# Effects of Diactive‐1–Supported Progressive Resistance Training on Body Composition in Youth With Type 1 Diabetes

**DOI:** 10.1002/jcsm.70257

**Published:** 2026-03-19

**Authors:** Jacinto Muñoz‐Pardeza, José Francisco López‐Gil, Ignacio Hormazábal‐Aguayo, Mikel Izquierdo, Cesar Agostinis‐Sobrinho, Yasmin Ezzatvar, Antonio García‐Hermoso

**Affiliations:** ^1^ Navarrabiomed Hospital Universitario de Navarra (HUN), Universidad Pública de Navarra (UPNA), IdiSNA Pamplona Spain; ^2^ School of Medicine Universidad Espíritu Santo Samborondón Ecuador; ^3^ Faculty of Health Sciences Universidad Autónoma de Chile Temuco Chile; ^4^ Vicerrectoría de Investigación y Postgrado Universidad de La Serena La Serena Chile; ^5^ CIBER of Frailty and Healthy Aging (CIBERFES) Instituto de Salud Carlos III Madrid Spain; ^6^ Health Research and Innovation Science Center Klaipeda University Klaipėda Lithuania; ^7^ Sport Physical Activity and Health Research & Innovation Center Polytechnic Institute of Guarda Guarda Portugal; ^8^ Lifestyle Factors With Impact on Ageing and Overall Health (LAH) Research Group, Departamento de Enfermería Universitat de València Valencia Spain; ^9^ Vicerrectoría de Investigación y Postgrado Universidad de Los Lagos Osorno Chile

**Keywords:** childhood, diabetic myopathy, insulin‐dependent diabetes, musculoskeletal health, strength training

## Abstract

**Background:**

Compared to their healthy peers, children and adolescents with type 1 diabetes are at an increased risk of adverse changes in body composition, including increased fat mass along with reductions in lean and bone mass. Although exercise has shown promise in improving body mass index in this population, the individual effects of resistance training on specific body composition parameters remain understudied. The aim of the study was to evaluate the effects of resistance training supported by the mHealth application Diactive‐1 on body composition in children and adolescents with type 1 diabetes.

**Methods:**

Sixty‐two participants with type 1 diabetes (aged 8–18 years old; 48% females) participated in a 24‐week randomised controlled trial and were assigned to either the usual care group (*n* = 32) or the exercise group (*n* = 30). The intervention was delivered via the Diactive‐1 app, which generates progressive overload resistance training programmes tailored to real‐time glycaemia and provides educational support. Body composition was assessed using anthropometry and dual‐energy X‐ray absorptiometry, with fat, lean and bone measurements standardised by age, sex and ethnicity. Linear mixed models were used to evaluate between‐group differences in change over time under both intention‐to‐treat (ITT) and per‐protocol (PP) approaches.

**Results:**

At 24 weeks, in the ITT analysis, the intervention group showed greater gains in lean mass (mean difference [MD] = 0.88 kg; 95% confidence interval [CI] 0.09 to 1.66; Hedges' *g* = 0.568) and whole‐body bone mineral content less head (MD = 32.40 g; 95% CI 6.90 to 57.89; *g* = 0.644) compared with those in the usual care group. No changes were observed in anthropometric measures, fat mass–related regions or standardised variables (*p* > 0.05). The risk of probable sarcopenia was lower in the intervention group (relative risk [RR] = 0.17; 95% CI 0.04 to 0.73; Cohen's *h* = 0.987) than in the usual care group. Findings were directionally consistent in the PP analysis.

**Conclusions:**

This intervention increased bone‐related outcomes and was associated with modest gains in lean mass and a lower risk of probable sarcopenia in youths with type 1 diabetes. These findings highlight the potential of the Diactive‐1 app as an adjunct tool to support musculoskeletal health in youths with type 1 diabetes.

**Trial Registration:**

ClinicalTrials.gov identifier: NCT06048757

## 1 Introduction

Type 1 diabetes is characterised by autoimmune destruction of pancreatic β‐cells, leading to progressive loss of insulin production [1]. Its incidence is increasing by 3%–4% annually [2], with 14.07 cases per 100 000 youths [3]. Although body mass index (BMI) and waist circumference are widely employed in clinical practice due to their association with health risks [4], they do not directly quantify body composition. In youths with type 1 diabetes, body fat has been reported to be approximately 2.3% greater than in their apparently healthy counterparts [5]. However, despite the musculoskeletal impairments observed in this population compared with their healthy peers [6], complications such as diabetic myopathy [7], a sarcopenia‐like condition characterised by loss of muscle mass and function [8], remain insufficiently investigated. In this context, muscle fibre atrophy has been observed [9], with a limited capacity for reversal [7], potentially contributing to metabolic dysfunction [10]. Decreases in bone strength, as evidenced by decreased areal bone mineral density (aBMD) and bone mineral content (BMC) [11], have been associated with an increased risk of fracture [12]. For this purpose, the American Diabetes Association (ADA) recommends monitoring bone health using dual‐energy X‐ray absorptiometry (DXA) in people with type 1 diabetes [13].

The ADA recommends musculoskeletal strengthening activities at least three times per week [14], aligning with the World Health Organization [15]. Such activities increase muscular strength, which is linked to reduced body fat [16] and greater BMC and aBMD across multiple anatomical sites in 83 children and adolescents with type 1 diabetes [17]. A meta‐analysis revealed that concurrent training (i.e., aerobic and resistance exercise) reduces BMI among young people with type 1 diabetes [18]; however, the individual effects of resistance training on BMI, fat mass and lean mass remain unexplored. With respect to bone health, a randomised controlled trial (RCT) including 14 children with type 1 diabetes revealed that a 9‐month training programme attenuated aBMD loss [19]. Nevertheless, this intervention combined resistance training with sports and did not assess outcomes related to BMC. A barrier to achieving physical activity adherence is the fear of hypoglycaemia [20]; however, the common use of smartphones offers an opportunity to deliver accessible tools that empower youths and promote self‐management in type 1 diabetes. The resistance training educational sessions supported by the mHealth application Diactive‐1 have already been shown to reduce insulin doses, with an effective algorithm for increasing muscle fitness [21]. This fits within a broader paradigm in type 1 diabetes management in which novel insulin analogues, adjunctive noninsulin therapies and integrated technologies are being explored to address insulin resistance and metabolic dysfunction [22].

A secondary analysis of a previously registered RCT (ClinicalTrials.gov: NCT06048757) was conducted to (a) evaluate the effects of resistance training delivered via the Diactive‐1 app on BMI, waist circumference, fat mass, lean mass and bone parameters in children and adolescents with type 1 diabetes; (b) assess these effects via standardised values relative to a reference population; and (c) determine the potential risk of sarcopenia after the intervention. We hypothesised that the programme would increase bone health and lean mass, while reducing the risk of sarcopenia.

## 2 Methods

### 2.1 Setting, Participants and Procedures

This parallel‐group RCT followed CONSORT 2025 guidelines (Table S1), the Template for Intervention Description and Replication, the Consensus on Exercise Reporting Template and the CHecklist for statistical Assessment of Medical Papers to ensure methodological transparency. Ethical approval was obtained (PI_2021/144), and informed consent was provided by participants and guardians in accordance with research standards.

The project was promoted through leaflets, although the Paediatric Endocrinology Unit coordinated recruitment at the University Hospital of Navarra (Spain). Eligible participants were males and females aged 8–18 years with type 1 diabetes who had been diagnosed for more than 6 months, were fluent in Spanish and were available to participate. The exclusion criteria included comorbidities limiting physical activity (e.g., cardiovascular disease or severe obesity), the honeymoon phase (i.e., insulin ≤ 0.50 U/kg/day–glycosylated haemoglobin [HbA_1c_] ≤ 6%) [23] and inability to install the Diactive‐1 app due to a lack of internet or mobile phone access. The RCT was conducted between 17 October 2023 and 9 September 2024. Appointments were scheduled by phone for the initial (Week 0), intermediate (Week 12) and final (Week 24) assessments.

Finally, a total of 62 children and adolescents with type 1 diabetes were enrolled across the six recruitment waves [24].

### 2.2 Randomisation and Masking

Participants received alphanumeric identifiers and were randomised (1:1) via block allocation by a principal investigator using Research Randomizer (version 4). In the Diactive‐1 and usual care groups, 30 and 32 participants were assigned, respectively. The paediatrician, who had access to clinical data for safety reasons, informed the patients of their assignments.

Data collectors and analysts were blinded to group assignments, although participants were aware of their allocation due to the nature of the intervention.

### 2.3 Interventions

#### 2.3.1 Usual Care Group

Participants received routine clinical care without being given any special information about lifestyle. They were placed on a waiting list to access the Diactive‐1 app and the materials after the intervention period.

#### 2.3.2 Diactive‐1 Group

The intervention programme consisted of resistance training sessions supported by the Diactive‐1 application over a 24‐week period. Before starting, the participants attended a face‐to‐face session led by a physical educator to demonstrate movement patterns and application use, which had previously been reported to have high usability (score: 4.33/5.00) [25].

Based on baseline handgrip strength, a marker of overall muscular strength [26], participants were initially stratified into three training levels using age‐ and sex‐specific European reference percentiles derived from the FitBack project [27]: low (≤ 20th percentile), medium (21st percentile–79th percentile) and high (≥ 80th percentile). Details of the assessment protocol are available elsewhere [24]. The training load was tailored and progressively increased in terms of volume (number of exercises, sets and repetitions) and intensity (exercise complexity and resistance applied to the equipment used). In practical terms, each session typically included 4–5 resistance exercises performed for 3–4 sets of 6–12 repetitions, with progressive overload achieved by increasing sets, repetitions and/or external resistance. The participants could select from equipment‐based sessions (i.e., adjustable‐weight aqua balls and resistance bands), which alternated between upper limb, lower limb and core exercises; equipment‐free full‐body sessions; or paired training. The sessions could be performed in any location, facilitated by the accessibility of the mHealth app. To enhance engagement via gamification, participants earned points that determined their progression through levels and ranking.

The interstitial glucose, as well as the corresponding trend arrow, reported by the insulin pump or continuous glucose monitor, was entered into the application both before and after each training session, providing educational guidelines in accordance with those provided by Moser et al. [28]. The procedure for recording glucose levels after sessions and the continuous monitoring of heart rate during the sessions via the Polar Verity Sense device (Polar Electro, Kempele, Finland) ensured accurate documentation of the training sessions and adherence to the intervention. The progressive weekly increase in training load is presented alongside maximum heart rate and weekly average interstitial glucose concentrations around exercise (Figure S1).

### 2.4 Outcomes and Measurements

Assessments (i.e., baseline, Week 12 and Week 24) were conducted at the youth‐friendly lab. For each time point, the pre‐intervention assessment was performed within the 24 h preceding the first intervention session, and the post‐intervention assessment was performed within the 24 h following the final intervention session at Weeks 12 and 24, respectively. Each participant evaluation was performed in a single morning session lasting approximately 90 min, to minimise circadian variability in the outcomes.

#### 2.4.1 Socio‐Economic Status

It was assessed using the Family Affluence Scale‐III [29].

#### 2.4.2 Parameters Related to Diabetes

Disease duration (i.e., time since diabetes diagnosis), data pump use and HbA_1c_ were obtained from medical records. Fasting blood samples were analysed in the hospital lab. Glycaemic parameters (i.e., time in range, interstitial glucose, glycaemic coefficient of variation, glycaemic risk index and hypoglycaemic episodes) and insulin requirements were collected as appropriate using LibreView or CareLink software linked to FreeStyle2 continuous glucose monitors or insulin pump devices, respectively. Data reflected participants' existing diabetes management systems, following the calibration prespecified by their health professionals.

#### 2.4.3 Anthropometric Parameters and Peak Height Velocity (PHV)

Height was measured using a SECA 123 stadiometer (Hamburg, Germany), and weight was recorded in light clothing using a SECA 869 scale. The BMI (kg/m^2^) and the waist‐to‐height ratio (WHtR; score) were calculated. Somatic maturation was estimated in years from PHV using the Moore equation [30]. The pubertal stage was classified as follows: prepubertal (≤ −1 year to PHV), peripubertal (−1 to 1 year to PHV) and postpubertal (≥ 1 year to PHV) [30].

#### 2.4.4 DXA

Participants were assessed in a fasting state via a single DXA (Lunar iDXA, GE HealthCare, Chicago, IL, USA), using the enCORE.v18 software. In accordance with the International Society for Clinical Densitometry, the device was calibrated daily, and participants were instructed to remain in the supine position [31].

The scanner focused on the whole body to determine body composition. Additionally, fat and lean mass data were obtained from specific regions such as the arms, legs and trunk. In contrast, BMC and aBMD data were processed from the total body less head (TBLH), as well as from the arms, legs, pelvis and spine. The appendicular lean mass index (ALMI; kg/m^2^) was calculated.

Additionally, age‐, sex‐ and race/ethnicity‐specific *z*‐scores for fat and lean mass in the whole body, as well as for aBMD and BMC at TBLH, were calculated using the reference‐population mean and standard deviation (SD) derived from the BMD in Childhood Study reference dataset [32]. The same method was used to calculate the ALMI *z*‐score [33].

#### 2.4.5 Status of Sarcopenia

According to the European Working Group on Sarcopenia in Older People criteria [8], sarcopenia status was defined as follows (Figure S2): ‘without sarcopenia’ (handgrip strength > percentile 20), ‘probable sarcopenia’ (handgrip strength ≤ percentile 20; ALMI *z*‐score > −1.5 SD) or ‘confirmed sarcopenia’ (handgrip strength ≤ percentile 20; ALMI *z*‐score ≤ −1.5 SD).

### 2.5 Statistical Analysis

All statistical analyses were performed using RStudio (version 2024.09.1; RStudio, Boston, MA, USA). The *p* value was obtained from two‐sided tests, and significance was set at *p* < 0.05.

The assumption of normality was assessed using quantile–quantile plots and the Shapiro–Wilk test. The homogeneity of variance was evaluated using Levene's test. Descriptive statistics are presented as means with SDs for continuous variables and as frequencies with proportions for categorical variables.

For normally distributed data, independent‐sample *t*‐tests or one‐way analysis of variance were used, as appropriate. For non‐normally distributed variables, the Mann–Whitney *U*‐test or the Kruskal–Wallis *H*‐test was used. Differences were examined between pubertal stages, sex and insulin pump use, as well as between participants who voluntarily withdrew from the study and those who completed it.

To evaluate changes from baseline (Week 0) to post‐intervention (Week 24), considering the intermediate time point (Week 12) when appropriate, linear mixed‐effects models were adjusted for both intention‐to‐treat (ITT) and per‐protocol (PP) approaches (i.e., those who completed the full 24 weeks of the project). These models included ‘group × time’ point interactions as fixed effects and subject identification as a random effect to account for intra‐individual variability. Modelling was performed using the ‘*lme4*’ package. Residual normality and homoscedasticity were examined using graphical diagnostics, whereas the performance of the model was assessed using the ‘*performance*’ package. Unstandardised coefficients with 95% confidence intervals (CIs) were calculated to estimate changes within and between groups over time using the ‘*emmeans*’ package. For greater clinical interpretability, the percentage change was calculated in conjunction with effect size estimation. Hedges' *g* was used to account for small sample bias, with interpretations based on thresholds of 0.2, 0.5 and 0.8 SDs for small, medium and large effects, respectively [34]. Data loss (4.2%) was assessed via the ‘*naniar*’ package and was assumed to be random, applying the MCAR test. The missing values were not imputed to preserve the integrity of the RCT and because the models allowed the available observations to be included without bias. Sensitivity analyses replicated the primary models with additional adjustment for disease duration to assess the impact of the baseline imbalance between groups. Effect modification by sex and pubertal status was also examined. A restricted longitudinal data analysis (cLDA) was conducted using the ‘*LMMstar*’ package, constraining baseline outcome means to be equal across groups and specifying fixed effects for time × treatment (treatment coded as 0 for both groups at baseline and as Diactive‐1 or usual care at 12 and 24 weeks) to estimate the effect under a common baseline assumption [35]. In line with contemporary methodological recommendations for longitudinal RCTs [36], these models were considered supportive rather than primary; accordingly, the principal results are derived from the prespecified linear mixed‐effects models estimating between‐group differences in change over time.

To investigate the changes in sarcopenia, the proportions of transitions between sarcopenia statuses over time were assessed using a Sankey diagram. In addition, the relative risk (RR) estimates with 95% CIs and the number needed to treat the transition from no sarcopenia to probable sarcopenia were calculated. The effect size was estimated using Cohen's *h* based on arcsine‐transformed proportions.

## 3 Results

### 3.1 Study Participants

**FIGURE 1:**
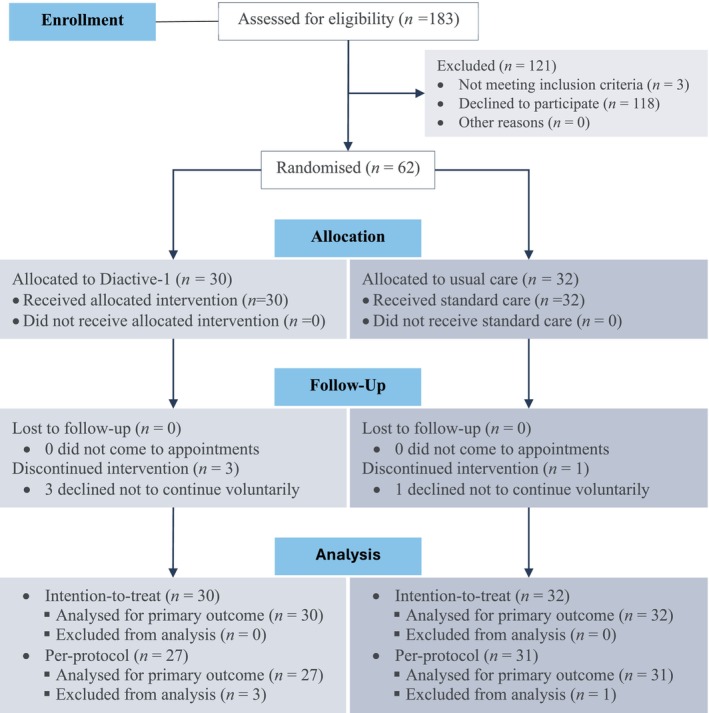
CONSORT 2025 flow diagram.

Sixty‐two patients with type 1 diabetes (aged 14.2 ± 2.5 years; females: 48%; disease duration: 5.7 ± 3.5 years; HbA_1c_: 60.4 ± 13.8 mmol/mol) provided informed consent to participate (Figure 1). In the intervention group, three participants did not complete the final assessment because they chose to withdraw voluntarily. Participants completed 74.2 ± 40.2 sessions, with an average of 2.9 ± 1.3 sessions per week. Eighty‐one per cent and 39% of the participants completed at least two and three sessions per week, respectively. One participant from the usual care group did not complete the final assessment. All baseline participants were included in the ITT analysis, and 58 (93%) met the criteria for the PP analysis.

Baseline characteristics (Tables 1 and S2) indicated that randomisation resulted in largely comparable groups; however, a difference in diabetes duration was observed between groups at baseline (*p* = 0.015). For the variables of interest, descriptive parameters are presented at baseline and at 12 and 24 weeks (Table S3). No statistically significant differences were observed between groups at any assessment point when the raw data were examined using a cross‐sectional analytical approach. Significant differences in waist circumference, fat mass, lean mass, BMC and aBMD were observed across the pubertal stages at baseline (Table S4; *p* < 0.05). Fat mass also differed according to sex (Table S5; *p* < 0.05). No differences were found between insulin pump users and non‐users or between study completers and withdrawals (Tables S6 and S7; *p* > 0.05). No adverse situations worthy of mention occurred during the study.

**TABLE 1:** Baseline characteristics of all participants and groups in the randomised controlled trial.

Variables	All (*n* = 62)	Diactive‐1 (*n* = 30)	Usual care (*n* = 32)
Demographic data			
Age (years)	14.25 ± 2.53	13.62 ± 2.41	14.83 ± 2.53
Diabetes duration (years)	5.76 ± 3.54	4.74 ± 3.41	6.69 ± 3.45
Socio‐economic level	8.60 ± 1.98	8.86 ± 2.06	8.35 ± 1.90
Sex			
Male, *n* (%)	32 (52)	15 (52)	17 (53)
Female, *n* (%)	30 (48)	14 (48)	16 (47)
Insulin pump			
Yes, *n* (%)	36 (57)	14 (45)	22 (69)
No, *n* (%)	26 (43)	16 (55)	10 (31)
Maturational status			
Peak height velocity (score)	0.54 ± 1.85	0.11 ± 1.75	0.92 ± 1.89
Prepubertal, *n* (%)	6 (10)	4 (17)	2 (6)
Peripubertal, *n* (%)	32 (51)	16 (50)	16 (50)
Postpubertal, *n* (%)	24 (39)	10 (33)	14 (44)
Diabetes‐related assessment			
Glycated haemoglobin (mmol/mol)	60.40 ± 13.80	58.73 ± 9.57	61.91 ± 16.77
Glycated haemoglobin (%)	7.6 ± 1.2	7.5 ± 0.8	7.8 ± 1.5
Time in range (%)	56.8 ± 20.8	55.8 ± 20.2	57.6 ± 21.7
Insulin doses (U/kg/day)	0.8 ± 0.2	0.9 ± 0.2	0.8 ± 0.2
Muscle strength			
Handgrip (reference *z*‐score)	−0.8 ± 0.6	−0.8 ± 0.7	−0.9 ± 0.6
Handgrip strength (kg)	23.04 ± 8.33	21.70 ± 7.92	24.26 ± 8.47
Anthropometry			
Height (m)	1.61 ± 0.14	1.59 ± 0.13	1.63 ± 0.14
Weight (kg)	55.39 ± 15.94	54.12 ± 16.27	56.54 ± 15.80
Body mass index (kg/m^2^)	20.88 ± 3.78	20.85 ± 3.70	20.90 ± 3.91
Waist circumference (cm)	68.88 ± 8.58	69.15 ± 9.49	68.64 ± 7.82
Waist‐to‐height ratio (score)	0.42 ± 0.04	0.43 ± 0.04	0.42 ± 0.04
Body composition by DXA			
Fat mass (kg)	15.89 ± 6.95	15.70 ± 7.90	16.06 ± 6.10
Fat mass (reference *z*‐score)	0.50 ± 0.85	0.43 ± 1.05	0.57 ± 0.64
Fat mass (%)	28.18 ± 7.28	28.01 ± 7.80	28.34 ± 6.90
Lean mass (kg)	37.62 ± 11.12	36.37 ± 10.40	38.76 ± 11.79
Lean mass (reference *z*‐score)	−0.81 ± 1.10	−0.64 ± 1.12	−0.97 ± 1.07
Sarcopenia (score)			
No sarcopenia, *n* (%)	27 (43)	13 (42)	14 (44)
Sarcopenia probable, *n* (%)	23 (37)	11 (38)	12 (37)
Sarcopenia confirmed, *n* (%)	12 (20)	6 (20)	6 (19)
Bone mineral content (g)	2049.89 ± 559.69	1977.71 ± 542.23	2115.30 ± 575.72
TBLH (reference *z*‐score)	0.85 ± 1.00	1.04 ± 1.01	0.67 ± 0.98
Areal bone mineral density (g/cm^2^)	1.04 ± 0.14	1.02 ± 0.14	1.05 ± 0.15
TBLH (reference *z*‐score)	0.51 ± 1.24	0.70 ± 1.11	0.35 ± 1.34

*Note:* Values are presented as means ± standard deviations. Age‐ and sex‐specific handgrip strength *z*‐scores are presented using reference data from FitBack network. Age‐, sex‐ and race/ethnicity‐specific body composition *z*‐scores are presented using reference data from the BMD in Childhood Study.

### 3.2 Intervention Effect

#### 3.2.1 ITT

No significant differences in anthropometry or fat mass were detected between the Diactive‐1 and usual care groups (Table 2). However, a significant between‐group difference in lean mass was detected at 24 weeks, favouring the Diactive‐1 group (mean difference [MD] = 0.88 kg; 95% CI 0.09 to 1.66; *g* = 0.568). With respect to BMC, increases were observed in the Diactive‐1 group compared to the usual care group, both at 12 weeks (MD = 26.74 g; 95% CI 1.13 to 52.34; *g* = 0.529) and at 24 weeks (MD = 33.55 g; 95% CI 7.39 to 59.71; *g* = 0.650). Region‐specific analyses (Table S8) revealed significant BMC increases in TBLH at 12 weeks (MD = 26.62 g; 95% CI 1.66 to 51.57; *g* = 0.540) and 24 weeks (MD = 32.40 g; 95% CI 6.90 to 57.89; *g* = 0.644) and in the pelvis at 24 weeks (MD = 12.36 g; 95% CI 2.87 to 21.84; *g* = 0.660). The aBMD improved in the legs at 12 weeks (MD = 0.02 g/cm^2^; 95% CI 0.01 to 0.03; *g* = 1.013) and 24 weeks (MD = 0.03 g/cm^2^; 95% CI 0.01 to 0.04; *g* = 1.519) and in the pelvis at 24 weeks (MD = 0.03 g/cm^2^; 95% CI 0.01 to 0.04; *g* = 1.519). Conversely, aBMD in the arms decreased in the Diactive‐1 group compared with the usual care group at 24 weeks (MD = −0.04 g/cm^2^; 95% CI −0.08 to −0.01; *g* = −0.506). No between‐group differences in the reference‐population *z*‐scores were found at 12 or 24 weeks (Table S10).

**TABLE 2:** Within‐group and between‐group differences (Diactive‐1 and usual care) resulting from intention‐to‐treat models at baseline, 12 and 24 weeks.

Variables	Within‐group differences (Diactive‐1; *n* = 30)			Within‐group differences (usual care; *n* = 32)			Between‐group differences		
	Change (95% CI)	Change %^a^	*p*	Change (95% CI)	Change %^a^	*p*	Mean difference (95% CI)	Hedges' *g*	*p*
Anthropometric									
BMI (kg/m^2^)									
Baseline to 24 weeks	0.53 (0.11 to 0.95)	1.272	**0.014**	0.31 (−0.07 to 0.69)	1.107	0.114	0.22 (−0.34 to 0.79)	0.197	0.433
WC (cm)									
Baseline to 12 weeks	0.88 (−0.87 to 2.63)	2.540	0.320	0.76 (−0.85 to 2.37)	1.482	0.349	0.12 (−2.26 to 2.50)	0.026	0.920
Waist‐to‐height ratio (score)									
Baseline to 24 weeks	0.00 (−0.01 to 0.01)	0.000	0.770	0.00 (−0.01 to 0.01)	0.000	0.912	0.00 (−0.01 to 0.01)	0.000	0.772
Body composition (DXA)									
Fat mass (kg)									
Baseline to 12 weeks	0.42 (−0.33 to 1.18)	2.675	0.387	0.34 (−0.37 to 1.05)	2.115	0.495	0.08 (−0.78 to 0.95)	0.047	0.851
12 to 24 weeks	0.12 (−0.67 to 0.92)	0.764	0.926	−0.19 (−0.90 to 0.53)	−1.182	0.814	0.31 (−0.58 to 1.20)	0.176	0.493
Baseline to 24 weeks	0.55 (−0.23 to 1.33)	3.503	0.225	0.15 (−0.56 to 0.87)	0.933	0.867	0.39 (−0.49 to 1.28)	0.223	0.383
Fat mass (%)									
Baseline to 12 weeks	−0.56 (−1.45 to 0.33)	−1.999	0.297	−0.77 (−1.61 to 0.06)	−2.716	0.075	0.21 (−0.80 to 1.23)	0.105	0.677
12 to 24 weeks	0.25 (−0.68 to 1.18)	0.892	0.801	−0.01 (−0.85 to 0.83)	−0.035	0.999	0.26 (−0.79 to 1.30)	0.126	0.627
Baseline to 24 weeks	−0.31 (−1.23 to 0.60)	−1.106	0.699	−0.78 (−1.63 to 0.06)	−2.751	0.076	0.47 (−0.57 to 1.51)	0.229	0.371
Lean mass (kg)									
Baseline to 12 weeks	2.33 (1.65 to 2.99)	6.404	**< 0.001**	1.81 (1.17 to 2.43)	4.669	**< 0.001**	0.52 (−0.25 to 1.28)	0.344	0.187
12 to 24 weeks	−0.07 (−0.77 to 0.62)	−0.192	0.967	−0.44 (−1.07 to 0.20)	−1.135	0.237	0.36 (−0.42 to 1.15)	0.232	0.363
Baseline to 24 weeks	2.25 (1.56 to 2.94)	6.184	**< 0.001**	1.37 (0.73 to 2.01)	3.534	**< 0.001**	0.88 (0.09 to 1.66)	0.568	**0.028**
BMC (g)									
Baseline to 12 weeks	77.60 (55.20 to 99.99)	3.923	**< 0.001**	50.86 (29.87 to 71.84)	2.404	**< 0.001**	26.74 (1.13 to 52.34)	0.529	**0.041**
12 to 24 weeks	48.92 (25.52 to 72.30)	2.473	**< 0.001**	42.10 (20.85 to 63.34)	1.990	**< 0.001**	6.81 (−19.54 to 33.17)	0.131	0.609
Baseline to 24 weeks	126.52 (103.45 to 149.57)	6.397	**< 0.001**	92.96 (71.71 to 114.20)	4.394	**< 0.001**	33.55 (7.39 to 59.71)	0.650	**0.012**
aBMD (g/cm^2^)									
Baseline to 12 weeks	0.02 (0.00 to 0.03)	1.941	**< 0.001**	0.02 (0.01 to 0.02)	1.886	**0.001**	0.00 (−0.01 to 0.01)	0.000	0.802
12 to 24 weeks	0.01 (−0.00 to 0.02)	0.970	0.282	0.01 (−0.01 to 0.02)	0.943	0.075	0.00 (−0.01 to 0.01)	0.000	0.728
Baseline to 24 weeks	0.03 (0.01 to 0.04)	2.912	**< 0.001**	0.03 (0.01 to 0.04)	2.830	**< 0.001**	0.00 (−0.01 to 0.01)	0.000	0.979

*Note:* The results are presented as the mean difference for each group and the mean difference between the groups. Data were analysed using linear‐mixed models. Bold values indicate significant differences.

Abbreviations: aBMD, areal body mass density; BMC, body mass content; BMI, body mass index; CI, confidence interval; WC, waist circumference.

^a^ The percentage change (corresponding assessment minus baseline values, divided by baseline values × 100) was calculated.

#### 3.2.2 PP

Findings were directionally consistent with the ITT analysis (Table S9). Similarly, no differences were observed in reference‐population *z*‐scores (Table S11).

#### 3.2.3 Sarcopenia

**FIGURE 2:**
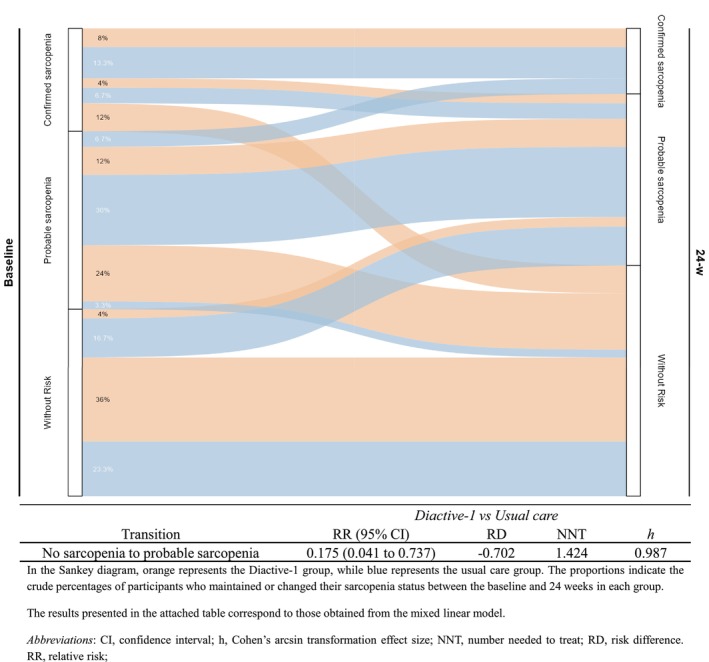
Estimated effect of the Diactive‐1 intervention on the risk transition between sarcopenia states at 24 weeks.

As shown in the Sankey plot (Figure 2), the intervention was associated with a more favourable 24‐week trajectory of sarcopenia status. Furthermore, when the risk of progression from non‐sarcopenia to probable sarcopenia was compared between groups at 24 weeks, the Diactive‐1 group had a lower risk (RR = 0.175; 95% CI 0.041 to 0.737; *h* = 0.987), with 1.42 participants needed to prevent one case of sarcopenia onset.

### 3.3 Sensitivity Analyses

When the analyses were replicated with diabetes duration adjustment to account for baseline differences between groups, the associations remained consistent in both direction and statistical significance (Table S12). Interaction analyses (i.e., pump use or sex) did not reveal significant differences in the variables with previously reported significant effects (Tables S13 and S14). The cLDA models restricting the baseline to ensure strict equality between groups showed results that were directionally consistent with the primary analyses (Table S15), although the between‐group MD over time in lean mass at 24 weeks was attenuated and no longer significant (MD = 0.96 kg; 95% CI −0.01 to 1.93; *g* = 0.504).

## 4 Discussion

This RCT evaluated whether a resistance training programme supported by the Diactive‐1 app could improve multiple aspects of body composition and muscle health in children and adolescents with type 1 diabetes. The intervention increased lean mass and BMC at whole‐body and regional levels, whereas standardised scores remained unchanged when compared with reference population values. Notably, the programme also reduced the proportion of participants classified as having probable sarcopenia, suggesting a potential early protective effect against musculoskeletal impairment in this population. Therefore, this study supports further benefits of strength‐based activities in diabetes care [14].

To our knowledge, this is the first RCT to simultaneously assess lean mass, fat mass, bone outcomes and sarcopenia‐related indicators in children and adolescents with type 1 diabetes using a structured resistance training programme delivered through an mHealth platform. The role of muscular strength in bone health has been highlighted previously because of its positive association with BMC and aBMD in the same regions examined in our study [17]. Maggio et al. showed that resistance training attenuated aBMD loss in a sample of 14 youths (53% female) with type 1 diabetes [19]; however, our results also demonstrated BMC gains, even when considering maturation status. The discrepancies observed between BMC and aBMD in our study may be explained in part by the fact that aBMD is influenced by the concomitant expansion of bone area characteristic of the paediatric population, which can mitigate the detectable effect of an intervention [37]. The muscle–bone unit could explain our findings in BMC; according to Frost's theory, mechanical loads stimulate the activity of osteoblasts and osteoclasts, which promotes bone accumulation [38]. In this context, lean mass increased to a greater extent over time in the Diactive‐1 group than in usual care when adjusting for disease duration; however, this association was attenuated under a restrictive baseline‐constrained approach and should therefore be interpreted with caution and examined in future studies in which body composition is a primary outcome. Physiologically, resistance training promotes lean mass gain by stimulating protein synthesis pathways, activating satellite cell activity and releasing anabolic hormones via acute inflammation [39]. This may be relevant, as lean mass has been associated with lower HbA_1c_ and better lipid profiles in youths with type 1 diabetes [40]. Nevertheless, despite raw increases, standardised *z*‐scores derived from a reference population remained unchanged, suggesting that the observed gains may have been consistent with the growth patterns of the reference population. Additionally, the reduced risk of sarcopenia warrants attention, given its relevance in the context of muscle health in diabetes, which is characterised by impaired muscle development and a shift towards fibres with reduced mitochondrial function in type 1 diabetes [7].

In the literature, BMI is the most studied body composition parameter in individuals with type 1 diabetes, and it decreases after exercise intervention [18]. However, the effects of resistance training remain undocumented, and their combination with aerobic exercise has led to inconsistent results [18]. Similarly, most evidence on fat mass in youths with type 1 diabetes involves concurrent training [18]; our results show no fat mass change with resistance training alone. Therefore, further research is needed to study the effects of resistance training on fat mass, given its relationship with insulin requirement [5].

### 4.1 Clinical and Research Implications

This 24‐week mHealth‐supported resistance training programme produced small‐to‐moderate increases in lean mass and BMC (Hedges' *g* ≈ 0.5–0.6), corresponding to an average gain of approximately 0.88 kg of lean mass and 33.55 g of BMC, respectively. Lean mass responses were heterogeneous and modest in absolute terms; however, changes of this magnitude have been suggested to be potentially relevant in youths participating in resistance training, particularly when accompanied by parallel improvements in muscular strength and functional indicators [39]. These findings should be interpreted cautiously and confirmed in trials specifically powered for body composition outcomes. In our study, the modest increase in lean mass coincided with gains in handgrip strength and ALMI *z*‐scores, suggesting a functionally relevant enhancement. From a clinical perspective, however, the direct impact of these changes on long‐term outcomes (e.g., fracture risk, metabolic stability and functional status) remains uncertain and warrants longer term trials with post‐intervention follow‐up.

The potential of Diactive‐1 to mitigate musculoskeletal comorbidities is supported by its integration of resistance training with educational content aligned with current clinical exercise and glucose‐management guidelines [28], and this mHealth system may represent a feasible and scalable adjunct to clinical care, although future studies should evaluate long‐term adherence and economic impact. In line with recent calls to develop type 1 diabetes–specific combination therapies that integrate drugs, hormones and technology‐assisted interventions [22], Diactive‐1 may represent a nonpharmacological component of such comprehensive strategies.

### 4.2 Strengths and Limitations

This RCT has several strengths, including the implementation of a systematic intervention using Diactive‐1, which delivers tailored sessions targeting muscle fitness and could be particularly useful for this population. Body composition assessment was aligned with ADA recommendations by incorporating DXA as a monitoring tool [13]. Additionally, mixed‐linear models accounted for intra‐individual variability, improving the accuracy of both pragmatic and explanatory estimates via both ITT and PP approaches.

However, this study had several limitations. First, DXA scans were limited to the whole body, which may reduce the precision of region‐specific results compared to site‐specific scans or more advanced techniques such as peripheral quantitative computed tomography (pQCT). Second, despite significant improvements in lean mass and bone health, the clinical implications should be validated in longer term RCTs to check whether the effects are sustained over time. Third, the handgrip and ALMI *z*‐score criteria used to define sarcopenia are based on adult populations, and although they have been applied in younger individuals, there are no specific criteria established for this age group. Fourth, the age range may introduce variability in exercise responses. Although interactions were explored, the study was not powered to detect subgroup effects. Therefore, this approach deserves further study in large, stratified samples. Fifth, participants were recruited from a region with a modest cohort, highlighting the need to validate the results in other contexts or even consider the scalability of this intervention. Sixth, awareness of the resistance training benefits, combined with the lack of participant blinding, may have led both groups to perform additional training due to intrinsic motivation, thereby introducing potential performance bias. Seventh, although the resistance programme allowed flexibility in exercise type and timing to enhance real‐world applicability, this variability may have led to heterogeneous training loads, influencing the observed outcomes. Eighth, this study is a secondary analysis of an RCT that was originally powered to detect changes in total daily insulin dose, the primary endpoint of the trial [24], and no separate a priori power calculation was conducted for the body composition outcomes; therefore, the present findings should be considered hypothesis‐generating and interpreted with caution until replicated in larger, prospectively powered trials. Finally, residual confounding cannot be fully excluded, and future studies should incorporate predefined covariate‐adjustment strategies within their models.

## 5 Conclusion

This intervention was associated with gains in bone‐related outcomes and with modest increases in lean mass, suggesting potential relevance for sarcopenia‐related risk in youths with type 1 diabetes. These findings support the potential of Diactive‐1 as an adjunctive strategy to promote musculoskeletal health in this population.

## Funding

The authors declare that financial support was received for the research, authorship and/or publication of this article. This study was supported by Grants PI21/01238 and PI24/00829 from the Instituto de Salud Carlos III (Spain) and co‐funded by the European Union. In addition, a research employment contract was secured from the Instituto de Salud Carlos III in the name of Jacinto Muñoz‐Pardeza (FI22/00329).

## Ethics Statement

Patients and/or the public were not involved in the design, conduct, reporting or dissemination plans of this research.

## Conflicts of Interest

The authors declare no conflicts of interest.

## Supporting information


**Table S1:** CONSORT 2025 checklist to include when reporting a randomised trial.
**Table S2:** Baseline characteristics of all participants and in each group in specific body regions.
**Table S3:** Characteristics of all participants and in each group, in each time and in specific body regions.
**Table S4:** Baseline differences between the different pubertal stages.
**Table S5:** Baseline differences between boys and girls.
**Table S6:** Baseline differences between insulin pump users and non‐insulin pump users.
**Table S7:** Baseline differences between those who dropped out of the study and those who completed the 24‐week study.
**Table S8:** Within‐group and between‐group differences (Diactive‐1 and usual care) in specific body regions, resulting from intention‐to‐treat models at baseline, 12 weeks and 24 weeks.
**Table S9:** Within‐group and between‐group differences (Diactive‐1 and usual care) in overall and specific body regions resulting from per‐protocol models at baseline, 12 weeks and 24 weeks
**Table S10:** Within‐group and between‐group differences (Diactive‐1 and usual care) in standardised outcomes (*z*‐scores adjusted for sex, age, and ethnicity) from intention‐to‐treat models at baseline, 12 weeks, and 24 weeks
**Table S11:** Within‐group and between‐group differences (Diactive‐1 and usual care) in standardised outcomes (*z*‐scores adjusted for sex, age, and ethnicity) from per‐protocol models at baseline, 12 weeks, and 24 weeks
**Table S12:** Within‐group and between‐group differences (Diactive‐1 and usual care) resulting from linear mixed models at baseline, 12 weeks and 24 week, and adjusted for duration of diabetes since onset.
**Table S13:** Interaction of sex and maturation stage on both the effects within the Diactive‐1 exercise group and the effects of the Diactive‐1 exercise group versus the usual care group at 12 and 24 weeks using the intention‐to‐treat approach.
**Table S14:** Interaction of sex and maturation stage on both the effects within the Diactive‐1 exercise group and the effects of the Diactive‐1 exercise group versus the usual care group at 12 and 24 weeks using the per‐protocol approach.
**Table S15:** Within‐group and between‐group differences (Diactive‐1 and usual care) resulting from restricted longitudinal data models (cLDA), limiting the means of the baseline results so that they were equal in all groups.
**Figure S1:** Actual progression of the Diactive‐1 muscle strength training programme over the 24‐week period.
**Figure S2:** Algorithm for sarcopenia status classification according to EWGSOP2 criteria.

## Data Availability

Data collected during the study will be made available upon reasonable request and approval by the trial steering committee, which can be obtained by contacting the corresponding author. The study protocol, statistical analysis plan and other issues were previously published [24].
