# Supplementary material for: Effects of Diactive‐1–Supported Progressive Resistance Training on Body Composition in Youth With Type 1 Diabetes
Source: J Cachexia Sarcopenia Muscle. 2026 Mar 19;17(2):e70257. doi: 10.1002/jcsm.70257 (PMC13140196; doi:10.1002/jcsm.70257)
Supplement: Supplementary file 1 — Table S1: CONSORT 2025 checklist to include when reporting a randomised trial. Table S2: Baseline characteristics of all participants and in each group in specific body regions. Table S3: Characteristics of all participants and in each group, in each time and in specific body regions. Table S4: Baseline differences between the different pubertal stages. Table S5: Baseline differences between boys and girls. Table S6: Baseline differences between insulin pump users and non‐insulin pump users. Table S7: Baseline differences between those who dropped out of the study and those who completed the 24‐week study. Table S8: Within‐group and between‐group differences (Diactive‐1 and usual care) in specific body regions, resulting from intention‐to‐treat models at baseline, 12 weeks and 24 weeks. Table S9: Within‐group and between‐group differences (Diactive‐1 and usual care) in overall and specific body regions resulting from per‐protocol models at baseline, 12 weeks and 24 weeks Table S10: Within‐group and between‐group differences (Diactive‐1 and usual care) in standardised outcomes (z‐scores adjusted for sex, age, and ethnicity) from intention‐to‐treat models at baseline, 12 weeks, and 24 weeks Table S11: Within‐group and between‐group differences (Diactive‐1 and usual care) in standardised outcomes (z‐scores adjusted for sex, age, and ethnicity) from per‐protocol models at baseline, 12 weeks, and 24 weeks Table S12: Within‐group and between‐group differences (Diactive‐1 and usual care) resulting from linear mixed models at baseline, 12 weeks and 24 week, and adjusted for duration of diabetes since onset. Table S13: Interaction of sex and maturation stage on both the effects within the Diactive‐1 exercise group and the effects of the Diactive‐1 exercise group versus the usual care group at 12 and 24 weeks using the intention‐to‐treat approach. Table S14: Interaction of sex and maturation stage on both the effects within the Diactive‐1 exercise group and the effects of the [file JCSM-17-e70257-s001.docx]

**Electronic supplementary material (ESM) for:**

**Effects of Diactive-1–supported progressive resistance training on body composition in youth with type 1 diabetes**

Jacinto Muñoz-Pardeza, José Francisco López-Gil, Ignacio Hormazábal-Aguayo, Mikel Izquierdo, Cesar Agostinis-Sobrinho, Yasmin Ezzatvar, Antonio García-Hermoso^*^

[**Table ESM1.** 1](#_Toc220601244)

[**Table ESM2.** 3](#_Toc220601245)

[**Table ESM3.** 5](#_Toc220601246)

[**Table ESM4.** 7](#_Toc220601247)

[**Table ESM5.** 9](#_Toc220601248)

[**Table ESM6.** 11](#_Toc220601249)

[**Table ESM7.** 13](#_Toc220601250)

[**Table ESM8.** 15](#_Toc220601251)

[**Table ESM9.** 18](#_Toc220601252)

[**Table ESM10.** 22](#_Toc220601253)

[**Table ESM11.** 23](#_Toc220601254)

[**Table ESM12.** 24](#_Toc220601255)

[**Table ESM13.** 27](#_Toc220601256)

[**Table ESM14.** 35](#_Toc220601257)

[**Table ESM15.** 43](#_Toc220601258)

[**Figure ESM1.** 46](#_Toc220601259)

[**Figure ESM2.** 47](#_Toc220601260)

**^*^ Corresponding author**

Antonio García-Hermoso

🖰 [antonio.garciah@unavarra.es](mailto:antonio.garciah@unavarra.es) (+34 848 424 931)

🖂 Navarrabiomed, Hospital Universitario de Navarra, Universidad Pública de Navarra (UPNA), IdiSNA, Pamplona, Spain.

# **Table ESM1.** CONSORT 2025 checklist to include when reporting a randomised trial.

| **Section/Topic** | **Nº** | **CONSORT 2025 checklist item description** | **Page** |
| --- | --- | --- | --- |
| **Title and abstract** |  |  |  |
| Title and structured abstract | 1a | Identification as a randomized trial | 1 |
|  | 1b | Structured summary of the trial design, methods, results, and conclusions | 2 |
| **Open science** |  |  |  |
| Trial registration | 2 | Name of trial registry, identifying number (with URL) and date of registration | 2 |
| Protocol and statistical analysis plan | 3 | Where the trial protocol and statistical analysis plan can be accessed | 19 |
| Data sharing | 4 | Where and how the individual de-identified participant data (including data dictionary), statistical code and any other materials can be accessed | 17 |
| Funding and conflicts of interest | 5a | Sources of funding and other support (eg, supply of drugs), and role of funders in the design, conduct, analysis and reporting of the trial | 17 |
|  | 5b | Financial and other conflicts of interest of the manuscript authors | 17 |
| **Introduction** |  |  |  |
| Background and rationale | 6 | Scientific background and rationale | 5 |
| Objectives | 7 | Specific objectives related to benefits and harms | 6 |
| **Methods** |  |  |  |
| Patient and public involvement | 8 | Details of patient or public involvement in the design, conduct and reporting of the trial | 6 |
| Trial design | 9 | Description of trial design including type of trial (eg, parallel group or crossover), allocation ratio, and framework (for example, superiority, equivalence, non-inferiority or exploratory) | 6 |
| Changes of trial protocol | 10 | Important changes to the trial after it commenced including any outcomes or analyses that were not prespecified, with reason | NA |
| Trial setting | 11 | Settings (such as community or hospital) and locations (eg, countries or sites) where the trial was conducted | 6 |
| Eligibility criteria | 12a | Eligibility criteria for participants | 6 |
|  | 12b | If applicable, eligibility criteria for sites and for individuals delivering the interventions (eg, surgeons or physiotherapists) | NA |
| Intervention and comparator | 13 | Intervention and comparator with sufficient details to allow replication. If relevant, where additional materials describing the intervention and comparator (eg, intervention manual) can be accessed | 7-8 |
| Outcomes | 14 | Prespecified primary and secondary outcomes, including the specific measurement variable (eg, systolic blood pressure), analysis metric (for example, change from baseline, final value, time to event), method of aggregation (eg, median, proportion), and time point for each outcome | 8-9 |
| Harms | 15 | How harms were defined and assessed (eg, systematically or non-systematically) | 6-9 |
| Sample size | 16a | How sample size was determined, including all assumptions supporting the sample size calculation | Protocol  Ref. 25 |
|  | 16b | Explanation of any interim analyses and stopping guidelines | NA |
| Randomization: |  |  |  |
| Sequence generation | 17a | Who generated the random allocation sequence and the method used | 7 |
|  | 17b | Type of randomization and details of any restriction (eg, stratification, blocking and block size) | 7 |
| Allocation concealment mechanism | 18 | Mechanism used to implement the random allocation sequence (eg, central computer/telephone; sequentially numbered, opaque, sealed containers), describing any steps to conceal the sequence until interventions were assigned | 7 |
| Implementation | 19 | Whether the personnel who enrolled and those who assigned participants to the interventions had access to the random allocation sequence | 7 |
| Blinding | 20a | Who was blinded after assignment to interventions (eg, participants, care providers, outcome assessors, data analysts) | 7 |
|  | 20b | If blinded, how blinding was achieved and description of the similarity of interventions | 7 |
| Statistical methods | 21a | Statistical methods used to compare groups for primary and secondary outcomes, including harms | 10 |
|  | 21b | Definition of who is included in each analysis (eg, all randomized participants), and in which group | 10 |
|  | 21c | How missing data were handled in the analysis | 11 |
|  | 21d | Methods for any additional analyses (eg. subgroup and sensitivity analyses), distinguishing prespecified from post hoc | 11 |
| **Results** |  |  |  |
| Participant flow, including flow diagram | 22a | For each group, the numbers of participants who were randomly assigned, received intended intervention, and were analyzed for the primary outcome | 11 |
|  | 22b | For each group, losses and exclusions after randomization, together with reasons | 11-12 |
| Recruitment | 23a | Dates defining the periods of recruitment and follow-up for outcomes of benefits and harms | 6 |
|  | 23b | If relevant, why the trial ended or was stopped | NA |
| Intervention and comparator delivery | 24a | Intervention and comparator as they were actually administered (eg, where appropriate, who delivered the intervention/comparator, how participants adhered, whether they were delivered as intended (fidelity)) | 7 |
|  | 24b | Concomitant care received during the trial for each group | 7 |
| Baseline data | 25 | A table showing baseline demographic and clinical characteristics for each group | 11 and Table 1 |
| Number analysed, outcomes and estimation | 26 | For each primary and secondary outcome, by group:  the number of participants included in the analysis  the number of participants with available data at the outcome time point result for each group, and the estimated effect size and its precision (such as 95% confidence interval)  for binary outcomes, presentation of both absolute and relative effect size | 12-13 |
| Harms | 27 | All harms or unintended events in each group | 12 |
| Ancillary analyses | 28 | Any other analyses performed, including subgroup and sensitivity analyses, distinguishing pre-specified from post hoc | 13 |
| Discussion |  |  |  |
| Interpretation | 29 | Interpretation consistent with results, balancing benefits and harms, and considering other relevant evidence | 13-14 |
| Limitations | 30 | Trial limitations, addressing sources of potential bias, imprecision, generalisability, and, if relevant, multiplicity of analyses | 15 |

# **Table ESM2.** Baseline characteristics of all participants and in each group in specific body regions.

| Variables | **All** (*n*=62) | **Diactive-1** (*n*=30) | **Usual Care** (*n*=32) |
| --- | --- | --- | --- |
| **Body composition by DEXA** | | | |
| Fat mass (kg) |  |  |  |
| *Arms (kg)* | 1.82 ± 0.69 | 1.77 ± 0.78 | 1.87 ± 0.61 |
| *Legs (kg)* | 6.99 ± 2.87 | 6.79 ± 3.10 | 7.16 ± 2.69 |
| *Trunk (kg)* | 6.25 ± 3.46 | 6.32 ± 4.03 | 6.19 ± 2.92 |
| Visceral fat mass (g) | 134.78 ± 137.21 | 127.03 ± 163.27 | 141.56 ± 111.85 |
| Subcutaneous fat mass (g) | 694.80 ± 562.37 | 712.50 ± 632.37 | 679.31 ± 503.07 |
| Lean mass (kg) |  |  |  |
| *Arms (kg)* | 3.99 ± 1.61 | 3.76 ± 1.49 | 4.20 ± 1.71 |
| *Legs (kg)* | 13.25 ± 4.24 | 12.67 ± 3.86 | 13.77 ± 4.55 |
| *Trunk (kg)* | 17.33 ± 5.07 | 16.92 ± 4.84 | 17.70 ± 5.31 |
| Appendicular lean mass index (kg/m²) | 6.44 ± 1.28 | 6.32 ± 1.19 | 6.55 ± 1.37 |
| Bone mineral content (g) |  |  |  |
| *TBLH (g)* | 1629.25 ± 504.92 | 1566.87 ± 491.65 | 1685.77 ± 517.85 |
| *Arms (g)* | 254.66 ± 85.85 | 241.44 ± 80.49 | 266.64 ± 90.00 |
| *Legs (g)* | 791.74 ± 236.63 | 760.31 ± 227.58 | 820.21 ± 244.61 |
| *Pelvis (g)* | 255.01 ± 91.75 | 246.69 ± 88.78 | 262.55 ± 95.14 |
| *Spine (g)* | 133.36 ± 49.03 | 126.76 ± 48.76 | 139.34 ± 49.26 |
| Areal bone mineral density (g/cm^2^) |  |  |  |
| *TBLH (g/cm^2^)* | 0.93 ± 0.15 | 0.92 ± 0.15 | 0.95 ± 0.16 |
| *Arms (g/cm^2^)* | 0.70 ± 0.13 | 0.69 ± 0.12 | 0.72 ± 0.14 |
| *Legs (g/cm^2^)* | 1.11 ± 0.18 | 1.09 ± 0.17 | 1.13 ± 0.19 |
| *Pelvis (g/cm^2^)* | 0.93 ± 0.18 | 0.91 ± 0.17 | 0.94 ± 0.19 |
| *Spine (g/cm^2^)* | 0.88 ± 0.17 | 0.87 ± 0.17 | 0.90 ± 0.18 |
| Values are presented as means ± standard deviations. | | | |

# **Table ESM3.** Characteristics of all participants and in each group, in each time and in specific body regions.

| Variables | **Diactive-1** (*n*=30) | | | **Usual Care** (*n*=32) | | | ***P*-value** | | |
| --- | --- | --- | --- | --- | --- | --- | --- | --- | --- |
|  | **Baseline** | **12-w** | **24-w** | **Baseline** | **12-w** | **24-w** | **Baseline** | **12-w** | **24-w** |
| **Anthropometric** | | | | | | | | | |
| IMC | 20.85 ± 3.70 | *NA* | 21.07 ± 3.36 | 20.90 ± 3.91 | *NA* | 21.25 ± 4.20 | 0.957 | *NA* | 0.856 |
| WC | 69.15 ± 9.49 | *NA* | 69.00 ± 8.63 | 68.64 ± 7.82 | *NA* | 69.61 ± 7.93 | 0.819 | *NA* | 0.783 |
| WHtR | 43.43 ± 4.12 | *NA* | 42.87 ± 3.59 | 42.15 ± 4.24 | *NA* | 42.33 ± 4.78 | 0.143 | *NA* | 0.626 |
| **DEXA** | | | | | | | | | |
| Fat mass (kg) | 15.70 ± 7.90 | 16.56 ± 7.97 | 14.95 ± 6.71 | 16.06 ± 6.10 | 16.39 ± 6.89 | 15.63 ± 6.12 | 0.600 | 0.933 | 0.701 |
| *Arms (kg)* | 1.77 ± 0.78 | 1.82 ± 0.74 | 1.74 ± 0.69 | 1.87 ± 0.61 | 1.89 ± 0.73 | 1.82 ± 0.63 | 0.573 | 0.720 | 0.655 |
| *Legs (kg)* | 6.79 ± 3.10 | 7.1 ± 3.20 | 6.5 ± 2.70 | 7.16 ± 2.69 | 7.22 ± 2.96 | 7.04 ± 2.78 | 0.623 | 0.887 | 0.474 |
| *Trunk (kg)* | 6.32 ± 4.03 | 6.81 ± 4.08 | 5.9 ± 3.39 | 6.19 ± 2.92 | 6.45 ± 3.34 | 5.95 ± 2.86 | 0.893 | 0.715 | 0.955 |
| Fat mass (%) | 28.01 ± 7.80 | 28.13 ± 7.61 | 26.75 ± 7.56 | 28.34 ± 6.90 | 27.56 ± 6.71 | 27.22 ± 7.01 | 0.860 | 0.766 | 0.813 |
| *Arms (%)* | 30.80 ± 7.94 | 31.48 ± 7.59 | 29.79 ± 8.15 | 30.88 ± 8.96 | 30.63 ± 9 | 29.44 ± 9.23 | 0.972 | 0.697 | 0.880 |
| *Legs (%)* | 32.80 ± 7.77 | 32.55 ± 7.51 | 31.32 ± 7.77 | 33.18 ± 7.58 | 31.99 ± 7.3 | 32.13 ± 7.79 | 0.848 | 0.775 | 0.702 |
| *Trunk (%)* | 24.69 ± 9.63 | 25.04 ± 9.51 | 23.36 ± 8.96 | 25.05 ± 7.46 | 24.37 ± 7.48 | 23.76 ± 7.51 | 0.874 | 0.769 | 0.859 |
| Visceral fat (*g*) | 127.03 ± 163.27 | 156.96 ± 179.69 | 107.00 ± 80.53 | 141.56 ± 111.85 | 170.41 ± 134.3 | 152.52 ± 123.16 | 0.693 | 0.763 | 0.099 |
| Subcutaneous fat (*g*) | 712.50 ± 632.37 | 645.85 ± 637.66 | 634.15 ± 482.40 | 679.31 ± 503.07 | 690.48 ± 585.63 | 678.39 ± 563.31 | 0.824 | 0.788 | 0.750 |
| Lean mass (kg) | 36.37 ± 10.40 | 37.96 ± 9.66 | 37.88 ± 11.09 | 38.76 ± 11.79 | 40.55 ± 11.62 | 39.71 ± 11.65 | 0.523 | 0.357 | 0.554 |
| *Arms (kg)* | 3.76 ± 1.49 | 3.71 ± 1.28 | 4.02 ± 1.79 | 4.20 ± 1.71 | 4.22 ± 1.7 | 4.40 ± 1.84 | 0.289 | 0.204 | 0.443 |
| *Legs (kg)* | 12.67 ± 3.86 | 13.31 ± 3.70 | 13.18 ± 3.86 | 13.77 ± 4.55 | 14.50 ± 4.57 | 14.07 ± 4.46 | 0.310 | 0.278 | 0.429 |
| *Trunk (kg)* | 16.92 ± 4.84 | 17.92 ± 4.53 | 17.67 ± 5.25 | 17.70 ± 5.31 | 18.77 ± 5.23 | 18.18 ± 5.21 | 0.549 | 0.509 | 0.718 |
| BMC (g) | 1977.71 ± 542.23 | 2035.27 ± 527.44 | 2070.85 ± 574.33 | 2115.30 ± 575.72 | 2166.81 ± 582.09 | 2186.12 ± 576.70 | 0.341 | 0.370 | 0.462 |
| *TBLH (g)* | 1566.87 ± 491.65 | 1617.56 ± 478.75 | 1647.79 ± 521.57 | 1685.77 ± 517.85 | 1732.14 ± 522.32 | 1746.57 ± 515.96 | 0.361 | 0.387 | 0.485 |
| *Arms (g)* | 241.44 ± 80.49 | 242.34 ± 75.71 | 258.47 ± 87.23 | 266.64 ± 90.00 | 269.16 ± 86.22 | 279.19 ± 88.85 | 0.253 | 0.212 | 0.388 |
| *Legs (g)* | 760.31 ± 227.58 | 779.71 ± 222.91 | 784.89 ± 229.21 | 820.21 ± 244.61 | 837.09 ± 245.13 | 849.74 ± 244.31 | 0.325 | 0.354 | 0.315 |
| *Pelvis (g)* | 246.69 ± 88.78 | 262 ± 83.73 | 268.02 ± 98.27 | 262.55 ± 95.14 | 277.84 ± 100.79 | 270.73 ± 93.54 | 0.503 | 0.516 | 0.917 |
| *Spine (g)* | 126.76 ± 48.76 | 133.69 ± 51.60 | 138.62 ± 53.61 | 139.34 ± 49.26 | 144.06 ± 48.13 | 144.30 ± 47.68 | 0.320 | 0.434 | 0.682 |
| aBMD (g/cm^2^) | 1.02 ± 0.14 | 1.04 ± 0.15 | 1.05 ± 0.15 | 1.05 ± 0.15 | 1.08 ± 0.16 | 1.08 ± 0.14 | 0.426 | 0.427 | 0.492 |
| *TBLH (g/cm^2^)* | 0.92 ± 0.15 | 0.94 ± 0.15 | 0.94 ± 0.15 | 0.95 ± 0.16 | 0.97 ± 0.17 | 0.97 ± 0.15 | 0.459 | 0.469 | 0.527 |
| *Arms (g/cm^2^)* | 0.69 ± 0.12 | 0.69 ± 0.15 | 0.68 ± 0.14 | 0.72 ± 0.14 | 0.74 ± 0.15 | 0.74 ± 0.14 | 0.427 | 0.191 | 0.071 |
| *Legs (g/cm^2^)* | 1.09 ± 0.17 | 1.12 ± 0.17 | 1.13 ± 0.15 | 1.13 ± 0.19 | 1.15 ± 0.20 | 1.15 ± 0.18 | 0.384 | 0.501 | 0.697 |
| *Pelvis (g/cm^2^)* | 0.91 ± 0.17 | 0.95 ± 0.16 | 0.97 ± 0.19 | 0.94 ± 0.19 | 0.97 ± 0.19 | 0.96 ± 0.17 | 0.578 | 0.685 | 0.783 |
| *Spine (g/cm^2^)* | 0.87 ± 0.17 | 0.90 ± 0.18 | 0.91 ± 0.19 | 0.90 ± 0.18 | 0.93 ± 0.18 | 0.93 ± 0.17 | 0.452 | 0.503 | 0.652 |
| Values are presented as means ± standard deviations.  The *p*-value is derived from either the independent t-test or the Mann-Whitney U test.  Bold letters indicate significant differences. | | | | | | | | | |

# **Table ESM4.** Baseline differences between the different pubertal stages.

| Variables | **Prepuberal**  (*n* = 6) | **Peripuberal**  (*n* = 32) | **Postpuberal**  (*n* = 24) | *p-*value |
| --- | --- | --- | --- | --- |
| **Demographic data** | | | | |
| Age (years) | 10 ± 0.56 | 13.15 ± 1.69 | 16.59 ± 1.27 | **<0.001** |
| Diabetes duration (years) | 3.71 ± 1.60 | 5.50 ± 3.29 | 6.53 ± 4.01 | 0.483 |
| Socioeconomic level | 8.00 ± 3.39 | 8.64 ± 2.12 | 8.66 ± 1.46 | 0.812 |
| **Diabetes-related assessment** | | | | |
| Glycosylated haemoglobin (mmol/mol) | 60.43 ± 8.72 | 62.29 ± 17.01 | 57.87 ± 9.07 | 0.908 |
| Glycosylated haemoglobin (%) | 7.68 ± 0.79 | 7.85 ± 1.55 | 7.44 ± 0.83 |  |
| Time in range (%) | 52.20 ± 9.85 | 51.50 ± 21.92 | 64.83 ± 19.03 | 0.059 |
| Insulin doses (U/kg/day) | 1.07 ± 0.16 | 0.89 ± 0.29 | 0.77 ± 0.28 | 0.065 |
| **Muscular fitness** | | | | |
| Handgrip (reference z-score) | -0.91 ± 0.44 | -0.87 ± 0.70 | -0.87 ± 0.69 | 0.992 |
| Handgrip strength (kg) | 12.82 ± 0.78 | 19.82 ± 5.52 | 29.47 ± 7.50 | **<0.001** |
| **Anthropometric data** | | | | |
| Height (m) | 1.34 ± 0.02 | 1.56 ± 0.09 | 1.72 ± 0.09 | **<0.001** |
| Weight (kilogram) | 32.88 ± 4.58 | 51.91 ± 14.88 | 64.71 ± 11.95 | **<0.001** |
| Body mass index (kg/m^2^) | 18.22 ± 1.98 | 20.73 ± 4.28 | 21.64 ± 3.12 | 0.177 |
| Waist circumference (cm) | 60.66 ± 5.33 | 68.27 ± 9.67 | 71.42 ± 6.28 | **0.029** |
| Waist-to-height ratio (score) | 0.42 ± 0.03 | 0.44 ± 0.05 | 0.41 ± 0.03 | 0.404 |
| **Body composition by DEXA** | | | | |
| Fat mass (kg) | 9.69 ± 3.76 | 15.99 ± 8.16 | 17.04 ± 4.91 | **0.021** |
| *Fat mass (reference z score)* | 0.19 ± 0.94 | 0.56 ± 0.96 | 0.50 ± 0.70 | 0.684 |
| *Arms (kg)* | 1.23 ± 0.34 | 1.85 ± 0.81 | 1.91 ± 0.50 | **0.047** |
| *Legs (kg)* | 4.09 ± 1.37 | 7.02 ± 3.18 | 7.55 ± 2.34 | **0.009** |
| *Trunk (kg)* | 3.69 ± 2.09 | 6.31 ± 4.22 | 6.71 ± 2.21 | **0.044** |
| *Fat mass (%)* | 28.95 ± 8.64 | 29.52 ± 7.83 | 26.25 ± 6.02 | 0.247 |
| Visceral fat mass (g) | 94.80 ± 79.94 | 134.19 ± 159.85 | 143.87 ± 115.84 | 0.597 |
| Subcutaneous fat mass (g) | 414.80 ± 326.50 | 699.61 ± 661.04 | 746.91 ± 450.40 | 0.259 |
| Lean mass (kg) | 21.90 ± 2.47 | 34.01 ± 8.37 | 45.71 ± 9.34 | **<0.001** |
| *Lean mass (reference z score)* | -1.51 ± 0.43 | -0.88 ± 1.25 | -0.58 ± 0.92 | 0.202 |
| *Arms (kg)* | 2.02 ± 0.31 | 3.44 ± 1.10 | 5.14 ± 1.58 | **<0.001** |
| *Legs (kg)* | 7.42 ± 0.89 | 11.98 ± 3.24 | 16.16 ± 3.75 | **<0.001** |
| *Trunk (kg)* | 10.03 ± 1.33 | 15.62 ± 3.89 | 21.12 ± 3.95 | **<0.001** |
| Appendicular lean mass index (kg/m²) | 5.25 ± 0.67 | 6.16 ± 1.17 | 7.06 ± 1.25 | **0.001** |
| Sarcopenia |  |  |  |  |
| *No sarcopenia, n (%)* | 2 (25) | 13 (41) | 12 (50) | **0.006** |
| *Sarcopenia probable, n (%)* | 4 (75) | 11 (34) | 8 (33) | 0.200 |
| *Sarcopenia confirmed, n (%)* | 0 (0) | 8 (25) | 4 (17) | **0.018** |
| Bone mineral content (g) | 1170.82 ± 105.11 | 1861.60 ± 432.50 | 2484.08 ± 386.15 | **<0.001** |
| *TBLH (reference z score)* | 0.52 ± 0.59 | 0.96 ± 1.20 | 0.76 ± 0.76 | 0.567 |
| *TBLH (g)* | 851.45 ± 111.56 | 1460.07 ± 393.54 | 2016.85 ± 355.06 | **<0.001** |
| *Arms (g)* | 131.10 ± 11.26 | 222.67 ± 58.10 | 323.04 ± 70.93 | **<0.001** |
| *Legs (g)* | 416.69 ± 70.01 | 722.30 ± 180.38 | 962.45 ± 183.48 | **<0.001** |
| *Pelvis (g)* | 121.15 ± 16.52 | 225.50 ± 75.57 | 322.24 ± 66.28 | **<0.001** |
| *Spine (g)* | 62.48 ± 7.71 | 115.02 ± 39.48 | 172.58 ± 31.53 | **<0.001** |
| Areal bone mineral density (g/cm^2^) | 0.83 ± 0.05 | 1.00 ± 0.13 | 1.14 ± 0.10 | **<0.001** |
| *TBLH (reference z score)* | 0.13 ± 0.90 | 0.63 ± 1.40 | 0.44 ± 1.07 | 0.666 |
| *TBLH (g/cm^2^)* | 0.71 ± 0.07 | 0.89 ± 0.14 | 1.03 ± 0.10 | **<0.001** |
| *Arms (g/cm^2^)* | 0.54 ± 0.08 | 0.66 ± 0.11 | 0.79 ± 0.10 | **<0.001** |
| *Legs (g/cm^2^)* | 0.84 ± 0.07 | 1.07 ± 0.16 | 1.23 ± 0.12 | **<0.001** |
| *Pelvis (g/cm^2^)* | 0.67 ± 0.05 | 0.88 ± 0.16 | 1.04 ± 0.13 | **<0.001** |
| *Spine (g/cm^2^)* | 0.64 ± 0.02 | 0.83 ± 0.16 | 1.00 ± 0.12 | **<0.001** |
| Values are presented as means ± standard deviations.  The *p*-value is derived from either the independent t-test or the Mann-Whitney U test.  Bold letters indicate significant differences. | | | | |

# **Table ESM5.** Baseline differences between boys and girls.

| Variables | **Boys** (*n* = 32) | **Girls** (*n* = 30) | *p-*value |
| --- | --- | --- | --- |
| **Demographic data** | | | |
| Age (years) | 14.25 ± 2.67 | 14.26 ± 2.41 | 0.990 |
| Diabetes duration (years) | 5.21 ± 3.58 | 6.36 ± 3.47 | 0.133 |
| Socioeconomic level | 8.12 ± 1.97 | 9.14 ± 1.87 | 0.018 |
| **Maturational status** | | | |
| Peak height velocity (score) | 0.66 ± 1.39 | 0.62 ± 1.16 | 0.901 |
| **Diabetes-related assessment** | | | |
| Glycosylated haemoglobin (mmol/mol) | 59.91 ± 12.29 | 61.82 ± 15.40 | 0.597 |
| Glycosylated haemoglobin (%) | 7.55 ± 1.12 | 7.80 ± 1.40 |  |
| Time in range (%) | 2.18 ± 2.65 | 1.62 ± 1.87 | 0.485 |
| Insulin doses (Units/kilogram/day) | 0.86 ± 0.30 | 0.85 ± 0.27 | 0.927 |
| **Muscular fitness** | | | |
| Handgrip (reference z-score) | -1.04 ± 0.63 | -0.60 ± 0.68 | **0.043** |
| Handgrip strength (kilogram) | 24.74 ± 10.14 | 21.17 ± 5.00 | 0.278 |
| **Anthropometric data** | | | |
| Height (m) | 1.63 ± 0.16 | 1.58 ± 0.10 | 0.138 |
| Weight (kilogram) | 56.58 ± 18.40 | 54.07 ± 12.88 | 0.543 |
| Body mass index (kg/m^2^) | 20.49 ± 3.84 | 21.31 ± 3.73 | 0.404 |
| Waist circumference (cm) | 70.38 ± 8.92 | 67.23 ± 8.02 | 0.153 |
| Waist-to-height ratio (score) | 0.43 ± 0.03 | 0.42 ± 0.04 | 0.258 |
| **Body composition by DEXA** | | | |
| Fat mass (kg) | 14.24 ± 7.25 | 17.71 ± 6.23 | **0.011** |
| *Fat mass (reference z score)* | 0.59 ± 0.86 | 0.41 ± 0.85 | 0.413 |
| *Arms (kg)* | 1.63 ± 0.67 | 2.03 ± 0.66 | **0.017** |
| *Legs (kg)* | 6.20 ± 3.05 | 7.86 ± 2.43 | **0.003** |
| *Trunk (kg)* | 5.55 ± 3.57 | 7.03 ± 3.22 | **0.027** |
| *Fat mass (%)* | 24.57 ± 7.02 | 32.17 ± 5.27 | **<0.001** |
| Visceral fat mass (g) | 144.90 ± 165.17 | 123.96 ± 101.02 | 0.917 |
| Subcutaneous fat mass (g) | 555.22 ± 492.68 | 844.00 ± 601.42 | **0.031** |
| Lean mass (kg) | 40.51 ± 13.14 | 34.44 ± 7.35 | 0.131 |
| *Lean mass (reference z score)* | -0.89 ± 0.97 | -0.73 ± 1.24 | 0.576 |
| *Arms (kg)* | 4.52 ± 1.96 | 3.41 ± 0.80 | 0.074 |
| *Legs (kg)* | 14.33 ± 4.95 | 12.06 ± 2.93 | 0.084 |
| *Trunk (kg)* | 18.50 ± 5.99 | 16.03 ± 3.46 | 0.056 |
| Appendicular lean mass index (kg/m²) | 6.77 ± 1.45 | 6.08 ± 0.97 | 0.101 |
| Sarcopenia (score) |  |  |  |
| *No sarcopenia, n (%)* | 10 (31) | 16 (55) | 0.103 |
| *Sarcopenia probable, n (%)* | 14 (44) | 9 (31) | 0.447 |
| *Sarcopenia confirmed, n (%)* | 9 (25) | 5 (14) | 0.437 |
| Bone mineral content (g) | 2134.00 ± 644.79 | 1957.07 ± 440.40 | 0.220 |
| *TBLH (reference z score)* | 0.82 ± 0.86 | 0.87 ± 1.16 | 0.846 |
| *TBLH (g)* | 1703.50 ± 590.96 | 1547.31 ± 382.61 | 0.230 |
| *Arms (g)* | 270.60 ± 104.48 | 237.07 ± 55.65 | 0.128 |
| *Legs (g)* | 842.34 ± 278.23 | 735.89 ± 167.70 | 0.079 |
| *Pelvis (g)* | 265.26 ± 105.70 | 243.70 ± 73.60 | 0.363 |
| *Spine (g)* | 129.56 ± 53.65 | 137.56 ± 43.92 | 0.528 |
| Areal bone mineral density (g/cm^2^) | 1.04 ± 0.15 | 1.04 ± 0.14 | 0.875 |
| *TBLH (reference z score)* | 0.33 ± 1.08 | 0.72 ± 1.38 | 0.225 |
| *TBLH (g/cm^2^)* | 0.93 ± 0.17 | 0.93 ± 0.14 | 0.932 |
| *Arms (g/cm^2^)* | 0.70 ± 0.14 | 0.71 ± 0.11 | 0.792 |
| *Legs (g/cm^2^)* | 1.12 ± 0.20 | 1.11 ± 0.15 | 0.879 |
| *Pelvis (g/cm^2^)* | 0.93 ± 0.20 | 0.93 ± 0.16 | 0.914 |
| *Spine (g/cm^2^)* | 0.86 ± 0.17 | 0.91 ± 0.18 | 0.310 |
| Values are presented as means ± standard deviations.  The *p*-value is derived from either the independent t-test or the Mann-Whitney U test.  Bold letters indicate significant differences. | | | |

# **Table ESM6.** Baseline differences between insulin pump users and non-insulin pump users.

| Variables | **Insulin pump** (*n* = 36) | **Non-insulin pump** (*n* = 26) | *p-*value |
| --- | --- | --- | --- |
| **Demographic data** | | | |
| Age (years) | 14.39 ± 2.70 | 14.07 ± 2.31 | 0.621 |
| Diabetes duration (years) | 6.92 ± 3.56 | 4.20 ± 2.91 | **0.002**^*^ |
| Socioeconomic level | 8.64 ± 1.87 | 8.53 ± 2.15 | 0.909 |
| **Maturational status** | | | |
| Peak height velocity (score) | 0.80 ± 1.44 | 0.43 ± 0.99 | 0.273 |
| **Diabetes-related assessment** | | | |
| Glycosylated haemoglobin (mmol/mol) | 57.18 ± 9.76 | 64.73 ± 17.14 | **0.045**^*^ |
| Glycosylated haemoglobin (%) | 7.38 ± 0.89 | 8.07 ± 1.56 |  |
| Time in range (%) | 65.17 ± 16.45 | 45.53 ± 21.19 | **<0.001**^***^ |
| Insulin doses (U/kg/day) | 0.85 ± 0.24 | 0.86 ± 0.34 | 0.929 |
| **Muscular fitness** | | | |
| Handgrip (reference z-score) | -0.81 ± 0.62 | -0.96 ± 0.74 | 0.389 |
| Handgrip strength (kg) | 23.69 ± 7.96 | 22.18 ± 8.71 | 0.373 |
| **Anthropometric data** | | | |
| Height (m) | 1.62 ± 0.15 | 1.59 ± 0.11 | 0.482 |
| Weight (kilogram) | 55.18 ± 16.17 | 55.66 ± 15.94 | 0.909 |
| Body mass index (kg/m^2^) | 20.49 ± 3.42 | 21.40 ± 4.23 | 0.360 |
| Waist circumference (cm) | 67.88 ± 8.08 | 70.24 ± 9.20 | 0.291 |
| Waist-to-height ratio (score) | 0.41 ± 0.03 | 0.43 ± 0.04 | 0.078 |
| **Body composition by DEXA** | | | |
| Fat mass (kg) | 15.68 ± 6.84 | 16.16 ± 7.23 | 0.679 |
| *Fat mass (reference z score)* | 0.42 ± 0.88 | 0.61 ± 0.82 | 0.392 |
| *Arms (kg)* | 1.82 ± 0.70 | 1.82 ± 0.69 | 0.811 |
| *Legs (kg)* | 7.00 ± 2.97 | 6.97 ± 2.81 | 0.867 |
| *Trunk (kg)* | 6.04 ± 3.22 | 6.54 ± 3.82 | 0.627 |
| *Fat mass (%)* | 28.10 ± 7.44 | 28.30 ± 7.21 | 0.918 |
| Visceral fat mass (g) | 134.22 ± 149.88 | 135.56 ± 120.24 | 0.781 |
| Subcutaneous fat mass (g) | 646.54 ± 520.64 | 762.36 ± 620.73 | 0.528 |
| Lean mass (kg) | 37.59 ± 11.59 | 37.67 ± 10.69 | 0.959 |
| *Lean mass (reference z score)* | -0.85 ± 1.10 | -0.77 ± 1.12 | 0.778 |
| *Arms (kg)* | 4.04 ± 1.68 | 3.92 ± 1.54 | 0.879 |
| *Legs (kg)* | 13.28 ± 4.44 | 13.21 ± 4.04 | 0.867 |
| *Trunk (kg)* | 17.21 ± 5.23 | 17.49 ± 4.94 | 0.834 |
| Appendicular lean mass index (kg/m²) | 6.36 ± 1.20 | 6.56 ± 1.41 | 0.712 |
| Sarcopenia (score) |  |  |  |
| *No sarcopenia, n (%)* | 17 (49) | 9 (35) | 0.407 |
| *Sarcopenia probable, n (%)* | 12 (34) | 11 (42) | 0.709 |
| *Sarcopenia confirmed, n (%)* | 6 (17) | 7 (23) | 0.801 |
| Bone mineral content (g) | 2065.99 ± 587.02 | 2028.21 ± 531.33 | 0.796 |
| *TBLH (reference z score)* | 0.79 ± 1.05 | 0.92 ± 0.95 | 0.640 |
| *TBLH (g)* | 1640.03 ± 523.92 | 1614.73 ± 488.02 | 0.848 |
| *Arms (g)* | 259.83 ± 89.49 | 247.69 ± 81.90 | 0.588 |
| *Legs (g)* | 796.70 ± 252.65 | 785.05 ± 217.92 | 0.851 |
| *Pelvis (g)* | 256.01 ± 92.88 | 253.66 ± 92.01 | 0.922 |
| *Spine (g)* | 133.68 ± 48.12 | 132.93 ± 51.18 | 0.953 |
| Areal bone mineral density (g/cm^2^) | 1.04 ± 0.14 | 1.04 ± 0.15 | 0.969 |
| *TBLH (reference z score)* | 0.42 ± 1.23 | 0.65 ± 1.26 | 0.478 |
| *TBLH (g/cm^2^)* | 0.93 ± 0.15 | 0.93 ± 0.16 | 0.938 |
| *Arms (g/cm^2^)* | 0.70 ± 0.13 | 0.71 ± 0.13 | 0.894 |
| *Legs (g/cm^2^)* | 1.12 ± 0.18 | 1.11 ± 0.17 | 0.800 |
| *Pelvis (g/cm^2^)* | 0.93 ± 0.17 | 0.93 ± 0.19 | 0.936 |
| *Spine (g/cm^2^)* | 0.88 ± 0.16 | 0.89 ± 0.19 | 0.752 |
| Values are presented as means ± standard deviations.  The *p*-value is derived from either the independent t-test or the Mann-Whitney U test.  Bold letters indicate significant differences. | | | |

# **Table ESM7.** Baseline differences between those who dropped out of the study and those who completed the 24-week study.

| Variables | **Dropout** (*n* = 4) | **24-weeks ended** (*n* = 58) | *p-*value |
| --- | --- | --- | --- |
| **Demographic data** | | | |
| Age (years) | 13.43 ± 1.51 | 14.31 ± 2.58 | 0.506 |
| Diabetes duration (years) | 6.20 ± 3.28 | 5.73 ± 1.98 | 0.651 |
| Socioeconomic level | 8.75 ± 2.21 | 8.5 ± 2.06 | 0.880 |
| **Maturational status** | | | |
| Peak height velocity (score) | 0.01 ± 0.67 | 0.69 ± 1.30 | 0.309 |
| **Diabetes-related assessment** | | | |
| Glycosylated haemoglobin (mmol/mol) | 60.38 ± 16.43 | 60.40 ± 13.77 | 0.599 |
| Glycosylated haemoglobin (%) | 7.67 ± 1.50 | 7.67 ± 1.26 |  |
| Time in range (%) | 51.50 ± 33.76 | 57.17 ± 20.10 | 0.861 |
| Insulin doses (U/kg/day) | 0.65 ± 0.41 | 0.87 ± 0.27 | 0.146 |
| **Muscular fitness** | | | |
| Handgrip (reference z-score) | -1.00 ± 0.44 | -0.86 ± 0.68 | 0.701 |
| Handgrip strength (kg) | 20.15 ± 3.47 | 23.25 ± 8.46 | 0.662 |
| **Anthropometric data** | | | |
| Height (m) | 165.15 ± 8.42 | 160.99 ± 14.42 | 0.572 |
| Weight (kilogram) | 63.02 ± 19.53 | 54.85 ± 15.72 | 0.325 |
| Body mass index (kg/m^2^) | 22.72 ± 5.18 | 20.75 ± 3.69 | 0.319 |
| Waist circumference (cm) | 75.00 ± 17.08 | 68.45 ± 7.78 | 0.142 |
| Waist-to-height ratio (score) | 0.45 ± 0.08 | 0.42 ± 0.03 | 0.827 |
| **Body composition by DEXA** | | | |
| Fat mass (kg) | 23.32 ± 11.84 | 15.37 ± 6.33 | 0.149 |
| *Fat mass (reference z score)* | 1.17 ± 1.29 | 0.45 ± 0.81 | 0.106 |
| *Arms (kg)* | 2.46 ± 0.96 | 1.78 ± 0.66 | 0.126 |
| *Legs (kg)* | 9.77 ± 4.22 | 6.79 ± 2.70 | 0.175 |
| *Trunk (kg)* | 10.18 ± 6.57 | 5.98 ± 3.06 | 0.226 |
| *Fat mass (%)* | 34.59 ± 9.38 | 27.73 ± 7.00 | 0.068 |
| Visceral fat mass (g) | 319.00 ± 417.90 | 125.08 ± 108.10 | 0.519 |
| Subcutaneous fat mass (g) | 913.00 ± 995.37 | 683.315 ± 543.26 | 0.878 |
| Lean mass (kg) | 38.54 ± 7.18 | 37.56 ± 11.39 | 0.827 |
| *Lean mass (reference z score)* | -0.22 ± 0.23 | -0.86 ± 1.12 | 0.270 |
| *Arms (kg)* | 3.78 ± 0.59 | 4.01 ± 1.66 | 0.781 |
| *Legs (kg)* | 13.44 ± 2.89 | 13.24 ± 4.34 | 0.759 |
| *Trunk (kg)* | 18.04 ± 3.56 | 17.28 ± 5.17 | 0.774 |
| Appendicular lean mass index (kg/m²) | 6.25 ± 0.76 | 6.45 ± 1.32 | 0.988 |
| Sarcopenia (score) |  |  |  |
| *No sarcopenia, n (%)* | 2 (50) | 24 (42) | 0.991 |
| *Sarcopenia probable, n (%)* | 2 (50) | 21 (36) | 0.628 |
| *Sarcopenia confirmed, n (%)* | 0 (0) | 13 (22) | 0.576 |
| Bone mineral content (g) | 2080.55 ± 446.66 | 2047.74 ± 569.97 | 0.910 |
| *TBLH (reference z score)* | 1.43 ± 0.61 | 0.81 ± 1.02 | 0.230 |
| *TBLH (g)* | 1652.73 ± 376.38 | 1627.60 ± 515.29 | 0.924 |
| *Arms (g)* | 238.20 ± 37.94 | 255.81 ± 88.31 | 0.695 |
| *Legs (g)* | 849.70 ± 203.85 | 787.67 ± 239.81 | 0.616 |
| *Pelvis (g)* | 247.88 ± 62.93 | 255.51 ± 93.82 | 0.873 |
| *Spine (g)* | 128.66 ± 43.79 | 133.69 ± 49.71 | 0.844 |
| Areal bone mineral density (g/cm^2^) | 1.05 ± 0.11 | 1.04 ± 0.15 | 0.919 |
| *TBLH (reference z score)* | 1.19 ± 0.47 | 0.47 ± 1.26 | 0.263 |
| *TBLH (g/cm^2^)* | 0.95 ± 0.10 | 0.93 ± 0.16 | 0.841 |
| *Arms (g/cm^2^)* | 0.71 ± 0.08 | 0.70 ± 0.13 | 0.909 |
| *Legs (g/cm^2^)* | 1.13 ± 0.10 | 1.11 ± 0.18 | 0.811 |
| *Pelvis (g/cm^2^)* | 0.90 ± 0.12 | 0.93 ± 0.18 | 0.732 |
| *Spine (g/cm^2^)* | 0.92 ± 0.18 | 0.88 ± 0.18 | 0.687 |
| Values are presented as means ± standard deviations.  The *p*-value is derived from either the independent t-test or the Mann-Whitney U test. | | | |

# **Table ESM8.** Within-group and between-group differences (Diactive-1 and usual care) in specific body regions, resulting from intention-to-treat models at baseline, 12 weeks and 24 weeks.

|  | Within-group differences (Diactive-1; *n*=30) | | | Within-group differences (Usual care; *n*=32) | | | Between-groups differences | | |
| --- | --- | --- | --- | --- | --- | --- | --- | --- | --- |
| Variables | Change  (95% CI) | Change %^Ψ^ | *p*  value | Change  (95% CI) | Change %^Ψ^ | *p*  value | Mean difference  (95% CI) | Hedges' *g* | *p*  value |
| **Body composition (DXA)** | | | | | | | | | |
| Arm fat mass *(kg)* |  |  |  |  |  |  |  |  |  |
| *Baseline to 12-w* | 0.02 (-0.08 to 0.12) | 1.129 | 0.932 | 0.02 (-0.08 to 0.11) | 1.063 | 0.908 | 0.00 (-0.12 to 0.11) | 0.000 | 0.978 |
| *12-w to 24-w* | 0.08 (-0.03 to 0.18) | 4.519 | 0.201 | -0.02 (-0.12 to 0.07) | -1.063 | 0.862 | 0.10 (-0.02 to 0.22) | 0.422 | 0.107 |
| *Baseline to 24-w* | 0.09 (-0.01 to 0.20) | 5.084 | 0.096 | 0.00 (-0.10 to 0.09) | 0.000 | 0.993 | 0.10 (-0.02 to 0.22) | 0.422 | 0.110 |
| Leg fat mass *(kg)* |  |  |  |  |  |  |  |  |  |
| *Baseline to 12-w* | 0.13 (-0.14 to 0.41) | 1.911 | 0.483 | 0.07 (-0.18 to 0.33) | 0.976 | 0.793 | 0.06 (-0.25 to 0.38) | 0.096 | 0.691 |
| *12-w to 24-w* | 0.07 (-0.21 to 0.36) | 3.088 | 0.825 | 0.03 (-0.23 to 0.29) | 1.394 | 0.960 | 0.04 (-0.28 to 0.36) | 0.063 | 0.797 |
| *Baseline to 24-w* | 0.21 (-0.07 to 0.49) | 1.029 | 0.202 | 0.10 (-0.16 to 0.36) | 0.418 | 0.635 | 0.11 (-0.21 to 0.43) | 0.174 | 0.518 |
| Trunk fat mass *(kg)* |  |  |  |  |  |  |  |  |  |
| *Baseline to 12-w* | 0.26 (-0.18 to 0.71) | 4.113 | 0.344 | 0.25 (-0.17 to 0.66) | 4.032 | 0.348 | 0.02 (-0.49 to 0.53) | 0.020 | 0.945 |
| *12-w to 24-w* | -0.03 (-0.50 to 0.43) | -0.474 | 0.983 | 0.25 (-0.17 to 0.66) | -3.064 | 0.536 | 0.16 (-0.37 to 0.68) | 0.154 | 0.556 |
| *Baseline to 24-w* | 0.23 (-0.23 to 0.69) | 3.639 | 0.464 | 0.06 (-0.37 to 0.48) | 0.967 | 0.949 | 0.18 (-0.35 to 0.70) | 0.174 | 0.508 |
| Visceral fat (*g*) |  |  |  |  |  |  |  |  |  |
| *Baseline to 12-w* | 18.71 (-14.77 to 52.18) | 13.603 | 0.382 | 28.21 ( -1.89 to 58.31) | 19.927 | 0.071 | -9.51 (-47.05 to 28.04) | -0.128 | 0.616 |
| *12-w to 24-w* | -17.21 (-51.20 to 16.78) | -12.512 | 0.453 | -19.20 (-49.66 to 11.26) | -13.563 | 0.295 | 2.00 (-36.07 to 40.07) | 0.027 | 0.917 |
| *Baseline to 24-w* | 1.50 (-30.50 to 33.50) | 1.090 | 0.993 | 9.01 (-20.35 to 38.37) | 6.364 | 0.746 | -7.51 (-43.73 to 28.71) | -0.105 | 0.681 |
| Subcutaneous fat (*g*) |  |  |  |  |  |  |  |  |  |
| *Baseline to 12-w* | -93.99 (-200.14 to 12.16) | -12.460 | 0.093 | 1.13 ( -97.31 to 99.56) | 0.166 | 0.999 | -95.12 (-215.86 to 25.63) | -0.399 | 0.121 |
| *12-w to 24-w* | 50.97 ( -56.78 to 158.72) | 6.757 | 0.501 | -6.09 (-105.73 to 93.54) | -0.896 | 0.988 | 57.06 ( -65.34 to 179.47) | 0.236 | 0.357 |
| *Baseline to 24-w* | -43.02 (-147.69 to 61.65) | -5.703 | 0.593 | -4.97 (-100.96 to 91.02) | -0.731 | 0.991 | -38.05 (-156.51 to 80.41) | -0.163 | 0.525 |
| Arm lean mass *(kg)* |  |  |  |  |  |  |  |  |  |
| *Baseline to 12-w* | 0.11 (-0.08 to 0.29) | 2.917 | 0.372 | 0.00 (-0.17 to 0.17) | 0.000 | 0.999 | 0.11 (-0.10 to 0.31) | 0.272 | 0.326 |
| *12-w to 24-w* | 0.22 (0.02 to 0.41) | 5.835 | **0.022** | 0.21 (0.03 to 0.38) | 4.988 | **0.016** | 0.01 (-0.20 to 0.23) | 0.024 | 0.915 |
| *Baseline to 24-w* | 0.33 (0.13 to 0.51) | 8.753 | **0.001** | 0.21 (0.03 to 0.38) | 4.988 | **0.016** | 0.12 (-0.10 to 0.33) | 0.283 | 0.286 |
| Leg lean mass *(kg)* |  |  |  |  |  |  |  |  |  |
| *Baseline to 12-w* | 0.84 (0.52 to 1.16) | 6.624 | **<0.001** | 0.72 (0.41 to 1.01) | 5.224 | **<0.001** | 0.12 (-0.24 to 0.49) | 0.167 | 0.500 |
| *12-w to 24-w* | -0.07 (-0.40 to 0.25) | -0.552 | 0.855 | -0.23 (-0.53 to 0.07) | -1.669 | 0.176 | 0.15 (-0.22 to 0.53) | 0.203 | 0.418 |
| *Baseline to 24-w* | 0.77 (0.43 to 1.09) | 6.072 | **<0.001** | 0.49 (0.18 to 0.79) | 3.555 | **0.001** | 0.28 (-0.09 to 0.65) | 0.383 | 0.141 |
| Trunk lean mass *(kg)* |  |  |  |  |  |  |  |  |  |
| *Baseline to 12-w* | 1.35 (0.91 to 1.78) | 7.978 | **<0.001** | 1.08 (0.66 to 1.48) | 6.101 | **<0.001** | 0.28 (-0.22 to 0.77) | 0.286 | 0.278 |
| *12-w to 24-w* | -0.23 (-0.69 to 0.22) | -1.359 | 0.450 | -0.42 (-0.83 to -0.01) | -2.372 | **0.046** | 0.19 (-0.32 to 0.70) | 0.189 | 0.469 |
| *Baseline to 24-w* | 1.12 (0.66 to 1.56) | 6.619 | **<0.001** | 0.65 (0.23 to 1.07) | 3.672 | **0.001** | 0.46 (-0.04 to 0.97) | 0.461 | 0.075 |
| ALMI (*kg/m²)* |  |  |  |  |  |  |  |  |  |
| *Baseline to 24-w* | 0.23 (0.08 to 0.36) | 3.633 | **0.001** | 0.13 (0.01 to 0.25) | 1.984 | **0.047** | 0.10 (-0.08 to 0.28) | 0.281 | 0.294 |
| BMC-TBLH (*g*) |  |  |  |  |  |  |  |  |  |
| *Baseline to 12-w* | 70.79 (48.96 to 92.61) | 4.517 | **<0.001** | 44.17 (23.70 to 64.62) | 2.620 | **<0.001** | 26.62 (1.66 to 51.57) | 0.540 | **0.036** |
| *12-w to 24-w* | 41.79 (18.99 to 64.58) | 2.667 | **<0.001** | 36.01(15.29 to 56.72) | 2.136 | **<0.001** | 5.78 (-19.91 to 31.47) | 0.114 | 0.656 |
| *Baseline to 24-w* | 112.57 (90.09 to 135.05) | 7.184 | **<0.001** | 80.17 (59.46 to 100.88) | 4.755 | **<0.001** | 32.40 (6.90 to 57.89) | 0.644 | **0.013** |
| BMC-Arms (*g*) |  |  |  |  |  |  |  |  |  |
| *Baseline to 12-w* | 5.74 (-2.533 to 14.021) | 2.377 | 0.229 | 1.60 (-6.155 to 9.36) | 0.600 | 0.875 | 4.14 (-5.32 to 13.60) | 0.222 | 0.387 |
| *12-w to 24-w* | 15.60 (6.951 to 24.241) | 6.461 | **<0.001** | 11.53 (3.669 to 19.38) | 4.324 | **0.002** | 4.07 (-5.67 to 13.81) | 0.212 | 0.409 |
| *Baseline to 24-w* | 21.34 (12.816 to 29.863) | 8.838 | **<0.001** | 13.13 (5.275 to 20.98) | 4.924 | **<0.001** | 8.21 (-1.45 to 17.87) | 0.430 | 0.095 |
| BMC-Legs (*g*) |  |  |  |  |  |  |  |  |  |
| *Baseline to 12-w* | 28.68 (18.897 to 38.464) | 3.772 | **<0.001** | 15.05 (5.88 to 24.22) | 1.834 | **<0.001** | 13.63 (2.44 to 24.81) | 0.617 | **0.017** |
| *12-w to 24-w* | 19.35 (9.136 to 29.573) | 2.544 | **<0.001** | 23.41 (14.12 to 32.70) | 2.854 | **<0.001** | -4.06 (-15.57 to 7.45) | -0.179 | 0.486 |
| *Baseline to 24-w* | 48.03 (37.959 to 58.110) | 6.317 | **<0.001** | 38.47 (29.18 to 47.75) | 4.690 | **<0.001** | 9.57 ( -1.86 to 20.99) | 0.424 | 0.100 |
| BMC-Pelvis (*g*) |  |  |  |  |  |  |  |  |  |
| *Baseline to 12-w* | 20.29 (12.16 to 28.40) | 8.224 | **<0.001** | 15.26 (7.64 to 22.86) | 5.812 | **<0.001** | 5.03 (-4.25 to 14.31) | 0.275 | 0.284 |
| *12-w to 24-w* | 4.78 ( -3.70 to 13.26) | 1.937 | 0.376 | -2.54 (-10.24 to 5.16) | -0.967 | 0.714 | 7.32 (-2.23 to 16.88) | 0.388 | 0.131 |
| *Baseline to 24-w* | 25.07 (16.70 to 33.43) | 10.162 | **<0.001** | 12.71 (5.00 to 20.42) | 4.840 | **<0.001** | 12.36 (2.87 to 21.84) | 0.660 | **0.011** |
| BMC-Spine (*g*) |  |  |  |  |  |  |  |  |  |
| *Baseline to 12-w* | 7.55 (3.01 to 12.07) | 5.955 | **<0.001** | 6.03 (1.77 to 10.27) | 4.327 | 0.003 | 1.52 (-3.65 to 6.70) | 0.149 | 0.561 |
| *12-w to 24-w* | 3.49 (-1.24 to 8.21) | 2.753 | 0.191 | 2.08 (-2.22 to 6.37) | 1.492 | 0.487 | 1.41 (-3.92 to 6.74) | 0.134 | 0.601 |
| *Baseline to 24-w* | 11.03 (6.36 to 15.69) | 8.700 | **<0.001** | 8.10 (3.80 to 12.40) | 5.812 | **<0.001** | 2.93 (-2.36 to 8.22) | 0.281 | 0.274 |
| aBMD-TBLH (*g/cm^2^*) |  |  |  |  |  |  |  |  |  |
| *Baseline to 12-w* | 0.02 (0.01 to 0.02) | 2.173 | **<0.001** | -0.01 (-0.194 to 0.184) | 2.105 | **0.002** | 0.01 (-0.01 to 0.02) | 0.169 | 0.475 |
| *12-w to 24-w* | 0.01 (-0.01 to 0.02) | 1.086 | 0.422 | 0.01 (-0.01 to 0.02) | 1.052 | 0.089 | 0.00 (-0.01 to 0.01) | 0.000 | 0.620 |
| *Baseline to 24-w* | 0.03 (0.01 to 0.04) | 3.260 | **<0.001** | 0.03 (0.01 to 0.04) | 3.157 | **<0.001** | 0.00 (-0.01 to 0.01) | 0.000 | 0.841 |
| aBMD-Arms (*g/cm^2^*) |  |  |  |  |  |  |  |  |  |
| *Baseline to 12-w* | 0.00 (-0.03 to 0.03) | 0.000 | 0.999 | 0.02 (-0.01 to 0.05) | 2.777 | 0.302 | -0.02 (-0.05 to 0.01) | -0.338 | 0.317 |
| *12-w to 24-w* | -0.01 (-0.04 to 0.02) | -1.428 | 0.657 | 0.01 (-0.02 to 0.04) | 1.388 | 0.710 | -0.02 (-0.06 to 0.01) | -0.267 | 0.241 |
| *Baseline to 24-w* | -0.01 (-0.04 to 0.02) | -1.428 | 0.657 | 0.03 (-0.01 to 0.06) | 4.166 | 0.066 | -0.04 (-0.08 to -0.01) | -0.506 | **0.032** |
| aBMD-Legs (*g/cm^2^*) |  |  |  |  |  |  |  |  |  |
| *Baseline to 12-w* | 0.03 ( 0.015 to 0.047) | 2.727 | **<0.001** | 0.01 (-0.003 to 0.027) | 0.877 | 0.131 | 0.02 (0.01 to 0.03) | 1.013 | **0.045** |
| *12-w to 24-w* | 0.02 ( 0.005 to 0.039) | 1.818 | **0.005** | 0.02 ( 0.000 to 0.030) | 1.754 | 0.051 | 0.01 (-0.01 to 0.02) | 0.169 | 0.458 |
| *Baseline to 24-w* | 0.05 ( 0.037 to 0.070) | 4.545 | **<0.001** | 0.03 ( 0.012 to 0.043) | 2.631 | **0.001** | 0.03 (0.01 to 0.04) | 1.519 | **0.007** |
| aBMD-Pelvis (*g/cm^2^*) |  |  |  |  |  |  |  |  |  |
| *Baseline to 12-w* | 0.03 (0.01 to 0.04) | 3.260 | **<0.001** | 0.02 (0.01 to 0.03) | 2.105 | **0.002** | 0.01 (-0.01 to 0.03) | 0.253 | 0.111 |
| *12-w to 24-w* | 0.02 (0.01 to 0.03) | 2.173 | **0.014** | 0.00 (-0.01 to 0.01) | 0.000 | 0.726 | 0.01 (-0.01 to 0.03) | 0.253 | 0.112 |
| *Baseline to 24-w* | 0.05 (0.03 to 0.06) | 5.434 | **<0.001** | 0.02 (0.01 to 0.03) | 2.105 | **<0.001** | 0.03 ( 0.01 to 0.04) | 1.519 | **0.001** |
| aBMD-Spine (*g/cm^2^*) |  |  |  |  |  |  |  |  |  |
| *Baseline to 12-w* | 0.03 (0.01 to 0.05) | 3.448 | **<0.001** | 0.03 (0.01 to 0.05) | 3.296 | **<0.001** | 0.00 (-0.02 to 0.01) | 0.000 | 0.991 |
| *12-w to 24-w* | 0.01 (-0.01 to 0.02) | 1.149 | 0.747 | 0.01 (-0.01 to 0.02) | 1.098 | 0.412 | 0.00 (-0.02 to 0.01) | 0.000 | 0.750 |
| *Baseline to 24-w* | 0.04 (0.02 to 0.05) | 4.597 | **<0.001** | 0.04 (0.02 to 0.05) | 4.395 | **<0.001** | 0.00 (-0.02 to 0.01) | 0.000 | 0.741 |
| The results are presented as the mean difference for each group and the mean difference between the groups. Data were analysed using linear-mixed models.  Bold letters indicate significant differences.  ^Ψ^The percentage change (corresponding assessment less baseline values/baseline values × 100) was calculated.  *Abbreviations*: CI, confidence interval; ALMI, appendicular lean mass index; BMC, body mass content; TBLH, total body less head; aBMD, areal body mass density. | | | | | | | | | |

# **Table ESM9.** Within-group and between-group differences (Diactive-1 and usual care) in overall and specific body regions resulting from per-protocol models at baseline, 12 weeks and 24 weeks

|  | Within-group differences (Diactive-1; *n*=27) | | | Within-group differences (Usual care; *n*=31) | | | Between-groups differences | | |
| --- | --- | --- | --- | --- | --- | --- | --- | --- | --- |
| Variables | Change  (95% CI) | Change %^Ψ^ | *p*  value | Change  (95% CI) | Change %^Ψ^ | *p*  value | Mean difference  (95% CI) | Hedges' *g* | *p*  value |
| **Anthropometric** | | | | | | | | | |
| BMI (*kg/m^2^*) |  |  |  |  |  |  |  |  |  |
| *Baseline to 24-w* | 0.55 (0.12 to 0.96) | 2.679 | **0.012** | 0.31 (-0.07 to 0.69) | 1.479 | 0.116 | 0.24 (-0.33 to 0.80) | 0.221 | 0.405 |
| WC (*cm*) |  |  |  |  |  |  |  |  |  |
| *Baseline to 24-w* | 1.03 (-0.71 to 2.78) | 1.515 | 0.241 | 0.73 (-0.87 to 2.33) | 1.059 | 0.366 | 0.31 (-2.06 to 2.68) | 0.069 | 0.797 |
| WHtR (score) |  |  |  |  |  |  |  |  |  |
| *Baseline to 24-w* | 0.00 (-0.011 to 0.010) | 0.000 | 0.916 | 0.00 (-0.010 to 0.010) | 0.000 | 0.962 | 0.00 (-0.015 to 0.014) | 0.000 | 0.912 |
| **Body composition (DEXA)** | | | | | | | | | |
| Fat mass (*kg*) |  |  |  |  |  |  |  |  |  |
| *Baseline to 12-w* | 0.48 (-0.32 to 1.27) | 3.296 | 0.334 | 0.34 (-0.37 to 1.05) | 2.118 | 0.498 | 0.13 (-0.75 to 1.02) | 0.076 | 0.766 |
| *12-w to 24-w* | 0.11 (-0.69 to 0.92) | 0.755 | 0.941 | -0.19 (-0.91 to 0.53) | -1.183 | 0.813 | 0.30 (-0.60 to 1.20) | 0.174 | 0.512 |
| *Baseline to 24-w* | 0.59 (-0.20 to 1.38) | 4.052 | 0.189 | 0.15 (-0.57 to 0.88) | 0.934 | 0.870 | 0.43 (-0.46 to 1.33) | 0.252 | 0.339 |
| Arm fat mass *(kg)* |  |  |  |  |  |  |  |  |  |
| *Baseline to 12-w* | 0.00 (-0.10 to 0.11) | 0.000 | 0.997 | 0.02 (-0.08 to 0.11) | 1.069 | 0.908 | -0.01 (-0.13 to 0.10) | -0.043 | 0.821 |
| *12-w to 24-w* | 0.09 (-0.02 to 0.19) | 5.389 | 0.145 | -0.02 (-0.12 to 0.07) | 1.069 | 0.993 | 0.11 (-0.01 to 0.23) | 0.464 | 0.082 |
| *Baseline to 24-w* | 0.09 (-0.01 to 0.20) | 5.389 | 0.118 | 0.00 (-0.10 to 0.09) | 0.000 | 0.861 | 0.10 (-0.02 to 0.22) | 0.426 | 0.125 |
| Leg fat mass *(kg)* |  |  |  |  |  |  |  |  |  |
| *Baseline to 12-w* | 0.15 (-0.14 to 0.43) | 2.351 | 0.450 | 0.07 (-0.19 to 0.33) | 0.979 | 0.793 | 0.08 (-0.24 to 0.40) | 0.129 | 0.644 |
| *12-w to 24-w* | 0.07 (-0.22 to 0.36) | 1.097 | 0.839 | 0.03 (-0.23 to 0.29) | 0.419 | 0.962 | 0.04 (-0.29 to 0.37) | 0.064 | 0.809 |
| *Baseline to 24-w* | 0.22 (-0.07 to 0.50) | 3.448 | 0.180 | 0.10 (-0.16 to 0.36) | 1.398 | 0.637 | 0.12 (-0.21 to 0.44) | 0.193 | 0.482 |
| Trunk fat mass *(kg)* |  |  |  |  |  |  |  |  |  |
| *Baseline to 12-w* | 0.31 (-0.15 to 0.78) | 5.429 | 0.251 | 0.25 (-0.17 to 0.67) | 4.025 | 0.350 | 0.07 (-0.45 to 0.59) | 0.070 | 0.799 |
| *12-w to 24-w* | -0.05 (-0.52 to 0.42) | -0.875 | 0.967 | -0.19 (-0.62 to 0.23) | -3.059 | 0.532 | 0.14 (-0.38 to 0.67) | 0.138 | 0.592 |
| *Baseline to 24-w* | 0.27 (-0.20 to 0.73) | 4.728 | 0.371 | 0.05 (-0.37 to 0.48) | 0.805 | 0.952 | 0.21 (-0.31 to 0.74) | 0.209 | 0.428 |
| Fat mass (*%*) |  |  |  |  |  |  |  |  |  |
| *Baseline to 12-w* | -0.45 (-1.374 to 0.483) | -1.664 | 0.491 | -0.77 (-1.612 to 0.064) | -2.717 | 0.076 | 0.33 (-0.71 to 1.37) | 0.166 | 0.533 |
| *12-w to 24-w* | 0.21 (-0.736 to 1.149) | 0.776 | 0.861 | -0.01 (-0.858 to 0.839) | -0.035 | 0.999 | 0.22 (-0.84 to 1.27) | 0.109 | 0.686 |
| *Baseline to 24-w* | -0.24 (-1.167 to 0.689) | -0.887 | 0.814 | -0.78 (-1.631 to 0.065) | -2.752 | 0.076 | 0.54 (-0.50 to 1.59) | 0.271 | 0.305 |
| Visceral fat (*g*) |  |  |  |  |  |  |  |  |  |
| *Baseline to 12-w* | 16.90 (-17.48 to 51.28) | 16.286 | 0.474 | 29.71 (-1.01 to 60.43) | 20.780 | 0.060 | -12.81 (-51.26 to 25.64) | -0.175 | 0.510 |
| *12-w to 24-w* | -13.67 (-48.0 to 20.7) | -13.173 | 0.612 | -20.16 (-50.88 to 10.56) | -14.100 | 0.267 | 6.49 (-31.96 to 44.94) | 0.089 | 0.738 |
| *Baseline to 24-w* | 3.23 (-29.08 to 35.55) | 3.112 | 0.969 | 9.55 (-20.05 to 39.14) | 6.679 | 0.723 | -6.32 (-42.86 to 30.23) | -0.091 | 0.732 |
| Subcutaneous fat (*g*) |  |  |  |  |  |  |  |  |  |
| *Baseline to 12-w* | -112.64 (-221.02 to -4.25) | -16.503 | **0.039** | 0.26 ( -99.82 to 100.33) | 0.038 | 1.000 | -112.89 (-235.93 to 10.14) | -0.482 | 0.071 |
| *12-w to 24-w* | 64.25 (-44.13 to 172.64) | 9.413 | 0.339 | -5.84 (-105.92 to 94.24) | -0.853 | 0.989 | 70.09 ( -52.95 to 193.13) | 0.299 | 0.261 |
| *Baseline to 24-w* | -48.38 (-153.58 to 56.81) | -7.088 | 0.520 | -5.58 (-101.92 to 90.76) | -0.815 | 0.989 | -42.80 (-161.77 to 76.16) | -0.189 | 0.477 |
| Lean mass (*kg*) |  |  |  |  |  |  |  |  |  |
| *Baseline to 12-w* | 2.23 (1.52 to 2.92) | 6.168 | **<0.001** | 1.81 (1.17 to 2.43) | 4.670 | **<0.001** | 0.42 (-0.36 to 1.20) | 0.282 | 0.294 |
| *12-w to 24-w* | -0.02 (-0.72 to 0.68) | -0.055 | 0.997 | -0.44 (-1.07 to 0.20) | -1.135 | 0.237 | 0.42 (-0.37 to 1.21) | 0.278 | 0.300 |
| *Baseline to 24-w* | 2.21 (1.50 to 2.90) | 6.113 | **<0.001** | 1.37 (0.73 to 2.00) | 3.535 | **<0.001** | 0.83 ( 0.04 to 1.62) | 0.553 | **0.038** |
| Arm lean mass *(kg)* |  |  |  |  |  |  |  |  |  |
| *Baseline to 12-w* | 0.07 (-0.12 to 0.26) | 1.856 | 0.678 | 0.00 (-0.17 to 0.17) | 0.000 | 1.000 | 0.07 (-0.14 to 0.28) | 0.171 | 0.5301 |
| *12-w to 24-w* | 0.24 (0.04 to 0.43) | 6.366 | **0.011** | 0.21 (0.03 to 0.38) | 4.976 | **0.015** | 0.03 (-0.18 to 0.25) | 0.072 | 0.7780 |
| *Baseline to 24-w* | 0.31 (0.11 to 0.49) | 8.222 | **0.001** | 0.21 (0.03 to 0.38) | 4.976 | **0.016** | 0.10 (-0.11 to 0.31) | 0.242 | 0.3641 |
| Leg lean mass *(kg)* |  |  |  |  |  |  |  |  |  |
| *Baseline to 12-w* | 0.79 (0.46 to 1.12) | 6.269 | **<0.001** | 0.72 (0.41 to 1.01) | 5.224 | **<0.001** | 0.08 (-0.29 to 0.45) | 0.112 | 0.680 |
| *12-w to 24-w* | -0.05 (-0.38 to 0.28) | -0.396 | 0.934 | -0.23 (-0.53 to 0.07) | -1.669 | 0.177 | 0.18 (-0.20 to 0.55) | 0.250 | 0.350 |
| *Baseline to 24-w* | 0.74 (0.41 to 1.07) | 5.873 | **<0.001** | 0.49 (0.18 to 0.79) | 3.555 | **0.001** | 0.26 (-0.11 to 0.63) | 0.363 | 0.178 |
| Trunk lean mass *(kg)* |  |  |  |  |  |  |  |  |  |
| *Baseline to 12-w* | 1.33 (0.87 to 1.79) | 7.921 | **<0.001** | 1.08 (0.66 to 1.48) | 6.105 | **<0.001** | 0.26 (-0.25 to 0.77) | 0.265 | 0.324 |
| *12-w to 24-w* | -0.22 (-0.68 to 0.24) | -1.310 | 0.494 | -0.42 (-0.84 to -0.01) | -2.374 | **0.048**^*^ | 0.20 (-0.32 to 0.72) | 0.201 | 0.451 |
| *Baseline to 24-w* | 1.11 (0.65 to 1.56) | 6.611 | **<0.001** | 0.65 (0.23 to 1.07) | 3.674 | **0.001** | 0.46 (-0.06 to 0.97) | 0.467 | 0.083 |
| ALMI (*kg/m²)* |  |  |  |  |  |  |  |  |  |
| *Baseline to 24-w* | 0.23 (0.08 to 0.36) | 3.627 | **0.001** | 0.13 (0.01 to 0.25) | 1.981 | **0.047** | 0.10 (-0.08 to 0.28) | 0.280 | 0.297 |
| BMC (*g*) |  |  |  |  |  |  |  |  |  |
| *Baseline to 12-w* | 74.22 (51.06 to 97.38) | 3.774 | **<0.001** | 50.86 (29.95 to 71.75) | 2.403 | **<0.001** | 23.37 ( -2.65 to 49.38) | 0.472 | 0.077 |
| *12-w to 24-w* | 50.68 (27.17 to 74.19) | 2.577 | **<0.001** | 92.96 (71.80 to 114.12) | 1.990 | **<0.001** | 8.58 (-17.80 to 34.95) | 0.171 | 0.520 |
| *Baseline to 24-w* | 124.91 (101.74 to 148.0) | 6.352 | **<0.001** | 42.11 (20.94 to 63.26) | 4.393 | **<0.001** | 31.94 (5.77 to 58.10) | 0.642 | **0.017** |
| BMC-TBLH (*g*) |  |  |  |  |  |  |  |  |  |
| *Baseline to 12-w* | 66.85 (44.36 to 89.33) | 4.297 | **<0.001** | 44.16 (23.86 to 64.45) | 2.616 | **<0.001** | 22.69 ( -2.57 to 47.95) | 0.472 | 0.077 |
| *12-w to 24-w* | 43.85 (21.02 to 66.67) | 2.818 | **<0.001** | 36.01 (15.46 to 56.55) | 2.133 | **<0.001** | 7.84 (-17.77 to 33.45) | 0.161 | 0.545 |
| *Baseline to 24-w* | 110.69 (88.20 to 133.18) | 7.115 | **<0.001** | 80.17 (59.62 to 100.71) | 4.749 | **<0.001** | 30.53 ( 5.12 to 55.93) | 0.632 | **0.019** |
| BMC-Arms (*g*) |  |  |  |  |  |  |  |  |  |
| *Baseline to 12-w* | 3.97 (-4.58 to 12.51) | 1.641 | 0.514 | 1.58 (-6.13 to 9.29) | 0.590 | 0.877 | 2.38 (-7.21 to 11.98) | 0.130 | 0.623 |
| *12-w to 24-w* | 16.52 (7.84 to 25.19) | 6.832 | **<0.001** | 11.53 (3.71 to 19.33) | 4.309 | **0.001** | 4.99 (-4.74 to 14.72) | 0.269 | 0.311 |
| *Baseline to 24-w* | 20.49 (11.93 to 29.03) | 8.473 | **<0.00**1 | 13.11 (5.30 to 20.92) | 4.899 | **0.001** | 7.38 (-2.28 to 17.03) | 0.402 | 0.133 |
| BMC-Legs (*g*) |  |  |  |  |  |  |  |  |  |
| *Baseline to 12-w* | 27.09 (16.92 to 37.25) | 3.628 | **<0.001** | 15.05 (5.87 to 24.21) | 1.830 | **<0.001** | 12.04 ( 0.62 to 23.45) | 0.554 | **0.039** |
| *12-w to 24-w* | 20.21 (9.88 to 30.52) | 2.706 | **<0.001** | 23.41 (14.12 to 32.70) | 2.847 | **<0.001** | -3.21 (-14.78 to 8.37) | -0.146 | 0.583 |
| *Baseline to 24-w* | 47.29 (37.12 to 57.45) | 6.333 | **<0.001** | 38.46 (29.17 to 47.74) | 4.678 | **<0.001** | 8.83 ( -2.65 to 20.31) | 0.404 | 0.130 |
| BMC-Pelvis (*g*) |  |  |  |  |  |  |  |  |  |
| *Baseline to 12-w* | 18.02 (9.87 to 26.16) | 7.292 | **<0.001** | 15.25 (7.90 to 22.60) | 5.807 | **<0.001** | 2.76 (-6.39 to 11.91) | 0.158 | 0.550 |
| *12-w to 24-w* | 5.96 (-2.31 to 14.22) | 2.412 | 0.205 | -2.54 (-9.98 to 4.90) | -0.967 | 0.697 | 8.49 (-0.78 to 17.77) | 0.481 | 0.072 |
| *Baseline to 24-w* | 23.98 (15.82 to 32.12) | 9.704 | **<0.001** | 12.72 (5.27 to 20.16) | 4.844 | **0.001** | 11.26 ( 2.05 to 20.46) | 0.643 | **0.017** |
| BMC-Spine (*g*) |  |  |  |  |  |  |  |  |  |
| *Baseline to 12-w* | 7.46 (2.81 to 12.11) | 5.802 | **0.001** | 6.05 (1.85 to 10.24) | 4.383 | **0.002** | 1.41 (-3.81 to 6.63) | 0.142 | 0.593 |
| *12-w to 24-w* | 3.51 (-1.20 to 8.23) | 2.730 | 0.185 | 2.08 (-2.17 to 6.32) | 1.507 | 0.478 | 1.43 (-3.86 to 6.73) | 0.142 | 0.592 |
| *Baseline to 24-w* | 10.98 (6.32 to 15.62) | 8.540 | **<0.001** | 8.13 (3.88 to 12.37) | 5.890 | **<0.001** | 2.84 (-2.41 to 8.09) | 0.284 | 0.285 |
| aBMD (*g/cm^2^*) |  |  |  |  |  |  |  |  |  |
| *Baseline to 12-w* | 0.02 (0.01 to 0.03) | 2.173 | **0.004** | 0.02 (0.01 to 0.02) | 2.105 | **0.001** | 0.00 (-0.01 to 0.01) | 0.000 | 0.954 |
| *12-w to 24-w* | 0.01 (-0.01 to 0.02) | 1.086 | 0.200 | 0.01 (-0.01 to 0.02) | 1.052 | 0.075 | 0.00 (-0.01 to 0.01) | 0.000 | 0.803 |
| *Baseline to 24-w* | 0.03 (0.01 to 0.03) | 3.260 | **<0.001** | 0.03 (0.01 to 0.04) | 3.157 | **<0.001** | 0.00 (-0.01 to 0.01) | 0.000 | 0.849 |
| aBMD-TBLH (*g/cm^2^*) |  |  |  |  |  |  |  |  |  |
| *Baseline to 12-w* | 0.02 (0.01 to 0.03) | 2.173 | **0.001** | 0.02 (0.01 to 0.02) | 2.105 | **0.002** | 0.00 (-0.01 to 0.01) | 0.000 | 0.700 |
| *12-w to 24-w* | 0.01 (-0.01 to 0.02) | 1.086 | 0.309 | 0.01 (-0.01 to 0.02) | 1.052 | 0.089 | 0.00 (-0.01 to 0.01) | 0.000 | 0.741 |
| *Baseline to 24-w* | 0.03 (0.01 to 0.04) | 3.260 | **<0.001** | 0.03 (0.01 to 0.04) | 3.157 | **<0.001** | 0.00 (-0.01 to 0.01) | 0.000 | 0.959 |
| aBMD-Arms (*g/cm^2^*) |  |  |  |  |  |  |  |  |  |
| *Baseline to 12-w* | 0.00 (-0.03 to 0.03) | 0.000 | 0.991 | 0.02 (-0.01 to 0.05) | 2.777 | 0.308 | -0.02 (-0.06 to 0.01) | -0.266 | 0.283 |
| *12-w to 24-w* | -0.01 (-0.04 to 0.02) | -1.449 | 0.720 | 0.01 (-0.02 to 0.04) | 1.388 | 0.713 | -0.02 (-0.06 to 0.01) | -0.250 | 0.274 |
| *Baseline to 24-w* | -0.01 (-0.04 to 0.02) | -1.449 | 0.635 | 0.03 (-0.01 to 0.06) | 4.166 | 0.069 | -0.04 (-0.08 to -0.01) | -0.489 | **0.031** |
| aBMD-Legs (*g/cm^2^*) |  |  |  |  |  |  |  |  |  |
| *Baseline to 12-w* | 0.03 (0.01 to 0.04) | 2.752 | **0.001** | 0.01 (-0.01 to 0.02) | 0.877 | 0.120 | 0.01 (-0.01 to 0.03) | 0.239 | 0.125 |
| *12-w to 24-w* | 0.02 (0.01 to 0.04) | 1.834 | **0.001** | 0.02 (0.01 to 0.03) | 1.754 | **0.044** | 0.01 (-0.01 to 0.02) | 0.277 | 0.314 |
| *Baseline to 24-w* | 0.05 (0.03 to 0.06) | 4.587 | **<0.001** | 0.03 (0.01 to 0.04) | 2.631 | **<0.001** | 0.02 ( 0.01 to 0.04) | 0.478 | **0.012** |
| aBMD-Pelvis (*g/cm^2^*) |  |  |  |  |  |  |  |  |  |
| *Baseline to 12-w* | 0.03 (0.01 to 0.04) | 3.260 | **<0.001** | 0.02 (0.01 to 0.03) | 2.105 | **0.001** | 0.01 (-0.01 to 0.02) | 0.263 | 0.197 |
| *12-w to 24-w* | 0.02 (0.01 to 0.03) | 2.173 | **0.001** | 0.00 (-0.01 to 0.01) | 0.000 | 0.719 | 0.02 (-0.01 to 0.03) | 0.457 | 0.081 |
| *Baseline to 24-w* | 0.05 (0.03 to 0.06) | 5.434 | **<0.001** | 0.02 (0.01 to 0.03) | 2.105 | **<0.001** | 0.03 ( 0.01 to 0.04) | 0.751 | **0.002** |
| aBMD-Spine (*g/cm^2^*) |  |  |  |  |  |  |  |  |  |
| *Baseline to 12-w* | 0.03 (0.01 to 0.05) | 3.448 | **<0.001** | 0.03 (0.01 to 0.05) | 3.333 | **<0.001** | 0.00 (-0.02 to 0.02) | 0.000 | 0.994 |
| *12-w to 24-w* | 0.01 (-0.01 to 0.02) | 1.149 | 0.762 | 0.01 (-0.01 to 0.02) | 1.111 | 0.418 | 0.00 (-0.02 to 0.01) | 0.000 | 0.746 |
| *Baseline to 24-w* | 0.04 (0.02 to 0.05) | 4.597 | **<0.001** | 0.04 (0.02 to 0.05) | 4.444 | **<0.001** | 0.00 (-0.02 to 0.01) | 0.000 | 0.739 |
| The results are presented as the mean difference for each group and the mean difference between the groups. Data were analysed using linear-mixed models.  Bold letters indicate significant differences.  ^Ψ^The percentage change (corresponding assessment less baseline values/baseline values × 100) was calculated.  *Abbreviations*: CI, confidence interval; BMI, body mass index; WC, waist circumference; ALMI, appendicular lean mass index; BMC, body mass content; TBLH, total body less head; aBMD, areal body mass density. | | | | | | | | | |

# **Table ESM10.** Within-group and between-group differences (Diactive-1 and usual care) in standardised outcomes (z-scores adjusted for sex, age, and ethnicity) from intention-to-treat models at baseline, 12 weeks, and 24 weeks

|  | Within-group differences (Diactive-1; *n*=30) | | | Within-group differences (Usual care; *n*=32) | | | Between-groups differences | | |
| --- | --- | --- | --- | --- | --- | --- | --- | --- | --- |
| Variables | Change  (95% CI) | Change %^Ψ^ | *p*  value | Change  (95% CI) | Change %^Ψ^ | *p*  value | Mean difference  (95% CI) | Hedges' *g* | *p*  value |
| Fat mass (*reference z*) |  |  |  |  |  |  |  |  |  |
| *Baseline to 12-w* | 0.02 (-0.09 to 0.13) | 4.651 | 0.942 | -0.04 (-0.15 to 0.06) | -6.896 | 0.592 | 0.06 (-0.07 to 0.19) | 0.234 | 0.365 |
| *12-w to 24-w* | -0.01 (-0.12 to 0.11) | -2.325 | 0.991 | -0.06 (-0.17 to 0.04) | -18.965 | 0.385 | 0.05 (-0.08 to 0.19) | 0.188 | 0.428 |
| *Baseline to 24-w* | 0.01 (-0.10 to 0.12) | 2.325 | 0.980 | -0.11 (-0.21 to 0.01) | -10.344 | 0.061 | 0.11 (-0.02 to 0.24) | 0.429 | 0.093 |
| Lean mass (*reference z*) |  |  |  |  |  |  |  |  |  |
| *Baseline to 12-w* | 0.25 (0.05 to 0.44) | 38.461 | **0.007** | 0.11 (-0.07 to 0.29) | 11.224 | 0.313 | 0.14 (-0.08 to 0.35) | 0.330 | 0.221 |
| *12-w to 24-w* | -0.19 (-0.38 to 0.01) | -29.230 | 0.076 | -0.17 (-0.35 to 0.01) | -17.346 | 0.078 | -0.02 (-0.24 to 0.20) | -0.046 | 0.877 |
| *Baseline to 24-w* | 0.06 (-0.13 to 0.26) | 9.230 | 0.740 | -0.06 (-0.24 to 0.12) | -6.122 | 0.737 | 0.12 (-0.10 to 0.34) | 0.276 | 0.296 |
| BMC-TBLH (*reference z*) |  |  |  |  |  |  |  |  |  |
| *Baseline to 12-w* | 0.02 (-0.15 to 0.19) | 1.923 | 0.961 | -0.13 (-0.29 to 0.03) | -19.117 | 0.157 | 0.15 (-0.05 to 0.35) | 0.380 | 0.146 |
| *12-w to 24-w* | -0.03 (-0.21 to 0.15) | -2.884 | 0.936 | 0.02 (-0.14 to 0.18) | 2.941 | 0.957 | -0.05 (-0.25 to 0.16) | -0.124 | 0.657 |
| *Baseline to 24-w* | -0.01 (-0.19 to 0.17) | -0.961 | 0.995 | -0.11 (-0.27 to 0.05) | -16.176 | 0.272 | 0.10 (-0.10 to 0.31) | 0.247 | 0.327 |
| aBMD-TBLH (*reference z*) |  |  |  |  |  |  |  |  |  |
| *Baseline to 12-w* | 0.04 (-0.15 to 0.24) | 5.714 | 0.860 | -0.05 (-0.23 to 0.14) | -14.285 | 0.827 | 0.09 (-0.13 to 0.31) | 0.207 | 0.435 |
| *12-w to 24-w* | -0.03 (-0.24 to 0.17) | -4.285 | 0.916 | 0.04 (-0.14 to 0.23) | 11.428 | 0.864 | -0.08 (-0.31 to 0.15) | -0.176 | 0.522 |
| *Baseline to 24-w* | 0.01 (-0.19 to 0.21) | 1.428 | 0.994 | -0.01 (-0.19 to 0.18) | -2.857 | 0.997 | 0.01 (-0.21 to 0.24) | 0.023 | 0.904 |
| The results are presented as the mean difference for each group and the mean difference between the groups. Data were analysed using linear-mixed models.  Bold letters indicate significant differences.  ^Ψ^The percentage change (corresponding assessment less baseline values/baseline values × 100) was calculated.  Age- and sex- handgrip strength z-scores are presented using the reference data from FitBack network. Age-, sex-, and race/ethnicity- body composition z-scores are presented using the reference data from BMD in Childhood Study.  *Abbreviations*: CI, confidence interval; BMC, body mass content; TBLH, total body less head; aBMD, areal body mass density. | | | | | | | | | |

# **Table ESM11.** Within-group and between-group differences (Diactive-1 and usual care) in standardised outcomes (z-scores adjusted for sex, age, and ethnicity) from per-protocol models at baseline, 12 weeks, and 24 weeks

|  | Within-group differences (Diactive-1; *n*=27) | | | Within-group differences (Usual care; *n*=31) | | | Between-groups differences | | |
| --- | --- | --- | --- | --- | --- | --- | --- | --- | --- |
| Variables | Change  (95% CI) | Change %^Ψ^ | *p*  value | Change  (95% CI) | Change %^Ψ^ | *p*  value | Mean difference  (95% CI) | Hedges' *g* | *p*  value |
| Fat mass (*reference z*) |  |  |  |  |  |  |  |  |  |
| *Baseline to 12-w* | 0.02 (-0.10 to 0.14) | 6.451 | 0.915 | -0.04 (-0.15 to 0.06) | -6.896 | 0.594 | 0.07 (-0.07 to 0.20) | 0.271 | 0.343 |
| *12-w to 24-w* | -0.01 (-0.12 to 0.11) | -3.225 | 0.992 | -0.06 (-0.17 to 0.04) | -10.344 | 0.389 | 0.05 (-0.08 to 0.19) | 0.191 | 0.430 |
| *Baseline to 24-w* | 0.01 (-0.10 to 0.13) | 3.225 | 0.957 | -0.11 (-0.21 to 0.00) | -18.965 | 0.063 | 0.12 (-0.01 to 0.25) | 0.464 | 0.084 |
| Lean mass (*reference z*) |  |  |  |  |  |  |  |  |  |
| *Baseline to 12-w* | 0.26 (0.05 to 0.45) | 38.235 | **0.007** | 0.11 (-0.06 to 0.29) | 10.891 | 0.294 | 0.15 (-0.07 to 0.36) | 0.352 | 0.201 |
| *12-w to 24-w* | -0.19 (-0.39 to 0.01) | -27.941 | 0.069 | -0.17 (-0.35 to 0.01) | -16.831 | 0.076 | -0.02 (-0.24 to 0.20) | -0.046 | 0.847 |
| *Baseline to 24-w* | 0.07 (-0.13 to 0.26) | 10.294 | 0.695 | -0.05 (-0.23 to 0.12) | -4.950 | 0.755 | 0.12 (-0.10 to 0.34) | 0.280 | 0.281 |
| BMC-TBLH (*reference z*) |  |  |  |  |  |  |  |  |  |
| *Baseline to 12-w* | 0.04 (-0.14 to 0.22) | 4.123 | 0.868 | -0.13 (-0.29 to 0.03) | -19.402 | 0.149 | 0.17 (-0.03 to 0.37) | 0.438 | 0.104 |
| *12-w to 24-w* | -0.03 (-0.21 to 0.15) | -3.092 | 0.899 | 0.02 (-0.14 to 0.18) | 2.985 | 0.954 | -0.05 (-0.26 to 0.15) | -0.127 | 0.604 |
| *Baseline to 24-w* | 0.00 (-0.17 to 0.18) | 0.000 | 0.998 | -0.11 (-0.27 to 0.05) | -16.417 | 0.263 | 0.11 (-0.09 to 0.31) | 0.282 | 0.272 |
| aBMD-TBLH (*reference z*) |  |  |  |  |  |  |  |  |  |
| *Baseline to 12-w* | 0.04 (-0.16 to 0.24) | 6.451 | 0.899 | -0.05 (-0.23 to 0.14) | -14.705 | 0.832 | 0.08 (-0.15 to 0.31) | 0.180 | 0.478 |
| *12-w to 24-w* | -0.03 (-0.24 to 0.18) | -4.838 | 0.941 | 0.04 (-0.14 to 0.23) | 11.764 | 0.864 | -0.07 (-0.30 to 0.16) | -0.156 | 0.555 |
| *Baseline to 24-w* | 0.01 (-0.19 to 0.21) | 1.612 | 0.994 | 0.00 (-0.19 to 0.18) | 0.000 | 0.998 | 0.01 (-0.22 to 0.24) | 0.022 | 0.911 |
| The results are presented as the mean difference for each group and the mean difference between the groups. Data were analysed using linear-mixed models.  Bold letters indicate significant differences.  ^Ψ^The percentage change (corresponding assessment less baseline values/baseline values × 100) was calculated.  Age- and sex- handgrip strength z-scores are presented using the reference data from FitBack network. Age-, sex-, and race/ethnicity- body composition z-scores are presented using the reference data from BMD in Childhood Study.  *Abbreviations*: CI, confidence interval; BMC, body mass content; TBLH, total body less head; aBMD, areal body mass density. | | | | | | | | | |

# **Table ESM12.** Within-group and between-group differences (Diactive-1 and usual care) resulting from linear mixed models at baseline, 12 weeks and 24 week, and adjusted for duration of diabetes since onset.

|  | Within-group differences (Diactive-1; *n*=30) | | | Within-group differences (Usual care; *n*=32) | | | Between-groups differences | | |
| --- | --- | --- | --- | --- | --- | --- | --- | --- | --- |
| Variables | Change  (95% CI) | Change %^Ψ^ | *p*  value | Change  (95% CI) | Change %^Ψ^ | *p*  value | Mean difference  (95% CI) | Hedges' *g* | *p*  value |
| **Anthropometric** | | | | | | | | | |
| BMI (*kg/m^2^*) |  |  |  |  |  |  |  |  |  |
| *Baseline to 24-w* | 0.38 (-0.08 to 0.83) | 1.794 | 0.104 | 0.15 (-0.27 to 0.57) | 0.722 | 0.114 | 0.23 (-0.33 to 0.79) | 0.203 | 0.426 |
| WC (*cm*) |  |  |  |  |  |  |  |  |  |
| *Baseline to 12-w* | 0.50 (-1.29 to 2.28) | 0.714 | 0.583 | 0.36 (-1.30 to 2.01) | 0.670 | 0.349 | 0.14 (-2.23 to 2.51) | 0.030 | 0.906 |
| WHtR (score) |  |  |  |  |  |  |  |  |  |
| *Baseline to 24-w* | -0.23 (-1.32 to 0.85) | -0.527 | 0.770 | -0.03 (-1.03 to 0.97) | -0.071 | 0.912 | -0.20 (-1.65 to 1.25) | -0.070 | 0.782 |
| **Body composition (DXA)** | | | | | | | | | |
| Fat mass (*kg*) |  |  |  |  |  |  |  |  |  |
| *Baseline to 12-w* | 0.17 (-0.63 to 0.96) | 1.029 | 0.873 | 0.07 (-0.69 to 0.82) | 0.445 | 0.975 | 0.10 (-0.76 to 0.96) | 0.058 | 0.820 |
| *12-w to 24-w* | 0.01 (-0.81 to 0.78) | -0.060 | 0.999 | -0.31 (-1.04 to 0.41) | -1.589 | 0.565 | 0.30 (-0.59 to 1.18) | 0.169 | 0.507 |
| *Baseline to 24-w* | 0.15 (-0.7 to 1.03) | 0.907 | 0.910 | -0.25 (-1.07 to 0.58) | -1.970 | 0.762 | 0.40 (-0.48 to 1.28) | 0.227 | 0.373 |
| Arm fat mass *(kg)* |  |  |  |  |  |  |  |  |  |
| *Baseline to 12-w* | -0.01 (-0.116 to 0.099) | -0.540 | 0.980 | -0.01 (-0.111 to 0.093) | -0.543 | 0.976 | 0.00 (-0.119 to 0.120) | 0.000 | 0.995 |
| *12-w to 24-w* | 0.07 (-0.044 to 0.176) | 3.783 | 0.332 | -0.03 (-0.134 to 0.066) | -1.630 | 0.702 | 0.10 (-0.023 to 0.223) | 0.410 | 0.111 |
| *Baseline to 24-w* | 0.06 (-0.057 to 0.172) | 3.243 | 0.463 | -0.04 (-0.150 to 0.064) | -2.173 | 0.612 | 0.10 (-0.022 to 0.222) | 0.414 | 0.107 |
| Leg fat mass *(kg)* |  |  |  |  |  |  |  |  |  |
| *Baseline to 12-w* | 0.02 (-0.276 to 0.313) | 0.278 | 0.988 | -0.05 (-0.334 to 0.228) | -0.713 | 0.897 | 0.07 (-0.243 to 0.385) | 0.114 | 0.654 |
| *12-w to 24-w* | 0.01 (-0.284 to 0.301) | 0.139 | 0.997 | -0.03 (-0.294 to 0.238) | -0.427 | 0.965 | 0.04 (-0.287 to 0.360) | 0.057 | 0.822 |
| *Baseline to 24-w* | 0.03 (-0.301 to 0.354) | 0.418 | 0.979 | -0.08 (-0.390 to 0.229) | -1.141 | 0.810 | 0.11 (-0.213 to 0.428) | 0.169 | 0.507 |
| Trunk fat mass *(kg)* |  |  |  |  |  |  |  |  |  |
| *Baseline to 12-w* | 0.16 (-0.310 to 0.622) | 2.398 | 0.707 | 0.13 (-0.312 to 0.572) | 2.148 | 0.764 | 0.03 (-0.486 to 0.537) | 0.025 | 0.921 |
| *12-w to 24-w* | -0.09 (-0.566 to 0.379) | -1.349 | 0.886 | -0.25 (-0.675 to 0.183) | -4.132 | 0.365 | 0.15 (-0.374 to 0.679) | 0.146 | 0.566 |
| *Baseline to 24-w* | 0.06 (-0.440 to 0.566) | 0.899 | 0.953 | -0.12 (-0.588 to 0.356) | -1.983 | 0.830 | 0.18 (-0.344 to 0.701) | 0.172 | 0.500 |
| Fat mass (*%*) |  |  |  |  |  |  |  |  |  |
| *Baseline to 12-w* | -0.76 (-1.68 to 0.16) | -2.652 | 0.131 | -0.99 (-1.86 to -0.10) | -3.525 | **0.024** | 0.23 (-0.78 to 1.24) | 0.113 | 0.658 |
| *12-w to 24-w* | 0.14 (-0.79 to 1.08) | 0.488 | 0.315 | -0.11 (-0.96 to 0.74) | -0.391 | 0.951 | 0.25 (-0.79 to 1.29) | 0.120 | 0.637 |
| *Baseline to 24-w* | -0.62 (-1.62 to 0.38) | -2.164 | 0.932 | -1.09 (-2.03 to -0.15) | -3.881 | **0.018** | 0.48 (-0.56 to 1.51) | 0.232 | 0.364 |
| Visceral fat (*g*) |  |  |  |  |  |  |  |  |  |
| *Baseline to 12-w* | 17.22 (-16.704 to 51.144) | 12.120 | 0.452 | 26.76 ( -3.838 to 57.354) | 19.138 | 0.099 | -9.54 (-47.074 to 27.998) | -0.128 | 0.615 |
| *12-w to 24-w* | -17.80 (-51.870 to 16.264) | -12.529 | 0.431 | -19.92 (-50.49 to 10.65) | -14.246 | 0.272 | 2.12 (-35.942 to 40.178) | 0.028 | 0.912 |
| *Baseline to 24-w* | -0.58 (-33.555 to 32.390) | -0.408 | 0.999 | 6.84 (-23.659 to 37.333) | 4.892 | 0.855 | -7.42 (-43.632 to 28.792) | -0.103 | 0.685 |
| Subcutaneous fat (*g*) |  |  |  |  |  |  |  |  |  |
| *Baseline to 12-w* | -110.74 (-218.94 to -2.53) | -13.724 | **0.043** | -16.19 (-117.01 to 84.62) | -2.459 | 0.923 | -94.54 (-215.07 to 25.98) | -0.397 | 0.122 |
| *12-w to 24-w* | 42.62 (-65.46 to 150.69) | 5.282 | 0.618 | -14.32 (-114.35 to 85.70) | -2.175 | 0.938 | 56.94 (-65.24 to 179.12) | 0.235 | 0.357 |
| *Baseline to 24-w* | -68.12 (-177.60 to 41.36) | -8.442 | 0.306 | -30.52 (-131.99 to 70.95) | -4.636 | 0.756 | -37.60 (-155.85 to 80.64) | -0.160 | 0.529 |
| Lean mass (*kg*) |  |  |  |  |  |  |  |  |  |
| *Baseline to 12-w* | 2.12 ( 1.33 to 2.89) | 5.723 | **<0.001** | 1.59 ( 0.83 to 2.34) | 4.132 | **<0.001** | 0.53 (-0.23 to 1.29) | 0.347 | 0.175 |
| *12-w to 24-w* | -0.19 (-0.92 to 0.54) | -0.512 | 0.816 | -0.54 (-1.20 to 0.12) | -1.403 | 0.135 | 0.35 (-0.43 to 1.14) | 0.226 | 0.376 |
| *Baseline to 24-w* | 1.93 ( 1.00 to 2.85) | 5.210 | **<0.001** | 1.04 ( 0.158 to 1.93) | 2.702 | **0.016** | 0.88 ( 0.09 to 1.66) | 0.574 | **0.027** |
| Arm lean mass *(kg)* |  |  |  |  |  |  |  |  |  |
| *Baseline to 12-w* | 0.09 (-0.111 to 0.281) | 2.349 | 0.559 | -0.02 (-0.208 to 0.164) | -0.478 | 0.957 | 0.11 (-0.105 to 0.319) | 0.254 | 0.320 |
| *12-w to 24-w* | 0.21 ( 0.012 to 0.405) | 5.483 | **0.035** | 0.20 ( 0.019 to 0.377) | 4.784 | **0.026** | 0.01 (-0.208 to 0.229) | 0.025 | 0.922 |
| *Baseline to 24-w* | 0.29 ( 0.080 to 0.507) | 7.571 | **0.004** | 0.18 (-0.025 to 0.377) | 4.306 | 0.099 | 0.12 (-0.099 to 0.335) | 0.274 | 0.284 |
| Leg lean mass *(kg)* |  |  |  |  |  |  |  |  |  |
| *Baseline to 12-w* | 0.75 ( 0.397 to 1.105) | 5.787 | **<0.001** | 0.62 ( 0.280 to 0.961) | 4.538 | **<0.001** | 0.13 (-0.236 to 0.496) | 0.179 | 0.482 |
| *12-w to 24-w* | -0.12 (-0.468 to 0.220) | -0.925 | 0.670 | -0.27 (-0.587 to 0.039) | -1.976 | 0.099 | 0.15 (-0.227 to 0.527) | 0.201 | 0.431 |
| *Baseline to 24-w* | 0.63 ( 0.222 to 1.033) | 4.861 | **0.001** | 0.35 (-0.039 to 0.733) | 2.562 | 0.087 | 0.28 (-0.094 to 0.654) | 0.379 | 0.140 |
| Trunk lean mass *(kg)* |  |  |  |  |  |  |  |  |  |
| *Baseline to 12-w* | 1.27 ( 0.790 to 1.741) | 7.388 | **<0.001** | 0.98 ( 0.529 to 1.440) | 5.571 | **<0.001** | 0.28 (-0.220 to 0.781) | 0.282 | 0.269 |
| *12-w to 24-w* | -0.28 (-0.747 to 0.189) | -1.628 | 0.336 | -0.46 (-0.889 to -0.038) | -2.615 | **0.029** | 0.18 (-0.330 to 0.700) | 0.181 | 0.478 |
| *Baseline to 24-w* | 0.99 ( 0.451 to 1.522) | 5.759 | **<0.001** | 0.52 ( 0.013 to 1.029) | 2.956 | **0.042** | 0.46 (-0.046 to 0.976) | 0.460 | **0.074** |
| BMC (*g*) |  |  |  |  |  |  |  |  |  |
| *Baseline to 12-w* | 68.59 (39.64 to 97.52) | 3.418 | **<0.001** | 41.34 (12.77 to 69.89) | 1.965 | **0.002** | 27.25 (1.53 to 52.96) | 0.537 | **0.038** |
| *12-w to 24-w* | 43.97 (18.45 to 69.47) | 2.191 | **<0.001** | 37.60 (14.42 to 60.78) | 1.787 | **0.001** | 6.36 (-20.11 to 32.83) | 0.121 | 0.634 |
| *Baseline to 24-w* | 112.55 (75.95 to 149.14) | 5.609 | **<0.001** | 78.94 (43.35 to 114.52) | 3.753 | **<0.001** | 33.61 (7.35 to 59.87) | 0.656 | **0.012** |
| BMC-TBLH (*g*) |  |  |  |  |  |  |  |  |  |
| *Baseline to 12-w* | 64.70 (37.06 to 92.33) | 4.078 | **<0.001** | 37.74 (10.55 to 64.92) | 2.249 | **<0.001** | 26.96 (1.89 to 52.03) | 0.546 | **0.035** |
| *12-w to 24-w* | 38.45 (13.77 to 63.11) | 2.423 | **<0.001** | 32.97 (10.55 to 55.38) | 1.965 | **0.001** | 5.48 (-20.33 to 31.28) | 0.107 | 0.674 |
| *Baseline to 24-w* | 103.15 (68.58 to 137.71) | 6.502 | **<0.001** | 70.71 (37.16 to 104.24) | 4.214 | **<0.001** | 32.44 (6.83 to 58.04) | 0.648 | **0.013** |
| BMC-Arms (*g*) |  |  |  |  |  |  |  |  |  |
| *Baseline to 12-w* | 4.45 (-4.412 to 13.306) | 1.812 | 0.461 | 0.23 (-8.227 to 8.678) | 0.086 | 0.997 | 4.22 (-5.269 to 13.711) | 0.224 | 0.379 |
| *12-w to 24-w* | 14.89 ( 6.062 to 23.721) | 6.063 | **<0.001** | 10.88 ( 2.854 to 18.907) | 4.106 | **0.004** | 4.01 (-5.761 to 13.781) | 0.207 | 0.417 |
| *Baseline to 24-w* | 19.34 ( 9.533 to 29.143) | 7.875 | **<0.001** | 11.11 ( 1.848 to 20.365) | 4.193 | **0.014** | 8.23 (-1.463 to 17.926) | 0.429 | 0.095 |
| BMC-Legs (*g*) |  |  |  |  |  |  |  |  |  |
| *Baseline to 12-w* | 24.35 (11.869 to 36.839) | 3.145 | **<0.001** | 10.48 (-1.818 to 22.784) | 1.286 | 0.111 | 13.87 ( 2.650 to 25.093) | 0.634 | **0.015** |
| *12-w to 24-w* | 16.98 ( 5.896 to 28.059) | 2.193 | **0.001** | 21.25 (11.185 to 31.324) | 2.608 | **<0.001** | -4.28 (-15.830 to 7.275) | -0.187 | 0.464 |
| *Baseline to 24-w* | 41.33 (25.635 to 57.028) | 5.339 | **<0.001** | 31.74 (16.492 to 46.983) | 3.896 | **<0.001** | 9.59 ( -1.866 to 21.053) | 0.424 | 0.099 |
| BMC-Pelvis (*g*) |  |  |  |  |  |  |  |  |  |
| *Baseline to 12-w* | 19.31 ( 10.480 to 28.148) | 7.729 | **<0.001** | 14.22 ( 5.759 to 22.681) | 5.442 | **<0.001** | 5.09 (-4.207 to 14.395) | 0.276 | 0.280 |
| *12-w to 24-w* | 4.25 ( -4.450 to 12.948) | 1.701 | 0.479 | -3.03 (-10.933 to 4.881) | -1.159 | 0.636 | 7.28 (-2.300 to 16.851) | 0.384 | 0.135 |
| *Baseline to 24-w* | 23.56 ( 13.622 to 33.505) | 9.431 | **<0.001** | 11.19 ( 1.767 to 20.621) | 4.283 | **0.015** | 12.37 ( 2.869 to 21.870) | 0.665 | **0.011** |
| BMC-Spine (*g*) |  |  |  |  |  |  |  |  |  |
| *Baseline to 12-w* | 6.76 ( 1.872 to 11.642) | 5.228 | **0.003** | 5.19 ( 0.517 to 9.856) | 3.752 | **0.025** | 1.57 (-3.619 to 6.760) | 0.153 | 0.549 |
| *12-w to 24-w* | 3.06 (-1.782 to 7.898) | 2.366 | 0.294 | 1.68 (-2.716 to 6.084) | 1.214 | 0.636 | 1.37 (-3.969 to 6.717) | 0.129 | 0.611 |
| *Baseline to 24-w* | 9.81 ( 4.364 to 15.265) | 7.588 | **<0.001** | 6.87 ( 1.713 to 12.028) | 4.967 | **0.005** | 2.94 (-2.357 to 8.245) | 0.280 | 0.273 |
| aBMD (*g/cm^2^*) |  |  |  |  |  |  |  |  |  |
| *Baseline to 12-w* | 0.02 ( 0.01 to 0.01) | 1.923 | **0.010** | 0.01 ( 0.01 to 0.02) | 0.943 | **0.019** | 0.00 (-0.01 to 0.01) | 0.000 | 0.785 |
| *12-w to 24-w* | 0.01 (-0.01 to 0.02) | 0.961 | 0.442 | 0.01 (-0.01 to 0.02) | 0.943 | 0.151 | 0.00 (-0.01 to 0.01) | 0.000 | 0.717 |
| *Baseline to 24-w* | 0.02 ( 0.01 to 0.03) | 1.923 | **<0.001** | 0.02 ( 0.01 to 0.03) | 1.886 | **0.002** | 0.00 (-0.01 to 0.01) | 0.000 | 0.921 |
| The results are presented as the mean difference for each group and the mean difference between the groups. Data was analysed using linear-mixed models.  Bold letters indicate significant differences.  ^Ψ^The percentage change (corresponding assessment less baseline values/baseline values × 100) was calculated.  *Abbreviations*: CI, confidence interval; BMI, body mass index; WC, waist circumference; BMC, body mass content; aBMD, areal body mass density. | | | | | | | | | |

# **Table ESM13.** Interaction of sex and maturation stage on both the effects within the Diactive-1 exercise group and the effects of the Diactive-1 exercise group versus the usual care group at 12 and 24 weeks using the intention-to-treat approach.

|  | **Within Diactive-1 exercise group** | | | | **Diactive-1 *vs* usual care** | | | |
| --- | --- | --- | --- | --- | --- | --- | --- | --- |
| **Body mass index** | Baseline to 12-w | | Baseline to 24-w | | At 12-w | | At 24-w | |
|  | Change (95%CI) | *p* | Change (95%CI) | *p* | MD (95%CI) | *p* | MD (95%CI) | *p* |
| *Sex* |  |  |  |  |  |  |  |  |
| Boys |  |  | 0.39 (-0.21 to 0.99) | 0.506 |  |  | 0.13 (-0.67 to 0.93) | 0.765 |
| Girls |  |  | 0.68 (0.07 to 1.28) |  |  |  | 0.31 (-0.53 to 1.14) |  |
| *Maturation stage* |  |  |  |  |  |  |  |  |
| Prepuberal |  |  | -0.35 (-1.89 to 1.19) | 0.872 |  |  | -0.20 (-2.87 to 2.46) | 0.858 |
| Peripuberal |  |  | 0.83 (0.25 to 1.41) |  |  |  | 0.42 (-0.48 to 1.32) |  |
| Postpuberal |  |  | 0.23 (-0.50 to 0.97) |  |  |  | -0.03 (-0.97 to 0.90) |  |
| **Waist circumference** | Baseline to 12-w | | Baseline to 24-w | | At 12-w | | At 24-w | |
|  | Change (95%CI) | *p* | Change (95%CI) | *p* | MD (95%CI) | *p* | MD (95%CI) | *p* |
| *Sex* |  |  |  |  |  |  |  |  |
| Boys |  |  | 1.34 (-1.07 to 3.74) | 0.605 |  |  | 2.18 (-1.02 to 5.38) | 0.062 |
| Girls |  |  | 0.45 (-1.96 to 2.86) |  |  |  | -2.23 (-5.57 to 1.12) |  |
| *Maturation stage* |  |  |  |  |  |  |  |  |
| Prepuberal |  |  | -2.82 (-9.27 to 3.62) | 0.898 |  |  | -0.92 (-12.04 to 10.20) | 0.557 |
| Peripuberal |  |  | 1.43 (-1.03 to 3.90) |  |  |  | 0.06 (-3.72 to 3.83) |  |
| Postpuberal |  |  | 0.22 (-2.89 to 3.33) |  |  |  | 0.44 (-3.51 to 4.39) |  |
| **WHtR** | Baseline to 12-w | | Baseline to 24-w | | At 12-w | | At 24-w | |
|  | Change (95%CI) | *p* | Change (95%CI) | *p* | MD (95%CI) | *p* | MD (95%CI) | *p* |
| *Sex* |  |  |  |  |  |  |  |  |
| Boys |  |  | -0.00 (-0.02 to 0.01) | 0.998 |  |  | 0.01 (-0.01 to 0.03) | 0.102 |
| Girls |  |  | -0.00 (-0.02 to 0.01) |  |  |  | -0.01 (-0.04 to 0.01) |  |
| *Maturation stage* |  |  |  |  |  |  |  |  |
| Prepuberal |  |  | -0.01 (-0.03 to 0.02) | 0.532 |  |  | 0.01 (-0.03 to 0.05) | 0.516 |
| Peripuberal |  |  | -0.00 (-0.02 to 0.02) |  |  |  | -0.01 (-0.04 to 0.02) |  |
| Postpuberal |  |  | 0.00 (-0.02 to 0.02) |  |  |  | 0.00 (-0.02 to 0.02) |  |
| **Fat mass** | Baseline to 12-w | | Baseline to 24-w | | At 12-w | | At 24-w | |
|  | Change (95%CI) | *p* | Change (95%CI) | *p* | MD (95%CI) | *p* | MD (95%CI) | *p* |
| *Sex* |  |  |  |  |  |  |  |  |
| Boys | 0.10 (-0.78 to 0.98) | 0.288 | 0.09 (-0.82 to 1.00) | 0.147 | -0.56 (-1.76 to 0.63) | 0.116 | -0.12 (-1.33 to 1.10) | 0.241 |
| Girls | 0.79 (-0.13 to 1.71) |  | 1.06 (0.11 to 2.01) |  | 0.84 (-0.44 to 2.11) |  | 0.95 (-0.37 to 2.26) |  |
| *Maturation stage* |  |  |  |  |  |  |  |  |
| Prepuberal | 0.61 (-1.36 to 2.57) | 0.551 | 0.15 (-2.15 to 2.45) | 0.402 | 0.72 (-0.55 to 1.98) | 0.172 | 0.84 (-0.55 to 2.23) | 0.387 |
| Peripuberal | 0.56 (-0.31 to 1.43) |  | 0.92 (0.02 to 1.81) |  | -0.72 (-2.16 to 0.72) |  | -0.48 (-1.97 to 1.00) |  |
| Postpuberal | 0.11 (-1.02 to 1.25) |  | -0.11 (-1.30 to 1.08) |  | 1.17 (-2.74 to 5.08) |  | 1.02 (-3.07 to 5.11) |  |
| **Arm fat mass** | Baseline to 12-w | | Baseline to 24-w | | At 12-w | | At 24-w | |
|  | Change (95%CI) | *p* | Change (95%CI) | *p* | MD (95%CI) | *p* | MD (95%CI) | *p* |
| *Sex* |  |  |  |  |  |  |  |  |
| Boys | 0.05 (-0.07 to 0.17) | 0.487 | 0.06 (-0.07 to 0.18) | 0.392 | 0.05 (-0.11 to 0.22) | 0.327 | 0.10 (-0.06 to 0.27) | 0.891 |
| Girls | -0.02 (-0.14 to 0.11) |  | 0.14 (0.00 to 0.27) |  | -0.06 (-0.24 to 0.11) |  | 0.09 (-0.10 to 0.27) |  |
| *Maturation stage* |  |  |  |  |  |  |  |  |
| Prepuberal | 0.04 (-0.23 to 0.31) | 0.722 | -0.07 (-0.38 to 0.25) | 0.493 | 0.03 (-0.51 to 0.56) | 0.819 | 0.02 (-0.54 to 0.58) | 0.829 |
| Peripuberal | 0.02 (-0.10 to 0.14) |  | 0.17 (0.05 to 0.29) |  | -0.02 (-0.19 to 0.16) |  | 0.15 (-0.04 to 0.34) |  |
| Postpuberal | -0.01 (-0.16 to 0.15) |  | -0.01 (-0.17 to 0.16) |  | 0.01 (-0.19 to 0.20) |  | 0.02 (-0.18 to 0.22) |  |
| **Leg fat mass** | Baseline to 12-w | | Baseline to 24-w | | At 12-w | | At 24-w | |
|  | Change (95%CI) | *p* | Change (95%CI) | *p* | MD (95%CI) | *p* | MD (95%CI) | *p* |
| *Sex* |  |  |  |  |  |  |  |  |
| Boys | -0.01 (-0.33 to 0.32) | 0.208 | 0.05 (-0.28 to 0.38) | 0.171 | -0.15 (-0.59 to 0.28) | 0.153 | -0.04 (-0.48 to 0.41) | 0.375 |
| Girls | 0.29 (-0.04 to 0.63) |  | 0.38 (0.04 to 0.73) |  | 0.31 (-0.15 to 0.78) |  | 0.26 (-0.22 to 0.74) |  |
| *Maturation stage* |  |  |  |  |  |  |  |  |
| Prepuberal | 0.05 (-0.67 to 0.78) | 0.758 | -0.02 (-0.87 to 0.82) | 0.595 | 0.40 (-1.03 to 1.84) | 0.356 | 0.44 (-1.06 to 1.94) | 0.548 |
| Peripuberal | 0.20 (-0.12 to 0.52) |  | 0.33 (0.00 to 0.66) |  | 0.25 (-0.21 to 0.72) |  | 0.23 (-0.28 to 0.74) |  |
| Postpuberal | 0.04 (-0.38 to 0.45) |  | 0.01 (-0.43 to 0.44) |  | -0.15 (-0.68 to 0.38) |  | -0.13 (-0.67 to 0.41) |  |
| **Trunk fat mass** | Baseline to 12-w | | Baseline to 24-w | | At 12-w | | At 24-w | |
|  | Change (95%CI) | *p* | Change (95%CI) | *p* | MD (95%CI) | *p* | MD (95%CI) | *p* |
| *Sex* |  |  |  |  |  |  |  |  |
| Boys | 0.05 (-0.46 to 0.57) | 0.234 | -0.05 (-0.58 to 0.49) | 0.135 | -0.47 (-1.17 to 0.23) | **0.043** | -0.21 (-0.93 to 0.50) | 0.120 |
| Girls | 0.50 (-0.04 to 1.04) |  | 0.54 (-0.02 to 1.09) |  | 0.59 (-0.16 to 1.34) |  | 0.61 (-0.16 to 1.39) |  |
| *Maturation stage* |  |  |  |  |  |  |  |  |
| Prepuberal | 0.50 (-0.65 to 1.65) | 0.459 | 0.21 (-1.13 to 1.56) | 0.377 | 0.78 (-1.51 to 3.07) | 0.069 | 0.58 (-1.81 to 2.97) | 0.290 |
| Peripuberal | 0.33 (-0.18 to 0.84) |  | 0.39 (-0.14 to 0.91) |  | 0.47 (-0.27 to 1.22) |  | 0.45 (-0.36 to 1.26) |  |
| Postpuberal | 0.08 (-0.59 to 0.74) |  | -0.11 (-0.80 to 0.59) |  | -0.58 (-1.42 to 0.27) |  | -0.38 (-1.24 to 0.49) |  |
| **Fat mass (%)** | Baseline to 12-w | | Baseline to 24-w | | At 12-w | | At 24-w | |
|  | Change (95%CI) | *p* | Change (95%CI) | *p* | MD (95%CI) | *p* | MD (95%CI) | *p* |
| *Sex* |  |  |  |  |  |  |  |  |
| Boys | -0.88 (-1.91 to 0.14) | 0.342 | -0.92 (-1.97 to 0.14) | 0.103 | -0.62 (-2.01 to 0.77) | 0.078 | -0.20 (-1.61 to 1.21) | 0.195 |
| Girls | -0.17 (-1.24 to 0.90) |  | 0.35 (-0.76 to 1.45) |  | 1.20 (-0.28 to 2.69) |  | 1.17 (-0.36 to 2.70) |  |
| *Maturation stage* |  |  |  |  |  |  |  |  |
| Prepuberal | -0.08 (-2.39 to 2.22) | 0.733 | -0.58 (-3.27 to 2.12) | 0.953 | 2.16 (-2.42 to 6.73) | 0.095 | 2.45 (-2.34 to 7.23) | 0.303 |
| Peripuberal | -0.60 (-1.62 to 0.42) |  | -0.32 (-1.36 to 0.73) |  | 0.82 (-0.66 to 2.31) |  | 0.79 (-0.84 to 2.41) |  |
| Postpuberal | -0.65 (-1.97 to 0.68) |  | -0.33 (-1.71 to 1.06) |  | -0.67 (-2.36 to 1.02) |  | -0.13 (-1.86 to 1.61) |  |
| **Visceral fat** | Baseline to 12-w | | Baseline to 24-w | | At 12-w | | At 24-w | |
|  | Change (95%CI) | *p* | Change (95%CI) | *p* | MD (95%CI) | *p* | MD (95%CI) | *p* |
| *Sex* |  |  |  |  |  |  |  |  |
| Boys | 12.22 (-25.10 to 49.54) | 0.585 | 16.51 (-20.89 to 53.91) | 0.268 | -25.69 (-76.02 to 24.64) | 0.339 | -7.20 (-57.08 to 42.69) | 0.947 |
| Girls | 27.64 (-14.01 to 69.29) |  | -13.29 (-50.97 to 24.40) |  | 10.67 (-45.09 to 66.44) |  | -4.78 (-56.94 to 47.37) |  |
| *Maturation stage* |  |  |  |  |  |  |  |  |
| Prepuberal | 63.33 (-15.95 to 142.62) | 0.359 | 49.82 (-42.60 to 142.25) | 0.405 | 113.88 (-41.81 to 269.57) | 0.167 | 107.37 (-55.41 to 270.14) | **0.046** |
| Peripuberal | 14.70 (-25.04 to 54.44) |  | -4.04 (-40.18 to 32.10) |  | -6.39 (-61.04 to 48.27) |  | 21.75 (-32.81 to 76.31) |  |
| Postpuberal | 10.89 (-34.89 to 56.66) |  | -3.22 (-49.00 to 42.55) |  | -25.23 (-84.37 to 33.92) |  | -26.49 (-84.12 to 31.13) |  |
| **Subcutaneous fat** | Baseline to 12-w | | Baseline to 24-w | | At 12-w | | At 24-w | |
|  | Change (95%CI) | *p* | Change (95%CI) | *p* | MD (95%CI) | *p* | MD (95%CI) | *p* |
| *Sex* |  |  |  |  |  |  |  |  |
| Boys | 28.71 (-90.45 to147.87) | **0.004** | -22.33 (-141.65 to 96.99) | 0.594 | -20.51 (-181.16 to 140.13) | 0.211 | -31.21 (-190.36 to 127.94) | 0.882 |
| Girls | -226.74 (-350.69 to -102.78) |  | -67.95 (-188.24 to 52.34) |  | -169.67 (-341.06 to 1.72) |  | -48.56 (-215.02 to 117.89) |  |
| *Maturation stage* |  |  |  |  |  |  |  |  |
| Prepuberal | 9.67 (-247.31 to 266.64) | 0.431 | -35.77 (-335.97 to 264.44) | 0.771 | 121.43 (-386.63 to 629.50) | 0.424 | 79.00 (-452.24 to 610.24) | 0.335 |
| Peripuberal | -186.18 (-307.18 to -65.18) |  | -44.27 (-161.71 to 73.16) |  | -98.10 (-269.77 to 73.58) |  | 7.87 (-169.50 to 185.23) |  |
| Postpuberal | 5.11 (-143.25 to 153.48) |  | -58.22 (-206.59 to 90.14) |  | 7.87 (-169.50 to 185.23) |  | -114.56 (-301.48 to 72.35) |  |
| **Lean mass** | Baseline to 12-w | | Baseline to 24-w | | At 12-w | | At 24-w | |
|  | Change (95%CI) | *p* | Change (95%CI) | *p* | MD (95%CI) | *p* | MD (95%CI) | *p* |
| *Sex* |  |  |  |  |  |  |  |  |
| Boys | 2.59 (1.81 to 3.36) | 0.321 | 2.79 (1.99 to 3.59) | 0.055 | 0.93 (-0.13 to 1.98) | 0.256 | 1.46 (0.39 to 2.52) | 0.133 |
| Girls | 2.02 (1.21 to 2.83) |  | 1.66 (0.82 to 2.50) |  | 0.04 (-1.09 to 1.17) |  | 0.25 (-0.91 to 1.41) |  |
| *Maturation stage* |  |  |  |  |  |  |  |  |
| Prepuberal | 1.73 (0.08 to 3.38) | 0.918 | 1.36 (-0.57 to 3.30) | 0.140 | 0.33 (-2.96 to 3.63) | 0.427 | -0.27 (-3.72 to 3.17) | 0.680 |
| Peripuberal | 2.50 (1.77 to 3.24) |  | 3.00 (2.25 to 3.75) |  | 0.46 (-0.60 to 1.53) |  | 1.01 (-0.16 to 2.18) |  |
| Postpuberal | 2.18 (1.23 to 3.13) |  | 1.18 (0.18 to 2.17) |  | 0.69 (-0.52 to 1.90) |  | 0.34 (-0.91 to 1.58) |  |
| **Arm lean mass** | Baseline to 12-w | | Baseline to 24-w | | At 12-w | | At 24-w | |
|  | Change (95%CI) | *p* | Change (95%CI) | *p* | MD (95%CI) | *p* | MD (95%CI) | *p* |
| *Sex* |  |  |  |  |  |  |  |  |
| Boys | 0.24 (0.03 to 0.45) | 0.065 | 0.49 (0.27 to 0.70) | **0.034** | 0.31 (0.03 to 0.60) | **0.036** | 0.27 (-0.02 to 0.56) | 0.156 |
| Girls | -0.05 (-0.27 to 0.17) |  | 0.15 (-0.08 to 0.37) |  | -0.13 (-0.44 to 0.17) |  | -0.04 (-0.36 to 0.27) |  |
| *Maturation stage* |  |  |  |  |  |  |  |  |
| Prepuberal | 0.09 (-0.38 to 0.56) | 0.715 | -0.04 (-0.59 to 0.50) | 0.737 | -0.03 (-0.97 to 0.90) | 0.372 | -0.16 (-1.14 to 0.81) | 0.663 |
| Peripuberal | 0.14 (-0.07 to 0.35) |  | 0.47 (0.26 to 0.69) |  | 0.07 (-0.24 to 0.37) |  | 0.38 (0.04 to 0.71) |  |
| Postpuberal | 0.05 (-0.23 to 0.32) |  | 0.16 (-0.12 to 0.44) |  | 0.17 (-0.18 to 0.51) |  | -0.04 (-0.40 to 0.31) |  |
| **Leg lean mass** | Baseline to 12-w | | Baseline to 24-w | | At 12-w | | At 24-w | |
|  | Change (95%CI) | *p* | Change (95%CI) | *p* | MD (95%CI) | *p* | MD (95%CI) | *p* |
| *Sex* |  |  |  |  |  |  |  |  |
| Boys | 0.98 (0.60 to 1.35) | 0.299 | 0.89 (0.51 to 1.27) | 0.347 | 0.31 (-0.20 to 0.81) | 0.307 | 0.45 (-0.06 to 0.97) | 0.327 |
| Girls | 0.69 (0.30 to 1.08) |  | 0.63 (0.22 to 1.03) |  | -0.08 (-0.62 to 0.46) |  | 0.08 (-0.48 to 0.64) |  |
| *Maturation stage* |  |  |  |  |  |  |  |  |
| Prepuberal | 0.53 (-0.28 to 1.34) | 0.764 | 0.58 (-0.37 to 1.54) | 0.242 | -0.00 (-1.62 to 1.62) | 0.638 | -0.10 (-1.80 to 1.59) | 0.798 |
| Peripuberal | 0.91 (0.55 to 1.27) |  | 1.00 (0.63 to 1.37) |  | 0.19 (-0.33 to 0.72) |  | 0.34 (-0.24 to 0.92) |  |
| Postpuberal | 0.81 (0.34 to 1.28) |  | 0.38 (-0.11 to 0.87) |  | 0.15 (-0.45 to 0.74) |  | 0.06 (-0.56 to 0.67) |  |
| **Trunk lean mass** | Baseline to 12-w | | Baseline to 24-w | | At 12-w | | At 24-w | |
|  | Change (95%CI) | *p* | Change (95%CI) | *p* | MD (95%CI) | *p* | MD (95%CI) | *p* |
| *Sex* |  |  |  |  |  |  |  |  |
| Boys | 1.32 (0.81 to 1.83) | 0.889 | 1.33 (0.81 to 1.86) | 0.245 | 0.26 (-0.43 to 0.95) | 0.952 | 0.64 (-0.06 to 1.34) | 0.499 |
| Girls | 1.37 (0.84 to 1.91) |  | 0.89 (0.34 to 1.44) |  | 0.29 (-0.45 to 1.03) |  | 0.29 (-0.48 to 1.05) |  |
| *Maturation stage* |  |  |  |  |  |  |  |  |
| Prepuberal | 1.06 (-0.05 to 2.17) | 0.891 | 1.06 (-0.05 to 2.17) | 0.371 | 0.66 (-1.55 to 2.87) | 0.727 | 0.16 (-2.15 to 2.47) | 0.460 |
| Peripuberal | 1.42 (0.93 to 1.91) |  | 1.43 (0.93 to 1.94) |  | 0.23 (-0.49 to 0.94) |  | 0.32 (-0.47 to 1.10) |  |
| Postpuberal | 1.30 (0.66 to 1.94) |  | 0.64 (-0.03 to 1.31) |  | 0.39 (-0.42 to 1.21) |  | 0.41 (-0.43 to 1.24) |  |
| **BMC** | Baseline to 12-w | | Baseline to 24-w | | At 12-w | | At 24-w | |
|  | Change (95%CI) | *p* | Change (95%CI) | *p* | MD (95%CI) | *p* | MD (95%CI) | *p* |
| *Sex* |  |  |  |  |  |  |  |  |
| Boys | 91.61 (66.49 to 116.73) | 0.107 | 144.81 (118.96 to 170.66) | **0.043** | 27.99 (-6.06 to 62.04) | 0.953 | 34.09 (-0.50 to 68.68) | 0.942 |
| Girls | 61.82 (35.53 to 88.10) |  | 106.20 (79.10 to 133.29) |  | 26.50 (-10.00 to 63.01) |  | 35.95 (-1.63 to 73.54) |  |
| *Maturation stage* |  |  |  |  |  |  |  |  |
| Prepuberal | 54.94 (1.79 to 108.10) | 0.402 | 63.00 (0.68 to 125.33) | 0.225 | -26.03 (-132.28 to 80.22) | 0.507 | -43.57 (-154.68 to 67.55) | 0.842 |
| Peripuberal | 94.64 (71.05 to 118.22) |  | 160.29 (136.07 to 184.51) |  | 56.70 (22.37 to 91.03) |  | 63.73 (26.07 to 101.39) |  |
| Postpuberal | 54.75 (24.06 to 85.44) |  | 86.00 (53.90 to 118.10) |  | -1.71 (-40.77 to 37.35) |  | -2.73 (-42.89 to 37.44) |  |
| **BMC-TBLH** | Baseline to 12-w | | Baseline to 24-w | | At 12-w | | At 24-w | |
|  | Change (95%CI) | *p* | Change (95%CI) | *p* | MD (95%CI) | *p* | MD (95%CI) | *p* |
| *Sex* |  |  |  |  |  |  |  |  |
| Boys | 87.59 (63.39 to 111.80) | **0.046** | 132.79 (107.89 to 157.70) | **0.021** | 30.41 (-2.40 to 63.22) | 0.780 | 34.27 (0.94 to 67.60) | 0.971 |
| Girls | 51.92 (26.59 to 77.24) |  | 90.07 (63.96 to 116.17) |  | 23.61 (-11.56 to 58.79) |  | 33.37 (-2.85 to 69.59) |  |
| *Maturation stage* |  |  |  |  |  |  |  |  |
| Prepuberal | 53.43 (2.06 to 104.81) | 0.214 | 51.49 (-8.74 to 111.72) | 0.154 | -1.12 (-103.80 to 101.56) | 0.317 | -27.92 (-135.30 to 79.46) | 0.738 |
| Peripuberal | 89.72 (66.92 to 112.51) |  | 146.92 (123.52 to 170.33) |  | 56.50 (23.33 to 89.68) |  | 61.28 (24.88 to 97.68) |  |
| Postpuberal | 42.98 (13.31 to 72.64) |  | 69.19 (38.17 to 100.22) |  | -5.58 (-43.32 to 32.17) |  | -4.85 (-43.66 to 33.97) |  |
| **BMC-Arms** | Baseline to 12-w | | Baseline to 24-w | | At 12-w | | At 24-w | |
|  | Change (95%CI) | *p* | Change (95%CI) | *p* | MD (95%CI) | *p* | MD (95%CI) | *p* |
| *Sex* |  |  |  |  |  |  |  |  |
| Boys | 12.54 (3.14 to 21.94) | **0.040** | 26.30 (16.63 to 35.98) | 0.137 | 15.62 (2.87 to 28.36) | **0.010** | 14.40 (1.45 to 27.34) | 0.178 |
| Girls | -1.76 (-11.60 to 8.08) |  | 15.72 (5.58 to 25.86) |  | -8.98 (-22.65 to 4.68) |  | 1.31 (-12.76 to 15.37) |  |
| *Maturation stage* |  |  |  |  |  |  |  |  |
| Prepuberal | 3.63 (-17.13 to 24.38) | 0.945 | 0.77 (-23.53 to 25.08) | 0.910 | -6.17 (-47.49 to 35.15) | 0.136 | -11.27 (-54.48 to 31.94) | 0.726 |
| Peripuberal | 6.01 (-3.19 to 15.22) |  | 27.45 (18.01 to 36.89) |  | -0.33 (-13.73 to 13.06) |  | 16.96 (2.28 to 31.63) |  |
| Postpuberal | 5.50 (-6.48 to 17.49) |  | 5.50 (-6.48 to 17.49) |  | 12.10 (-3.14 to 27.35) |  | 6.92 (-8.75 to 22.58) |  |
| **BMC-Legs** | Baseline to 12-w | | Baseline to 24-w | | At 12-w | | At 24-w | |
|  | Change (95%CI) | *p* | Change (95%CI) | *p* | MD (95%CI) | *p* | MD (95%CI) | *p* |
| *Sex* |  |  |  |  |  |  |  |  |
| Boys | 35.54 (25.05 to 46.04) | 0.057 | 60.01 (49.21 to 70.81) | **0.002** | 14.90 (0.67 to 29.13) | 0.827 | 11.03 (-3.43 to 25.48) | 0.916 |
| Girls | 20.83 (9.85 to 31.81) |  | 34.83 (23.50 to 46.15) |  | 12.60 (-2.65 to 27.85) |  | 9.89 (-5.82 to 25.59) |  |
| *Maturation stage* |  |  |  |  |  |  |  |  |
| Prepuberal | 26.53 (4.44 to 48.62) | 0.057 | 28.21 (2.31 to 54.11) | **0.010** | 6.96 (-37.20 to 51.12) | 0.627 | -9.50 (-55.69 to 36.68) | 0.514 |
| Peripuberal | 37.69 (27.89 to 47.50) |  | 65.08 (55.01 to 75.14) |  | 21.15 (6.88 to 35.41) |  | 21.60 (5.94 to 37.25) |  |
| Postpuberal | 13.38 (0.63 to 26.14) |  | 21.98 (8.64 to 35.32) |  | 3.96 (-12.27 to 20.20) |  | -7.76 (-24.45 to 8.94) |  |
| **BMC-Pelvis** | Baseline to 12-w | | Baseline to 24-w | | At 12-w | | At 24-w | |
|  | Change (95%CI) | *p* | Change (95%CI) | *p* | MD (95%CI) | *p* | MD (95%CI) | *p* |
| *Sex* |  |  |  |  |  |  |  |  |
| Boys | 25.55 (16.25 to 34.86) | 0.108 | 27.77 (18.19 to 37.34) | 0.408 | 5.45 (-7.17 to 18.06) | 0.978 | 11.95 (-0.87 to 24.76) | 0.901 |
| Girls | 14.53 (4.79 to 24.26) |  | 21.96 (11.93 to 31.99) |  | 5.19 (-8.33 to 18.71) |  | 13.14 (-0.78 to 27.06) |  |
| *Maturation stage* |  |  |  |  |  |  |  |  |
| Prepuberal | 16.82 (-3.50 to 37.15) | 0.468 | 10.73 (-13.08 to 34.53) | 0.938 | 1.59 (-38.92 to 42.10) | 0.120 | 0.44 (-41.93 to 42.80) | 0.515 |
| Peripuberal | 24.31 (15.29 to 33.32) |  | 29.86 (20.61 to 39.11) |  | 16.82 (3.70 to 29.94) |  | 13.90 (-0.47 to 28.28) |  |
| Postpuberal | 14.36 (2.63 to 26.10) |  | 20.52 (8.25 to 32.79) |  | -6.65 (-21.58 to 8.28) |  | 9.86 (-5.48 to 25.21) |  |
| **BMC-Spine** | Baseline to 12-w | | Baseline to 24-w | | At 12-w | | At 24-w | |
|  | Change (95%CI) | *p* | Change (95%CI) | *p* | MD (95%CI) | *p* | MD (95%CI) | *p* |
| *Sex* |  |  |  |  |  |  |  |  |
| Boys | 8.06 (2.76 to 13.35) | 0.772 | 13.00 (7.55 to 18.45) | 0.305 | 2.37 (-4.81 to 9.56) | 0.725 | 5.10 (-2.19 to 12.40) | 0.404 |
| Girls | 6.93 (1.39 to 12.48) |  | 8.90 (3.18 to 14.61) |  | 0.50 (-7.20 to 8.20) |  | 0.56 (-7.37 to 8.48) |  |
| *Maturation stage* |  |  |  |  |  |  |  |  |
| Prepuberal | 2.27 (-9.11 to 13.64) | 0.956 | 5.43 (-7.89 to 18.75) | 0.463 | 2.52 (-20.13 to 25.18) | 0.464 | 3.43 (-20.26 to 27.12) | 0.452 |
| Peripuberal | 9.35 (4.31 to 14.40) |  | 15.03 (9.86 to 20.21) |  | 6.77 (-0.57 to 14.11) |  | 8.99 (0.95 to 17.04) |  |
| Postpuberal | 5.99 (-0.58 to 12.56) |  | 5.95 (-0.91 to 12.82) |  | -2.45 (-10.80 to 5.90) |  | -3.10 (-11.68 to 5.49) |  |
| **aBMD** | Baseline to 12-w | | Baseline to 24-w | | At 12-w | | At 24-w | |
|  | Change (95%CI) | *p* | Change (95%CI) | *p* | MD (95%CI) | *p* | MD (95%CI) | *p* |
| *Sex* |  |  |  |  |  |  |  |  |
| Boys | 0.02 (0.00 to 0.03) | 0.672 | 0.03 (0.02 to 0.05) | 0.244 | -0.01 (-0.03 to 0.01) | 0.209 | 0.00 (-0.02 to 0.02) | 0.849 |
| Girls | 0.02 (0.01 to 0.04) |  | 0.02 (0.01 to 0.04) |  | 0.50 (-7.20 to 8.20) |  | -0.00 (-0.02 to 0.02) |  |
| *Maturation stage* |  |  |  |  |  |  |  |  |
| Prepuberal | -0.00 (-0.03 to 0.03) | 0.879 | 0.02 (-0.02 to 0.05) | 0.112 | -0.00 (-0.06 to 0.06) | 0.510 | 0.00 (-0.06 to 0.06) | 0.216 |
| Peripuberal | 0.03 (0.01 to 0.04) |  | 0.04 (0.03 to 0.05) |  | 0.02 (-0.00 to 0.04) |  | 0.02 (-0.01 to 0.04) |  |
| Postpuberal | 0.01 (-0.00 to 0.03) |  | 0.01 (-0.01 to 0.03) |  | -0.01 (-0.03 to 0.01) |  | -0.02 (-0.05 to 0.00) |  |
| **aBMD-TBLH** | Baseline to 12-w | | Baseline to 24-w | | At 12-w | | At 24-w | |
|  | Change (95%CI) | *p* | Change (95%CI) | *p* | MD (95%CI) | *p* | MD (95%CI) | *p* |
| *Sex* |  |  |  |  |  |  |  |  |
| Boys | 0.02 (0.01 to 0.04) | 0.854 | 0.04 (0.02 to 0.05) | 0.079 | -0.00 (-0.02 to 0.02) | 0.392 | 0.01 (-0.01 to 0.03) | 0.571 |
| Girls | 0.02 (0.01 to 0.04) |  | 0.02 (0.00 to 0.03) |  | 0.01 (-0.01 to 0.03) |  | -0.00 (-0.02 to 0.02) |  |
| *Maturation stage* |  |  |  |  |  |  |  |  |
| Prepuberal | 0.01 (-0.02 to 0.04) | 0.504 | 0.02 (-0.02 to 0.05) | **0.049** | 0.01 (-0.05 to 0.07) | 0.237 | -0.00 (-0.07 to 0.06) | 0.220 |
| Peripuberal | 0.03 (0.02 to 0.04) |  | 0.04 (0.03 to 0.06) |  | 0.02 (0.00 to 0.04) |  | 0.02 (-0.00 to 0.04) |  |
| Postpuberal | 0.01 (-0.01 to 0.03) |  | 0.00 (-0.01 to 0.02) |  | -0.01 (-0.03 to 0.01) |  | -0.02 (-0.05 to 0.00) |  |
| **aBMD-Arms** | Baseline to 12-w | | Baseline to 24-w | | At 12-w | | At 24-w | |
|  | Change (95%CI) | *p* | Change (95%CI) | *p* | MD (95%CI) | *p* | MD (95%CI) | *p* |
| *Sex* |  |  |  |  |  |  |  |  |
| Boys | -0.01 (-0.05 to 0.03) | 0.445 | 0.00 (-0.04 to 0.04) | 0.254 | -0.03 (-0.09 to 0.02) | 0.404 | -0.04 (-0.09 to 0.01) | 0.893 |
| Girls | 0.01 (-0.03 to 0.05) |  | -0.03 (-0.07 to 0.01) |  | -0.00 (-0.06 to 0.05) |  | -0.04 (-0.10 to 0.01) |  |
| *Maturation stage* |  |  |  |  |  |  |  |  |
| Prepuberal | -0.06 (-0.15 to 0.02) | 0.652 | -0.06 (-0.16 to 0.04) | 0.368 | -0.08 (-0.25 to 0.09) | 0.839 | -0.08 (-0.25 to 0.10) | 0.314 |
| Peripuberal | 0.02 (-0.02 to 0.05) |  | 0.01 (-0.02 to 0.05) |  | 0.01 (-0.04 to 0.07) |  | 0.00 (-0.06 to 0.06) |  |
| Postpuberal | -0.01 (-0.06 to 0.04) |  | -0.06 (-0.11 to -0.00) |  | -0.04 (-0.10 to 0.02) |  | -0.09 (-0.15 to -0.02) |  |
| **aBMD-Legs** | Baseline to 12-w | | Baseline to 24-w | | At 12-w | | At 24-w | |
|  | Change (95%CI) | *p* | Change (95%CI) | *p* | MD (95%CI) | *p* | MD (95%CI) | *p* |
| *Sex* |  |  |  |  |  |  |  |  |
| Boys | 0.03 (0.02 to 0.05) | 0.535 | 0.06 (0.04 to 0.08) | 0.167 | 0.01 (-0.01 to 0.04) | 0.581 | 0.03 (0.01 to 0.06) | 0.606 |
| Girls | 0.03 (0.01 to 0.05) |  | 0.04 (0.02 to 0.06) |  | 0.02 (-0.00 to 0.05) |  | 0.02 (-0.01 to 0.05) |  |
| *Maturation stage* |  |  |  |  |  |  |  |  |
| Prepuberal | 0.05 (0.00 to 0.09) | 0.368 | 0.06 (0.01 to 0.11) | 0.075 | 0.06 (-0.02 to 0.14) | 0.600 | 0.05 (-0.04 to 0.13) | 0.384 |
| Peripuberal | 0.03 (0.01 to 0.05) |  | 0.03 (0.01 to 0.05) |  | 0.02 (-0.01 to 0.04) |  | 0.03 (0.00 to 0.06) |  |
| Postpuberal | 0.02 (0.00 to 0.05) |  | 0.03 (0.00 to 0.05) |  | 0.02 (-0.01 to 0.05) |  | 0.01 (-0.02 to 0.04) |  |
| **aBMD-Pelvis** | Baseline to 12-w | | Baseline to 24-w | | At 12-w | | At 24-w | |
|  | Change (95%CI) | *p* | Change (95%CI) | *p* | MD (95%CI) | *p* | MD (95%CI) | *p* |
| *Sex* |  |  |  |  |  |  |  |  |
| Boys | 0.04 (0.02 to 0.06) | 0.350 | 0.05 (0.03 to 0.07) | 0.957 | 0.02 (-0.00 to 0.04) | 0.355 | 0.04 (0.01 to 0.06) | 0.283 |
| Girls | 0.03 (0.01 to 0.05) |  | 0.05 (0.03 to 0.07) |  | 0.01 (-0.02 to 0.03) |  | 0.02 (-0.01 to 0.04) |  |
| *Maturation stage* |  |  |  |  |  |  |  |  |
| Prepuberal | 0.04 (0.00 to 0.07) | 0.260 | 0.03 (-0.01 to 0.07) | 0.792 | 0.01 (-0.07 to 0.08) | 0.526 | -0.02 (-0.10 to 0.06) | 0.100 |
| Peripuberal | 0.04 (0.02 to 0.06) |  | 0.06 (0.04 to 0.07) |  | 0.03 (0.01 to 0.05) |  | 0.03 (0.00 to 0.06) |  |
| Postpuberal | 0.02 (0.00 to 0.04) |  | 0.05 (0.03 to 0.07) |  | 0.00 (-0.03 to 0.03) |  | 0.03 (0.01 to 0.06) |  |
| **aBMD-Spine** | Baseline to 12-w | | Baseline to 24-w | | At 12-w | | At 24-w | |
|  | Change (95%CI) | *p* | Change (95%CI) | *p* | MD (95%CI) | *p* | MD (95%CI) | *p* |
| *Sex* |  |  |  |  |  |  |  |  |
| Boys | 0.04 (0.02 to 0.06) | 0.386 | 0.05 (0.03 to 0.07) | 0.319 | 0.01 (-0.02 to 0.04) | 0.280 | 0.01 (-0.02 to 0.03) | 0.263 |
| Girls | 0.03 (0.01 to 0.05) |  | 0.03 (0.01 to 0.05) |  | -0.01 (-0.04 to 0.02) |  | -0.02 (-0.05 to 0.01) |  |
| *Maturation stage* |  |  |  |  |  |  |  |  |
| Prepuberal | 0.03 (-0.01 to 0.08) | 0.454 | 0.02 (-0.03 to 0.07) | 0.778 | 0.01 (-0.08 to 0.10) | 0.112 | -0.01 (-0.11 to 0.08) | 0.770 |
| Peripuberal | 0.04 (0.02 to 0.06) |  | 0.05 (0.03 to 0.07) |  | 0.02 (-0.01 to 0.05) |  | 0.01 (-0.02 to 0.04) |  |
| Postpuberal | 0.02 (-0.00 to 0.05) |  | 0.03 (0.00 to 0.05) |  | -0.02 (-0.05 to 0.01) |  | -0.02 (-0.05 to 0.02) |  |
| The results are presented as the mean difference for each group and the mean difference between the groups. Data were analysed using linear-mixed models.  Bold letters indicate significant differences for between-category interaction effects.  *Abbreviations*: CI, confidence interval; BMI, body mass index; WC, waist circumference; ALMI, appendicular lean mass index; BMC, body mass content; TBLH, total body less head; aBMD, areal body mass density. | | | | | | | | |

# **Table ESM14.** Interaction of sex and maturation stage on both the effects within the Diactive-1 exercise group and the effects of the Diactive-1 exercise group versus the usual care group at 12 and 24 weeks using the per-protocol approach.

|  | **Within Diactive-1 exercise group** | | | | **Diactive-1 *vs* usual care** | | | |
| --- | --- | --- | --- | --- | --- | --- | --- | --- |
| **Body mass index** | Baseline to 12-w | | Baseline to 24-w | | At 12-w | | At 24-w | |
|  | Change (95%CI) | *p* | Change (95%CI) | *p* | MD (95%CI) | *p* | MD (95%CI) | *p* |
| *Sex* |  |  |  |  |  |  |  |  |
| Boys |  |  | 0.43 (-0.18 to 1.03) | 0.581 |  |  | 0.17 (-0.63 to 0.97) | 0.822 |
| Girls |  |  | 0.66 (0.06 to 1.27) |  |  |  | 0.30 (-0.54 to 1.14) |  |
| *Maturation stage* |  |  |  |  |  |  |  |  |
| Prepuberal |  |  | -0.33 (-1.87 to 1.20) | 0.876 |  |  | -0.19 (-2.85 to 2.47) | 0.852 |
| Peripuberal |  |  | 0.86 (0.28 to 1.45) |  |  |  | 0.45 (-0.45 to 1.35) |  |
| Postpuberal |  |  | 0.23 (-0.50 to 0.96) |  |  |  | -0.03 (-0.96 to 0.91) |  |
| **Waist circumference** | Baseline to 12-w | | Baseline to 24-w | | At 12-w | | At 24-w | |
|  | Change (95%CI) | *p* | Change (95%CI) | *p* | MD (95%CI) | *p* | MD (95%CI) | *p* |
| *Sex* |  |  |  |  |  |  |  |  |
| Boys |  |  | 1.70 (-0.70 to 4.10) | 0.435 |  |  | 2.54 (-0.65 to 5.73) | **0.041** |
| Girls |  |  | 0.37 (-2.03 to 2.77) |  |  |  | -2.27 (-5.60 to 1.07) |  |
| *Maturation stage* |  |  |  |  |  |  |  |  |
| Prepuberal |  |  | -2.81 (-9.19 to 3.56) | 0.924 |  |  | -0.98 (-11.97 to 10.01) | 0.550 |
| Peripuberal |  |  | 1.80 (-0.66 to 4.26) |  |  |  | 0.51 (-3.24 to 4.25) |  |
| Postpuberal |  |  | 0.22 (-2.86 to 3.31) |  |  |  | 0.61 (-3.31 to 4.54) |  |
| **WHtR** | Baseline to 12-w | | Baseline to 24-w | | At 12-w | | At 24-w | |
|  | Change (95%CI) | *p* | Change (95%CI) | *p* | MD (95%CI) | *p* | MD (95%CI) | *p* |
| *Sex* |  |  |  |  |  |  |  |  |
| Boys |  |  | 0.00 (-0.01 to 0.02) | 0.765 |  |  | 0.01 (-0.01 to 0.03) | 0.073 |
| Girls |  |  | -0.00 (-0.02 to 0.01) |  |  |  | -0.01 (-0.04 to 0.01) |  |
| *Maturation stage* |  |  |  |  |  |  |  |  |
| Prepuberal |  |  | -0.01 (-0.03 to 0.02) | 0.555 |  |  | 0.01 (-0.03 to 0.05) | 0.508 |
| Peripuberal |  |  | 0.00 (-0.02 to 0.02) |  |  |  | -0.01 (-0.03 to 0.02) |  |
| Postpuberal |  |  | 0.00 (-0.02 to 0.02) |  |  |  | 0.00 (-0.02 to 0.02) |  |
| **Fat mass** | Baseline to 12-w | | Baseline to 24-w | | At 12-w | | At 24-w | |
|  | Change (95%CI) | *p* | Change (95%CI) | *p* | MD (95%CI) | *p* | MD (95%CI) | *p* |
| *Sex* |  |  |  |  |  |  |  |  |
| Boys | 0.17 (-0.79 to 1.12) | 0.368 | 0.16 (-0.77 to 1.09) | 0.193 | -0.50 (-1.75 to 0.76) | 0.148 | -0.04 (-1.28 to 1.19) | 0.283 |
| Girls | 0.77 (-0.15 to 1.70) |  | 1.04 (0.09 to 2.00) |  | 0.82 (-0.46 to 2.11) |  | 0.94 (-0.38 to 2.27) |  |
| *Maturation stage* |  |  |  |  |  |  |  |  |
| Prepuberal | 0.61 (-1.37 to 2.58) | 0.515 | 0.16 (-2.15 to 2.46) | 0.405 | 1.17 (-2.75 to 5.10) | 0.162 | 1.03 (-3.08 to 5.13) | 0.387 |
| Peripuberal | 0.68 (-0.26 to 1.62) |  | 1.01 (0.10 to 1.92) |  | 0.84 (-0.48 to 2.16) |  | 0.95 (-0.46 to 2.35) |  |
| Postpuberal | 0.11 (-1.02 to 1.25) |  | -0.11 (-1.30 to 1.08) |  | -0.72 (-2.17 to 0.73) |  | -0.48 (-1.97 to 1.01) |  |
| **Arm fat mass** | Baseline to 12-w | | Baseline to 24-w | | At 12-w | | At 24-w | |
|  | Change (95%CI) | *p* | Change (95%CI) | *p* | MD (95%CI) | *p* | MD (95%CI) | *p* |
| *Sex* |  |  |  |  |  |  |  |  |
| Boys | 0.03 (-0.10 to 0.16) | 0.631 | 0.06 (-0.07 to 0.18) | 0.407 | 0.04 (-0.14 to 0.21) | 0.417 | 0.10 (-0.07 to 0.27) | 0.894 |
| Girls | -0.02 (-0.15 to 0.11) |  | 0.13 (0.00 to 0.26) |  | -0.07 (-0.24 to 0.11) |  | 0.08 (-0.10 to 0.27) |  |
| *Maturation stage* |  |  |  |  |  |  |  |  |
| Prepuberal | 0.04 (-0.23 to 0.31) | 0.795 | -0.07 (-0.38 to 0.25) | 0.531 | 0.03 (-0.51 to 0.56) | 0.771 | 0.03 (-0.54 to 0.59) | 0.858 |
| Peripuberal | -0.00 (-0.13 to 0.13) |  | 0.17 (0.04 to 0.29) |  | -0.04 (-0.22 to 0.14) |  | 0.15 (-0.04 to 0.34) |  |
| Postpuberal | -0.01 (-0.16 to 0.15) |  | -0.01 (-0.17 to 0.16) |  | 0.01 (-0.19 to 0.21) |  | 0.02 (-0.18 to 0.22) |  |
| **Leg fat mass** | Baseline to 12-w | | Baseline to 24-w | | At 12-w | | At 24-w | |
|  | Change (95%CI) | *p* | Change (95%CI) | *p* | MD (95%CI) | *p* | MD (95%CI) | *p* |
| *Sex* |  |  |  |  |  |  |  |  |
| Boys | 0.00 (-0.35 to 0.35) | 0.246 | 0.07 (-0.27 to 0.40) | 0.206 | -0.15 (-0.60 to 0.31) | 0.173 | -0.02 (-0.47 to 0.43) | 0.412 |
| Girls | 0.29 (-0.05 to 0.63) |  | 0.38 (0.03 to 0.73) |  | 0.31 (-0.16 to 0.78) |  | 0.25 (-0.23 to 0.74) |  |
| *Maturation stage* |  |  |  |  |  |  |  |  |
| Prepuberal | 0.05 (-0.67 to 0.78) | 0.729 | -0.02 (-0.87 to 0.83) | 0.600 | 0.41 (-1.04 to 1.85) | 0.344 | 0.44 (-1.07 to 1.95) | 0.549 |
| Peripuberal | 0.24 (-0.11 to 0.58) |  | 0.36 (0.02 to 0.69) |  | 0.29 (-0.19 to 0.77) |  | 0.25 (-0.26 to 0.77) |  |
| Postpuberal | 0.04 (-0.38 to 0.46) |  | 0.01 (-0.43 to 0.44) |  | -0.15 (-0.68 to 0.38) |  | -0.13 (-0.68 to 0.42) |  |
| **Trunk fat mass** | Baseline to 12-w | | Baseline to 24-w | | At 12-w | | At 24-w | |
|  | Change (95%CI) | *p* | Change (95%CI) | *p* | MD (95%CI) | *p* | MD (95%CI) | *p* |
| *Sex* |  |  |  |  |  |  |  |  |
| Boys | 0.13 (-0.43 to 0.69) | 0.354 | 0.02 (-0.52 to 0.56) | 0.199 | -0.40 (-1.13 to 0.34) | 0.068 | -0.15 (-0.87 to 0.57) | 0.156 |
| Girls | 0.49 (-0.05 to 1.04) |  | 0.53 (-0.03 to 1.09) |  | 0.58 (-0.17 to 1.34) |  | 0.61 (-0.16 to 1.39) |  |
| *Maturation stage* |  |  |  |  |  |  |  |  |
| Prepuberal | 0.50 (-0.65 to 1.65) | 0.405 | 0.21 (-1.14 to 1.56) | 0.368 | 0.78 (-1.51 to 3.06) | 0.060 | 0.57 (-1.82 to 2.96) | 0.282 |
| Peripuberal | 0.43 (-0.12 to 0.98) |  | 0.46 (-0.07 to 0.99) |  | 0.58 (-0.19 to 1.35) |  | 0.53 (-0.28 to 1.35) |  |
| Postpuberal | 0.08 (-0.59 to 0.74) |  | -0.11 (-0.81 to 0.59) |  | -0.57 (-1.42 to 0.27) |  | -0.37 (-1.24 to 0.50) |  |
| **Fat mass (%)** | Baseline to 12-w | | Baseline to 24-w | | At 12-w | | At 24-w | |
|  | Change (95%CI) | *p* | Change (95%CI) | *p* | MD (95%CI) | *p* | MD (95%CI) | *p* |
| *Sex* |  |  |  |  |  |  |  |  |
| Boys | -0.69 (-1.80 to 0.42) | 0.525 | -0.76 (-1.84 to 0.31) | 0.164 | -0.43 (-1.88 to 1.03) | 0.124 | -0.05 (-1.47 to 1.38) | 0.253 |
| Girls | -0.19 (-1.27 to 0.88) |  | 0.33 (-0.78 to 1.44) |  | 1.20 (-0.29 to 2.69) |  | 1.17 (-0.37 to 2.71) |  |
| *Maturation stage* |  |  |  |  |  |  |  |  |
| Prepuberal | -0.08 (-2.39 to 2.22) | 0.653 | -0.56 (-3.26 to 2.14) | 0.987 | 2.13 (-2.46 to 6.71) | 0.082 | 2.43 (-2.36 to 7.23) | 0.290 |
| Peripuberal | -0.39 (-1.49 to 0.71) |  | -0.18 (-1.24 to 0.89) |  | 1.03 (-0.51 to 2.57) |  | 0.92 (-0.72 to 2.56) |  |
| Postpuberal | -0.65 (-1.98 to 0.69) |  | -0.33 (-1.72 to 1.07) |  | -0.67 (-2.37 to 1.02) |  | -0.14 (-1.88 to 1.60) |  |
| **Visceral fat** | Baseline to 12-w | | Baseline to 24-w | | At 12-w | | At 24-w | |
|  | Change (95%CI) | *p* | Change (95%CI) | *p* | MD (95%CI) | *p* | MD (95%CI) | *p* |
| *Sex* |  |  |  |  |  |  |  |  |
| Boys | 10.47 (-28.62 to 49.56) | 0.580 | 20.23 (-17.73 to 58.19) | 0.212 | -27.49 (-79.25 to 24.28) | 0.375 | -3.48 (-53.90 to 46.95) | 0.943 |
| Girls | 26.51 (-15.37 to 68.40) |  | -13.77(-51.73 to 24.19) |  | 7.13 (-49.92 to 64.17) |  | -6.13 (-58.84 to 46.59) |  |
| *Maturation stage* |  |  |  |  |  |  |  |  |
| Prepuberal | 63.33 (-16.46 to 143.12) | 0.386 | 49.05 (-43.65 to 141.75) | 0.408 | 104.04 (-50.92 to 259.00) | 0.173 | 96.76 (-65.23 to 258.75) | 0.048 |
| Peripuberal | 9.51 (-32.42 to 51.45) |  | -0.70 (-37.41 to 36.01) |  | -11.32 (-68.05 to 45.41) |  | 25.81 (-29.18 to 80.80) |  |
| Postpuberal | 10.89 (-35.18 to 56.95) |  | -3.22 (-49.29 to 42.84) |  | -25.16 (-84.89 to 34.58) |  | -24.96 (-83.29 to 33.38) |  |
| **Subcutaneous fat** | Baseline to 12-w | | Baseline to 24-w | | At 12-w | | At 24-w | |
|  | Change (95%CI) | *p* | Change (95%CI) | *p* | MD (95%CI) | *p* | MD (95%CI) | *p* |
| *Sex* |  |  |  |  |  |  |  |  |
| Boys | 5.80 (-118.79 to 130.40) | **0.009** | -25.08 (-146.00 to 95.85) | 0.590 | -43.60 (-208.59 to 121.39) | 0.310 | -33.96 (-194.60 to 126.68) | 0.901 |
| Girls | -231.12 (-355.71 to -106.52) |  | -71.69 (-192.62 to 49.23) |  | -167.46 (-342.89 to 7.97) |  | -48.55 (-216.48 to 119.38) |  |
| *Maturation stage* |  |  |  |  |  |  |  |  |
| Prepuberal | 9.67 (-244.71 to 264.04) | 0.346 | -34.42 (-331.42 to 262.58) | 0.829 | 119.93 (-382.04 to 621.90) | 0.488 | 78.85 (-446.01 to 603.70) | 0.364 |
| Peripuberal | -231.33 (-356.13 to -106.53) |  | -55.98 (-173.37 to 61.41) |  | -143.93 (-318.66 to 30.80) |  | -3.47 (-179.92 to 172.97) |  |
| Postpuberal | 5.11 (-141.75 to 151.97) |  | -58.22 (-205.08 to 88.64) |  | -92.44 (-283.07 to 98.19) |  | -114.63 (-301.11 to 71.86) |  |
| **Lean mass** | Baseline to 12-w | | Baseline to 24-w | | At 12-w | | At 24-w | |
|  | Change (95%CI) | *p* | Change (95%CI) | *p* | MD (95%CI) | *p* | MD (95%CI) | *p* |
| *Sex* |  |  |  |  |  |  |  |  |
| Boys | 2.43 (1.59 to 3.27) | 0.487 | 2.72 (1.91 to 3.53) | 0.073 | 0.77 (-0.33 to 1.87) | 0.356 | 1.39 (0.31 to 2.47) | 0.156 |
| Girls | 2.02 (1.21 to 2.83) |  | 1.65 (0.82 to 2.49) |  | 0.03 (-1.10 to 1.16) |  | 0.24 (-0.92 to 1.41) |  |
| *Maturation stage* |  |  |  |  |  |  |  |  |
| Prepuberal | 1.73 (0.08 to 3.38) | 0.818 | 1.37 (-0.57 to 3.30) | 0.153 | 0.33 (-2.97 to 3.62) | 0.377 | -0.27 (-3.72 to 3.18) | 0.652 |
| Peripuberal | 2.34 (1.56 to 3.13) |  | 2.93 (2.17 to 3.70) |  | 0.30 (-0.80 to 1.40) |  | 0.94 (-0.24 to 2.12) |  |
| Postpuberal | 2.18 (1.23 to 3.13) |  | 1.18 (0.18 to 2.17) |  | 0.69 (-0.52 to 1.90) |  | 0.33 (-0.91 to 1.58) |  |
| **Arm lean mass** | Baseline to 12-w | | Baseline to 24-w | | At 12-w | | At 24-w | |
|  | Change (95%CI) | *p* | Change (95%CI) | *p* | MD (95%CI) | *p* | MD (95%CI) | *p* |
| *Sex* |  |  |  |  |  |  |  |  |
| Boys | 0.19 (-0.04 to 0.41) | 0.146 | 0.46 (0.24 to 0.68) | **0.05** | 0.26 (-0.04 to 0.56) | 0.068 | 0.24 (-0.05 to 0.54) | 0.189 |
| Girls | -0.05 (-0.27 to 0.17) |  | 0.14 (-0.08 to 0.37) |  | -0.14 (-0.44 to 0.17) |  | -0.04 (-0.36 to 0.27) |  |
| *Maturation stage* |  |  |  |  |  |  |  |  |
| Prepuberal | 0.09 (-0.37 to 0.56) | 0.839 | -0.04 (-0.59 to 0.50) | 0.784 | -0.04 (-0.96 to 0.89) | 0.309 | -0.16 (-1.13 to 0.81) | 0.700 |
| Peripuberal | 0.07 (-0.15 to 0.29) |  | 0.44 (0.23 to 0.66) |  | -0.00 (-0.31 to 0.31) |  | 0.35 (0.01 to 0.68) |  |
| Postpuberal | 0.05 (-0.22 to 0.31) |  | 0.16 (-0.12 to 0.44) |  | 0.17 (-0.17 to 0.51) |  | -0.04 (-0.40 to 0.31) |  |
| **Leg lean mass** | Baseline to 12-w | | Baseline to 24-w | | At 12-w | | At 24-w | |
|  | Change (95%CI) | *p* | Change (95%CI) | *p* | MD (95%CI) | *p* | MD (95%CI) | *p* |
| *Sex* |  |  |  |  |  |  |  |  |
| Boys | 0.90 (0.50 to 1.31) | 0.448 | 0.86 (0.47 to 1.25) | 0.404 | 0.23 (-0.30 to 0.76) | 0.408 | 0.42 (-0.10 to 0.95) | 0.364 |
| Girls | 0.69 (0.29 to 1.08) |  | 0.62 (0.22 to 1.03) |  | -0.08 (-0.63 to 0.46) |  | 0.07 (-0.49 to 0.63) |  |
| *Maturation stage* |  |  |  |  |  |  |  |  |
| Prepuberal | 0.53 (-0.28 to 1.35) | 0.672 | 0.59 (-0.37 to 1.54) | 0.264 | -0.00 (-1.63 to 1.63) | 0.577 | -0.10 (-1.80 to 1.60) | 0.769 |
| Peripuberal | 0.83 (0.44 to 1.22) |  | 0.97 (0.59 to 1.35) |  | 0.11 (-0.43 to 0.66) |  | 0.31 (-0.27 to 0.89) |  |
| Postpuberal | 0.81 (0.34 to 1.29) |  | 0.38 (-0.11 to 0.88) |  | 0.15 (-0.45 to 0.75) |  | 0.06 (-0.56 to 0.67) |  |
| **Trunk lean mass** | Baseline to 12-w | | Baseline to 24-w | | At 12-w | | At 24-w | |
|  | Change (95%CI) | *p* | Change (95%CI) | *p* | MD (95%CI) | *p* | MD (95%CI) | *p* |
| *Sex* |  |  |  |  |  |  |  |  |
| Boys | 1.28 (0.73 to 1.84) | 0.827 | 1.32 (0.78 to 1.86) | 0.263 | 0.22 (-0.51 to 0.95) | 0.909 | 0.63 (-0.09 to 1.34) | 0.512 |
| Girls | 1.37 (0.83 to 1.91) |  | 0.88 (0.33 to 1.44) |  | 0.28 (-0.47 to 1.03) |  | 0.28 (-0.49 to 1.05) |  |
| *Maturation stage* |  |  |  |  |  |  |  |  |
| Prepuberal | 1.06 (-0.06 to 2.18) | 0.865 | 0.70 (-0.61 to 2.01) | 0.382 | 0.66 (-1.57 to 2.89) | 0.710 | 0.17 (-2.17 to 2.50) | 0.458 |
| Peripuberal | 1.40 (0.87 to 1.93) |  | 1.43 (0.91 to 1.95) |  | 0.20 (-0.54 to 0.95) |  | 0.31 (-0.48 to 1.11) |  |
| Postpuberal | 1.30 (0.66 to 1.95) |  | 0.64 (-0.04 to 1.31) |  | 0.39 (-0.43 to 1.21) |  | 0.41 (-0.44 to 1.25) |  |
| **BMC** | Baseline to 12-w | | Baseline to 24-w | | At 12-w | | At 24-w | |
|  | Change (95%CI) | *p* | Change (95%CI) | *p* | MD (95%CI) | *p* | MD (95%CI) | *p* |
| *Sex* |  |  |  |  |  |  |  |  |
| Boys | 87.08 (60.01 to 114.14) | 0.184 | 142.82 (116.56 to 169.07) | 0.056 | 23.46 (-12.03 to 58.95) | 0.912 | 32.09 (-2.78 to 66.97) | 0.888 |
| Girls | 61.66 (35.40 to 87.91) |  | 106.04 (78.98 to 133.10) |  | 26.29 (-10.17 to 62.75) |  | 35.74 (-1.80 to 73.28) |  |
| *Maturation stage* |  |  |  |  |  |  |  |  |
| Prepuberal | 54.94 (1.85 to 108.04) | 0.471 | 63.13 (0.88 to 125.37) | 0.242 | -26.06 (-132.19 to 80.06) | 0.560 | -43.48 (-154.46 to 67.51) | 0.868 |
| Peripuberal | 90.81 (65.54 to 116.09) |  | 158.68 (134.10 to 183.25) |  | 52.87 (17.38 to 88.36) |  | 62.10 (24.23 to 99.96) |  |
| Postpuberal | 54.75 (24.09 to 85.40) |  | 86.00 (53.94 to 118.06) |  | -1.71 (-40.72 to 37.30) |  | -2.73 (-42.85 to 37.38) |  |
| **BMC-TBLH** | Baseline to 12-w | | Baseline to 24-w | | At 12-w | | At 24-w | |
|  | Change (95%CI) | *p* | Change (95%CI) | *p* | MD (95%CI) | *p* | MD (95%CI) | *p* |
| *Sex* |  |  |  |  |  |  |  |  |
| Boys | 82.35 (56.34 to 108.36) | 0.097 | 130.46 (105.23 to 155.70) | **0.029** | 25.16 (-8.95 to 59.27) | 0.944 | 31.94 (-1.59 to 65.46) | 0.960 |
| Girls | 51.77 (26.53 to 77.00) |  | 89.92 (63.91 to 115.93) |  | 23.43 (-11.61 to 58.47) |  | 33.18 (-2.90 to 69.27) |  |
| *Maturation stage* |  |  |  |  |  |  |  |  |
| Prepuberal | 53.43 (2.27 to 104.60) | 0.268 | 51.63 (-8.35 to 111.62) | 0.168 | -1.17 (-103.43 to 101.08) | 0.364 | -27.83 (-134.77 to 79.11) | 0.768 |
| Peripuberal | 85.11 (60.75 to 109.47) |  | 144.95 (121.27 to 168.63) |  | 51.89 (17.69 to 86.09) |  | 59.28 (22.80 to 95.77) |  |
| Postpuberal | 42.98 (13.44 to 72.52) |  | 69.20 (38.30 to 100.09) |  | -5.56 (-43.15 to 32.03) |  | -4.84 (-43.50 to 33.82) |  |
| **BMC-Arms** | Baseline to 12-w | | Baseline to 24-w | | At 12-w | | At 24-w | |
|  | Change (95%CI) | *p* | Change (95%CI) | *p* | MD (95%CI) | *p* | MD (95%CI) | *p* |
| *Sex* |  |  |  |  |  |  |  |  |
| Boys | 10.06 (-0.09 to 20.22) | 0.098 | 25.19 (15.34 to 35.04) | 0.183 | 13.14 (-0.17 to 26.45) | **0.023** | 13.29 (0.20 to 26.37) | 0.215 |
| Girls | -1.85 (-11.70 to 8.00) |  | 15.63 (5.48 to 25.78) |  | -9.09 (-22.77 to 4.59) |  | 1.20 (-12.88 to 15.28) |  |
| *Maturation stage* |  |  |  |  |  |  |  |  |
| Prepuberal | 3.63 (-16.96 to 24.22) | 0.804 | 0.91 (-23.20 to 25.01) | 0.857 | 12.17 (-2.96 to 27.29) | 0.103 | 6.96 (-8.58 to 22.51) | 0.683 |
| Peripuberal | 2.70 (-7.10 to 12.51) |  | 26.05 (16.52 to 35.57) |  | -3.67 (-17.43 to 10.09) |  | 15.50 (0.84 to 30.16) |  |
| Postpuberal | 5.50 (-6.38 to 17.39) |  | 15.57 (3.14 to 28.00) |  | -6.28 (-47.28 to 34.72) |  | -11.25 (-54.12 to 31.63) |  |
| **BMC-Legs** | Baseline to 12-w | | Baseline to 24-w | | At 12-w | | At 24-w | |
|  | Change (95%CI) | *p* | Change (95%CI) | *p* | MD (95%CI) | *p* | MD (95%CI) | *p* |
| *Sex* |  |  |  |  |  |  |  |  |
| Boys | 33.42 (22.05 to 44.79) | 0.117 | 59.09 (48.06 to 70.12) | **0.003** | 12.77 (-2.13 to 27.68) | 0.983 | 10.11 (-4.54 to 24.76) | 0.979 |
| Girls | 20.78 (9.76 to 31.81) |  | 34.78 (23.41 to 46.15) |  | 12.54 (-2.78 to 27.85) |  | 9.83 (-5.94 to 25.60) |  |
| *Maturation stage* |  |  |  |  |  |  |  |  |
| Prepuberal | 26.53 (4.31 to 48.75) | 0.077 | 28.26 (2.21 to 54.30) | **0.012** | 7.01 (-37.40 to 51.41) | 0.691 | -9.41 (-55.85 to 37.03) | 0.541 |
| Peripuberal | 36.03 (25.46 to 46.61) |  | 64.40 (54.12 to 74.68) |  | 19.50 (4.65 to 34.35) |  | 20.95 (5.10 to 36.79) |  |
| Postpuberal | 13.38 (0.56 to 26.21) |  | 21.98 (8.56 to 35.39) |  | 3.99 (-12.33 to 20.31) |  | -7.72 (-24.50 to 9.07) |  |
| **BMC-Pelvis** | Baseline to 12-w | | Baseline to 24-w | | At 12-w | | At 24-w | |
|  | Change (95%CI) | *p* | Change (95%CI) | *p* | MD (95%CI) | *p* | MD (95%CI) | *p* |
| *Sex* |  |  |  |  |  |  |  |  |
| Boys | 21.84 (12.08 to 31.59) | 0.281 | 26.09 (16.63 to 35.55) | 0.536 | 1.73 (-11.06 to 14.52) | 0.722 | 10.27 (-2.30 to 22.84) | 0.772 |
| Girls | 14.40 (4.94 to 23.87) |  | 21.84 (12.09 to 31.59) |  | 5.03 (-8.11 to 18.18) |  | 12.98 (-0.55 to 26.51) |  |
| *Maturation stage* |  |  |  |  |  |  |  |  |
| Prepuberal | 16.82 (-2.83 to 36.47) | 0.605 | 10.88 (-12.14 to 33.89) | 0.873 | 1.37 (-37.81 to 40.55) | 0.149 | 0.37 (-40.60 to 41.35) | 0.461 |
| Peripuberal | 20.61 (11.26 to 29.97) |  | 28.26 (19.17 to 37.34) |  | 13.06 (-0.07 to 26.19) |  | 12.19 (-1.81 to 26.18) |  |
| Postpuberal | 14.36 (3.02 to 25.71) |  | 20.52 (8.66 to 32.39) |  | -6.68 (-21.11 to 7.76) |  | 9.78 (-5.06 to 24.62) |  |
| **BMC-Spine** | Baseline to 12-w | | Baseline to 24-w | | At 12-w | | At 24-w | |
|  | Change (95%CI) | *p* | Change (95%CI) | *p* | MD (95%CI) | *p* | MD (95%CI) | *p* |
| *Sex* |  |  |  |  |  |  |  |  |
| Boys | 8.05 (2.40 to 13.70) | 0.759 | 13.03 (7.55 to 18.50) | 0.289 | 2.37 (-5.04 to 9.77) | 0.704 | 5.13 (-2.15 to 12.40) | 0.381 |
| Girls | 6.83 (1.35 to 12.31) |  | 8.79 (3.15 to 14.44) |  | 0.33 (-7.28 to 7.94) |  | 0.38 (-7.45 to 8.22) |  |
| *Maturation stage* |  |  |  |  |  |  |  |  |
| Prepuberal | 2.27 (-8.94 to 13.47) | 0.943 | 5.49 (-7.64 to 18.61) | 0.449 | 2.42 (-19.90 to 24.75) | 0.463 | 3.39 (-19.96 to 26.73) | 0.436 |
| Peripuberal | 9.49 (4.16 to 14.83) |  | 15.14 (9.96 to 20.32) |  | 6.86 (-0.62 to 14.35) |  | 9.02 (1.05 to 17.00) |  |
| Postpuberal | 5.99 (-0.48 to 12.46) |  | 5.95 (-0.81 to 12.71) |  | -2.54 (-10.78 to 5.69) |  | -3.24 (-11.70 to 5.22) |  |
| **aBMD** | Baseline to 12-w | | Baseline to 24-w | | At 12-w | | At 24-w | |
|  | Change (95%CI) | *p* | Change (95%CI) | *p* | MD (95%CI) | *p* | MD (95%CI) | *p* |
| *Sex* |  |  |  |  |  |  |  |  |
| Boys | 0.01 (-0.00 to 0.03) | 0.407 | 0.03 (0.02 to 0.05) | 0.323 | -0.01 (-0.03 to 0.01) | 0.123 | -0.00 (-0.02 to 0.02) | 0.947 |
| Girls | 0.02 (0.01 to 0.04) |  | 0.02 (0.01 to 0.04) |  | 0.01 (-0.01 to 0.03) |  | -0.00 (-0.02 to 0.02) |  |
| *Maturation stage* |  |  |  |  |  |  |  |  |
| Prepuberal | -0.00 (-0.03 to 0.03) | 0.763 | 0.02 (-0.02 to 0.05) | 0.126 | -0.00 (-0.06 to 0.06) | 0.580 | 0.00 (-0.06 to 0.07) | 0.232 |
| Peripuberal | 0.02 (0.01 to 0.04) |  | 0.04 (0.03 to 0.05) |  | 0.01 (-0.01 to 0.03) |  | 0.01 (-0.01 to 0.04) |  |
| Postpuberal | 0.01 (-0.00 to 0.03) |  | 0.01 (-0.01 to 0.03) |  | -0.01 (-0.03 to 0.01) |  | -0.02 (-0.05 to 0.00) |  |
| **aBMD-TBLH** | Baseline to 12-w | | Baseline to 24-w | | At 12-w | | At 24-w | |
|  | Change (95%CI) | *p* | Change (95%CI) | *p* | MD (95%CI) | *p* | MD (95%CI) | *p* |
| *Sex* |  |  |  |  |  |  |  |  |
| Boys | 0.02 (0.00 to 0.03) | 0.819 | 0.04 (0.02 to 0.05) | 0.114 | -0.01 (-0.03 to 0.02) | 0.256 | 0.00 (-0.02 to 0.02) | 0.658 |
| Girls | 0.02 (0.01 to 0.04) |  | 0.02 (0.00 to 0.03) |  | 0.01 (-0.01 to 0.03) |  | -0.00 (-0.02 to 0.02) |  |
| *Maturation stage* |  |  |  |  |  |  |  |  |
| Prepuberal | 0.01 (-0.01 to 0.03) | 0.605 | 0.00 (-0.01 to 0.02) | 0.056 | 0.01 (-0.05 to 0.07) | 0.282 | -0.00 (-0.07 to 0.06) | 0.237 |
| Peripuberal | 0.03 (0.01 to 0.04) |  | 0.04 (0.03 to 0.06) |  | 0.02 (-0.00 to 0.04) |  | 0.02 (-0.00 to 0.04) |  |
| Postpuberal | 0.01 (-0.02 to 0.04) |  | 0.02 (-0.02 to 0.05) |  | -0.01 (-0.03 to 0.01) |  | -0.02 (-0.05 to 0.00) |  |
| **aBMD-Arms** | Baseline to 12-w | | Baseline to 24-w | | At 12-w | | At 24-w | |
|  | Change (95%CI) | *p* | Change (95%CI) | *p* | MD (95%CI) | *p* | MD (95%CI) | *p* |
| *Sex* |  |  |  |  |  |  |  |  |
| Boys | -0.02 (-0.06 to 0.03) | 0.373 | 0.00 (-0.04 to 0.04) | 0.275 | -0.04 (-0.09 to 0.01) | 0.354 | -0.04 (-0.09 to 0.01) | 0.908 |
| Girls | 0.01 (-0.03 to 0.05) |  | -0.03 (-0.07 to 0.01) |  | -0.00 (-0.06 to 0.05) |  | -0.04 (-0.10 to 0.01) |  |
| *Maturation stage* |  |  |  |  |  |  |  |  |
| Prepuberal | -0.06 (-0.15 to 0.02) | 0.620 | -0.06 (-0.16 to 0.04) | 0.382 | -0.08 (-0.25 to 0.09) | 0.873 | -0.07 (-0.25 to 0.10) | 0.327 |
| Peripuberal | 0.02 (-0.03 to 0.06) |  | 0.02 (-0.02 to 0.06) |  | 0.01 (-0.04 to 0.07) |  | 0.00 (-0.06 to 0.07) |  |
| Postpuberal | -0.01 (-0.06 to 0.04) |  | -0.06 (-0.11 to -0.00) |  | -0.04 (-0.10 to 0.03) |  | -0.09 (-0.15 to -0.02) |  |
| **aBMD-Legs** | Baseline to 12-w | | Baseline to 24-w | | At 12-w | | At 24-w | |
|  | Change (95%CI) | *p* | Change (95%CI) | *p* | MD (95%CI) | *p* | MD (95%CI) | *p* |
| *Sex* |  |  |  |  |  |  |  |  |
| Boys | 0.03 (0.01 to 0.05) | 0.996 | 0.06 (0.04 to 0.08) | 0.254 | 0.01 (-0.02 to 0.03) | 0.317 | 0.03 (0.00 to 0.05) | 0.739 |
| Girls | 0.03 (0.01 to 0.05) |  | 0.04 (0.02 to 0.06) |  | 0.02 (-0.00 to 0.05) |  | 0.02 (-0.01 to 0.05) |  |
| *Maturation stage* |  |  |  |  |  |  |  |  |
| Prepuberal | 0.05 (0.01 to 0.09) | 0.489 | 0.06 (0.01 to 0.11) | 0.084 | 0.06 (-0.02 to 0.14) | 0.710 | 0.05 (-0.04 to 0.13) | 0.411 |
| Peripuberal | 0.02 (0.00 to 0.04) |  | 0.06 (0.04 to 0.08) |  | 0.01 (-0.02 to 0.04) |  | 0.03 (0.00 to 0.06) |  |
| Postpuberal | 0.02 (0.00 to 0.05) |  | 0.03 (0.01 to 0.05) |  | 0.02 (-0.01 to 0.05) |  | 0.01 (-0.02 to 0.04) |  |
| **aBMD-Pelvis** | Baseline to 12-w | | Baseline to 24-w | | At 12-w | | At 24-w | |
|  | Change (95%CI) | *p* | Change (95%CI) | *p* | MD (95%CI) | *p* | MD (95%CI) | *p* |
| *Sex* |  |  |  |  |  |  |  |  |
| Boys | 0.04 (0.02 to 0.05) | 0.540 | 0.05 (0.03 to 0.07) | 0.862 | 0.02 (-0.01 to 0.04) | 0.486 | 0.03 (0.01 to 0.06) | 0.319 |
| Girls | 0.03 (0.01 to 0.05) |  | 0.05 (0.03 to 0.07) |  | 0.01 (-0.02 to 0.03) |  | 0.02 (-0.01 to 0.04) |  |
| *Maturation stage* |  |  |  |  |  |  |  |  |
| Prepuberal | 0.04 (0.00 to 0.07) | 0.317 | 0.03 (-0.01 to 0.07) | 0.754 | 0.01 (-0.07 to 0.08) | 0.587 | -0.02 (-0.09 to 0.06) | 0.089 |
| Peripuberal | 0.04 (0.02 to 0.05) |  | 0.06 (0.04 to 0.07) |  | 0.03 (0.00 to 0.05) |  | 0.03 (0.00 to 0.05) |  |
| Postpuberal | 0.02 (0.00 to 0.04) |  | 0.05 (0.03 to 0.07) |  | 0.00 (-0.02 to 0.03) |  | 0.03 (0.01 to 0.06) |  |
| **aBMD-Spine** | Baseline to 12-w | | Baseline to 24-w | | At 12-w | | At 24-w | |
|  | Change (95%CI) | *p* | Change (95%CI) | *p* | MD (95%CI) | *p* | MD (95%CI) | *p* |
| *Sex* |  |  |  |  |  |  |  |  |
| Boys | 0.04 (0.02 to 0.06) | 0.335 | 0.05 (0.03 to 0.07) | 0.283 | 0.01 (-0.02 to 0.04) | 0.244 | 0.01 (-0.02 to 0.04) | 0.234 |
| Girls | 0.03 (0.01 to 0.05) |  | 0.03 (0.01 to 0.05) |  | -0.01 (-0.04 to 0.02) |  | -0.02 (-0.05 to 0.01) |  |
| *Maturation stage* |  |  |  |  |  |  |  |  |
| Prepuberal | 0.03 (-0.01 to 0.08) | 0.452 | 0.02 (-0.03 to 0.07) | 0.775 | 0.01 (-0.08 to 0.10) | 0.111 | -0.01 (-0.11 to 0.08) | 0.760 |
| Peripuberal | 0.04 (0.02 to 0.06) |  | 0.05 (0.03 to 0.07) |  | 0.02 (-0.01 to 0.05) |  | 0.01 (-0.02 to 0.04) |  |
| Postpuberal | 0.02 (-0.00 to 0.05) |  | 0.03 (0.00 to 0.05) |  | -0.02 (-0.06 to 0.01) |  | -0.02 (-0.05 to 0.02) |  |
| The results are presented as the mean difference for each group and the mean difference between the groups. Data were analysed using linear-mixed models.  Bold letters indicate significant differences for between-category interaction effects.  *Abbreviations*: CI, confidence interval; BMI, body mass index; WC, waist circumference; ALMI, appendicular lean mass index; BMC, body mass content; TBLH, total body less head; aBMD, areal body mass density. | | | | | | | | |

# **Table ESM15.** Within-group and between-group differences (Diactive-1 and usual care) resulting from restricted longitudinal data models (cLDA), limiting the means of the baseline results so that they were equal in all groups.

|  | Within-group differences (Diactive-1; *n*=30) | | | Within-group differences (Usual care; *n*=32) | | | Between-groups differences | | |
| --- | --- | --- | --- | --- | --- | --- | --- | --- | --- |
| Variables | Change  (95% CI) | Change %^Ψ^ | *p*  value | Change  (95% CI) | Change %^Ψ^ | *p*  value | Mean difference  (95% CI) | Hedges' *g* | *p*  value |
| **Anthropometric** | | | | | | | | | |
| BMI (*kg/m^2^*) |  |  |  |  |  |  |  |  |  |
| *Baseline to 24-w* | 0.54 ( 0.03 to 1.04) | 2.586 | **0.023** | 0.31 (-0.15 to 0.77) | 1.484 | 0.193 | 0.24 (-0.33 to 0.80) | 0.209 | 0.412 |
| WC (*cm*) |  |  |  |  |  |  |  |  |  |
| *Baseline to 12-w* | 0.94 (-1.15 to 3.02) | 1.364 | 0.433 | 0.73 (-1.18 to 2.64) | 1.059 | 0.529 | 0.21 (-2.12 to 2.54) | 0.045 | 0.859 |
| WHtR (score) |  |  |  |  |  |  |  |  |  |
| *Baseline to 24-w* | -0.03 (-1.31 to 1.24) | -0.070 | 0.991 | -0.04 (-1.21 to 1.14) | -0.093 | 0.988 | 0.00 (-1.43 to 1.43) | 0.000 | 0.997 |
| **Body composition (DXA)** | | | | | | | | | |
| Fat mass (*kg*) |  |  |  |  |  |  |  |  |  |
| *Baseline to 12-w* | 0.41 (-0.34 to 1.16) | 2.580 | 0.392 | 0.34 (-0.37 to 1.04) | 2.139 | 0.489 | 0.07 (-0.78 to 0.93) | 0.043 | 0.866 |
| *12-w to 24-w* | 0.07 (-0.51 to 0.65) | 0.440 | 0.950 | -0.16 (-0.68 to 0.35) | -1.006 | 0.734 | 0.24 (-0.41 to 0.88) | 0.185 | 0.467 |
| *Baseline to 24-w* | 0.48 (-0.47 to 1.44) | 3.020 | 0.447 | 0.18 (-0.71 to 1.07) | 1.132 | 0.883 | 0.31 (-0.78 to 1.39) | 0.144 | 0.572 |
| Arm fat mass *(kg)* |  |  |  |  |  |  |  |  |  |
| *Baseline to 12-w* | 0.01 (-0.091 to 0.119) | 0.546 | 0.944 | 0.02 (-0.081 to 0.117) | 1.092 | 0.897 | 0.00 (-0.124 to 0.116) | 0.000 | 0.943 |
| *12-w to 24-w* | 0.09 (-0.019 to 0.194) | 4.918 | 0.128 | -0.02 (-0.113 to 0.078) | -1.092 | 0.897 | 0.10 (-0.032 to 0.233) | 0.448 | 0.082 |
| *Baseline to 24-w* | 0.10 (-0.017 to 0.219) | 5.464 | 0.106 | 0.00 (-0.107 to 0.109) | 0.000 | 0.999 | 0.10 (-0.014 to 0.223) | 0.384 | 0.135 |
| Leg fat mass *(kg)* |  |  |  |  |  |  |  |  |  |
| *Baseline to 12-w* | 0.13 (-0.121 to 0.388) | 1.859 | 0.422 | 0.07 (-0.174 to 0.308) | 1.001 | 0.782 | 0.07 (-0.224 to 0.357) | 0.117 | 0.646 |
| *12-w to 24-w* | 0.04 (-0.189 to 0.273) | 0.572 | 0.900 | 0.04 (-0.163 to 0.248) | 0.572 | 0.873 | 0.00 (-0.339 to 0.471) | 0.000 | 0.996 |
| *Baseline to 24-w* | 0.18 (-0.181 to 0.532) | 2.575 | 0.467 | 0.11 (-0.222 to 0.441) | 1.573 | 0.708 | 0.07 (-0.256 to 0.255) | 0.083 | 0.744 |
| Trunk fat mass *(kg)* |  |  |  |  |  |  |  |  |  |
| *Baseline to 12-w* | 0.25 (-0.219 to 0.728) | 3.993 | 0.404 | 0.25 (-0.200 to 0.694) | 3.993 | 0.384 | 0.01 (-0.534 to 0.550) | 0.007 | 0.977 |
| *12-w to 24-w* | -0.06 (-0.389 to 0.274) | -0.958 | 0.908 | -0.19 (-0.491 to 0.101) | -3.035 | 0.260 | 0.14 (-0.228 to 0.503) | 0.191 | 0.454 |
| *Baseline to 24-w* | 0.20 (-0.348 to 0.743) | 3.194 | 0.661 | 0.05 (-0.458 to 0.563) | 0.798 | 0.967 | 0.15 (-0.474 to 0.765) | 0.119 | 0.640 |
| Fat mass (*%*) |  |  |  |  |  |  |  |  |  |
| *Baseline to 12-w* | -0.56 (-1.43 to 0.31) | -1.986 | 0.279 | -0.77 (-1.59 to 0.05) | -2.731 | 0.073 | 0.21 (-0.78 to 1.20) | 0.105 | 0.679 |
| *12-w to 24-w* | 0.21 (-0.52 to 0.94) | 0.744 | 0.776 | 0.03 (-0.63 to 0.68) | 0.106 | 0.196 | 0.18 (-0.64 to 1.00) | 0.111 | 0.662 |
| *Baseline to 24-w* | -0.35 (-1.44 to 0.73) | -1.241 | 0.718 | -0.74 (-1.75 to 0.27) | -2.625 | 0.994 | 0.39 (-0.85 to 1.62) | 0.158 | 0.535 |
| Visceral fat (*g*) |  |  |  |  |  |  |  |  |  |
| *Baseline to 12-w* | 17.29 (-21.612 to 56.190) | 12.419 | 0.535 | 29.12 ( -6.004 to 64.241) | 20.916 | 0.122 | -11.83 (-55.13 to 31.47) | -0.139 | 0.585 |
| *12-w to 24-w* | -16.86 (-50.539 to 16.826) | -12.110 | 0.451 | -19.71 (-48.836 to 9.416) | -14.157 | 0.240 | 2.85 (-34.29 to 39.99) | 0.039 | 0.877 |
| *Baseline to 24-w* | 0.43 (-28.913 to 29.778) | 0.308 | 0.999 | 9.41 (-17.090 to 35.907) | 6.759 | 0.671 | -8.98 (-41.10 to 23.15) | -0.142 | 0.577 |
| Subcutaneous fat (*g*) |  |  |  |  |  |  |  |  |  |
| *Baseline to 12-w* | -90.48 (-212.59 to 31.63) | -12.677 | 0.183 | -0.61 (-113.94 to 112.73) | -0.085 | 0.999 | -89.87 (-227.78 to  48.03) | -0.331 | 0.196 |
| *12-w to 24-w* | 49.36 ( -58.40 to 157.11) | 6.915 | 0.514 | -7.25 (-106.71 to 92.21) | -1.015 | 0.983 | 56.60 ( -65.14 to 178.35) | 0.236 | 0.354 |
| *Baseline to 24-w* | -41.12 (-133.63 to 51.38) | -5.761 | 0.536 | -7.86 ( -92.66 to 76.95) | -1.101 | 0.973 | -33.27 (-136.18 to  69.64) | -0.164 | 0.519 |
| Lean mass (*kg*) |  |  |  |  |  |  |  |  |  |
| *Baseline to 12-w* | 2.31 ( 1.69 to 2.93) | 6.138 | **<0.001** | 1.82 (1.24 to 2.40) | 4.836 | **<0.001** | 0.49 (-0.21 to 1.19) | 0.354 | 0.167 |
| *12-w to 24-w* | 0.01 (-0.60 to 0.61) | 0.026 | 0.999 | -0.46 (-1.00 to 0.07) | -1.222 | 0.106 | 0.47 (-0.21 to 1.15) | 0.352 | 0.170 |
| *Baseline to 24-w* | 2.32 ( 1.47 to 3.17) | 6.165 | **<0.001** | 1.36 (0.56 to 2.14) | 3.614 | **0.001** | 0.96 (-0.01 to 1.93) | 0.504 | 0.051 |
| Arm lean mass *(kg)* |  |  |  |  |  |  |  |  |  |
| *Baseline to 12-w* | 0.08 (-0.093 to 0.262) | 2.000 | 0.489 | 0.01 (-0.157 to 0.170) | 0.250 | 0.995 | 0.08 (-0.124 to 0.280) | 0.196 | 0.441 |
| *12-w to 24-w* | 0.25 ( 0.036 to 0.470) | 6.250 | **0.018** | 0.19 (-0.011 to 0.384) | 4.750 | 0.068 | 0.07 (-0.065 to 0.354) | 0.141 | 0.580 |
| *Baseline to 24-w* | 0.34 ( 0.150 to 0.525) | 8.500 | **<0.001** | 0.19 ( 0.021 to 0.365) | 4.750 | **0.024** | 0.14 (-0.174 to 0.307) | 0.350 | 0.172 |
| Leg lean mass *(kg)* |  |  |  |  |  |  |  |  |  |
| *Baseline to 12-w* | 0.84 ( 0.537 to 1.149) | 6.334 | **<0.001** | 0.72 ( 0.432 to 1.005) | 5.429 | **<0.001** | 0.12 (-0.226 to 0.475) | 0.181 | 0.478 |
| *12-w to 24-w* | -0.10 (-0.447 to 0.238) | -0.754 | 0.744 | -0.22 (-0.528 to 0.089) | -1.659 | 0.209 | 0.11 (-0.268 to 0.497) | 0.152 | 0.550 |
| *Baseline to 24-w* | 0.74 ( 0.388 to 1.089) | 5.580 | **<0.001** | 0.50 ( 0.177 to 0.821) | 3.770 | **0.001** | 0.24 (-0.154 to 0.633) | 0.309 | 0.227 |
| Trunk lean mass *(kg)* |  |  |  |  |  |  |  |  |  |
| *Baseline to 12-w* | 1.33 ( 0.907 to 1.745) | 7.674 | **<0.001** | 1.08 ( 0.694 to 1.474) | 6.231 | **<0.001** | 0.24 (-0.234 to 0.718) | 0.258 | 0.312 |
| *12-w to 24-w* | -0.21 (-0.709 to 0.285) | -1.211 | 0.562 | -0.42 (-0.871 to 0.029) | -2.423 | 0.071 | 0.21 (-0.349 to 0.767) | 0.190 | 0.455 |
| *Baseline to 24-w* | 1.11 ( 0.649 to 1.579) | 6.405 | **<0.001** | 0.66 ( 0.238 to 1.089) | 3.808 | **0.001** | 0.45 (-0.075 to 0.977) | 0.435 | 0.091 |
| BMC (*g*) |  |  |  |  |  |  |  |  |  |
| *Baseline to 12-w* | 78.85 (57.72 to  99.96) | 3.846 | **<0.001** | 50.42 (30.40 to  70.43) | 2.459 | **<0.001** | 28.43 (4.18 to 52.67) | 0.595 | **0.022** |
| *12-w to 24-w* | 48.07 (32.27 to  63.86) | 2.345 | **<0.001** | 39.77 (25.50 to  54.03) | 1.940 | **<0.001** | 8.30 (-9.46 to 26.07) | 0.237 | 0.352 |
| *Baseline to 24-w* | 126.92 (96.32 to 157.51) | 6.191 | **<0.001** | 90.19 (61.33 to 119.04) | 4.399 | **<0.001** | 36.73 (1.65 to 71.80) | 0.532 | **0.040** |
| BMC-TBLH (*g*) |  |  |  |  |  |  |  |  |  |
| *Baseline to 12-w* | 71.58 (51.301 to 91.855) | 4.393 | **<0.001** | 44.01 (24.812 to 63.205) | 2.701 | **<0.001** | 27.57 (4.273 to 50.866) | 0.600 | **0.021** |
| *12-w to 24-w* | 41.53 (25.221 to 57.843) | 2.549 | **<0.001** | 33.68 (18.959 to 48.402) | 2.067 | **<0.001** | 7.85 (-10.497 to 26.200) | 0.218 | 0.393 |
| *Baseline to 24-w* | 113.11 (83.078 to 143.142) | 6.942 | **<0.001** | 77.69 (49.398 to 105.980) | 4.768 | **<0.001** | 35.42 (1.005 to 69.837) | 0.524 | **0.043** |
| BMC-Arms (*g*) |  |  |  |  |  |  |  |  |  |
| *Baseline to 12-w* | 4.68 (-3.628 to 12.990) | 1.837 | 0.370 | 2.31 (-5.435 to 10.060) | 0.907 | 0.753 | 2.37 (-7.000 to 11.737) | 0.128 | 0.614 |
| *12-w to 24-w* | 16.07 ( 6.340 to 25.797) | 6.310 | **<0.001** | 10.83 ( 1.937 to 19.717) | 4.252 | **0.013** | 5.24 (-5.742 to 16.224) | 0.242 | 0.342 |
| *Baseline to 24-w* | 20.75 (12.945 to 28.554) | 8.148 | **<0.001** | 13.14 ( 6.025 to 20.255) | 5.159 | **<0.001** | 7.61 (-1.188 to 16.407) | 0.439 | 0.088 |
| BMC-Legs (*g*) |  |  |  |  |  |  |  |  |  |
| *Baseline to 12-w* | 28.52 (20.176 to 36.860) | 3.602 | **<0.001** | 15.23 ( 7.347 to 23.111) | 1.923 | **<0.001** | 13.29 (3.715 to 22.864) | 0.707 | **0.007** |
| *12-w to 24-w* | 20.26 (11.957 to 28.557) | 2.558 | **<0.001** | 22.73 (15.309 to 30.161) | 2.870 | **<0.001** | -2.48 (-11.789 to 6.834) | -0.366 | 0.154 |
| *Baseline to 24-w* | 48.78 (35.642 to 61.910) | 6.161 | **<0.001** | 37.96 (25.676 to 50.251) | 4.794 | **<0.001** | 10.81 (-4.209 to 25.833) | 0.135 | 0.595 |
| BMC-Pelvis (*g*) |  |  |  |  |  |  |  |  |  |
| *Baseline to 12-w* | 21.08 (11.478 to 30.689) | 8.266 | **<0.001** | 15.07 (6.059 to 24.088) | 5.909 | **<0.001** | 6.01 (-4.942 to 16.962) | 0.278 | 0.276 |
| *12-w to 24-w* | 4.03 (-3.645 to 11.714) | 1.580 | 0.419 | -2.43 (-9.352 to 4.491) | -0.952 | 0.675 | 6.47 (-2.143 to 15.074) | 0.382 | 0.137 |
| *Baseline to 24-w* | 25.12 (17.366 to 32.869) | 9.850 | **<0.001** | 12.64 (5.448 to 19.837) | 4.956 | **<0.001** | 12.48 ( 3.675 to 21.276) | 0.722 | **0.006** |
| BMC-Spine (*g*) |  |  |  |  |  |  |  |  |  |
| *Baseline to 12-w* | 7.66 (2.728 to 12.592) | 5.743 | **0.001** | 6.05 (1.413 to 10.685) | 4.536 | **0.007** | 1.61 (-4.036 to 7.259) | 0.145 | 0.570 |
| *12-w to 24-w* | 3.08 (-1.084 to 7.235) | 2.309 | 0.184 | 2.00 (-1.740 to 5.740) | 1.499 | 0.406 | 1.08 (-2.938 to 8.311) | 0.118 | 0.643 |
| *Baseline to 24-w* | 10.74 ( 5.772 to 15.699) | 8.052 | **<0.001** | 8.05 ( 3.437 to 12.660) | 6.035 | **<0.001** | 2.69 (-3.562 to 5.713) | 0.243 | 0.341 |
| aBMD (*g/cm^2^*) |  |  |  |  |  |  |  |  |  |
| *Baseline to 12-w* | 0.02 (0.01 to 0.03) | 1.923 | **<0.001** | 0.02 (0.01 to 0.02) | 1.923 | 0.002 | 0.00 (-0.01 to 0.01) | 0.000 | 0.662 |
| *12-w to 24-w* | 0.01 (-0.01 to 0.01) | 0.961 | 0.408 | 0.01 (0.00 to 0.02) | 0.961 | 0.047 | 0.00 (-0.01 to 0.01) | 0.000 | 0.493 |
| *Baseline to 24-w* | 0.03 (0.01 to 0.04) | 2.884 | **<0.001** | 0.03 (0.01 to 0.04) | 2.884 | **<0.001** | 0.00 (-0.01 to 0.01) | 0.000 | 0.857 |
| aBMD-TBLH (*g/cm^2^*) |  |  |  |  |  |  |  |  |  |
| *Baseline to 12-w* | 0.02 ( 0.010 to 0.036) | 2.127 | **<0.001** | 0.02 ( 0.005 to 0.029) | 2.127 | **0.003** | 0.01 (-0.008 to 0.021) | 0.214 | 0.402 |
| *12-w to 24-w* | 0.01 (-0.007 to 0.017) | 1.063 | 0.588 | 0.01 ( 0.000 to 0.022) | 1.063 | 0.062 | 0.00 (-0.015 to 0.016) | 0.000 | 0.404 |
| *Baseline to 24-w* | 0.03 ( 0.015 to 0.042) | 3.191 | **<0.001** | 0.03 ( 0.015 to 0.040) | 3.191 | **<0.001** | -0.01 (-0.019 to 0.008) | -0.018 | 0.943 |
| aBMD-Arms (*g/cm^2^*) |  |  |  |  |  |  |  |  |  |
| *Baseline to 12-w* | 0.00 (-0.032 to 0.033) | 0.000 | 0.999 | 0.02 (-0.010 to 0.050) | 2.816 | 0.262 | -0.02 (-0.056 to 0.018) | -0.265 | 0.299 |
| *12-w to 24-w* | -0.02 (-0.050 to 0.018) | -2.816 | 0.500 | 0.01 (-0.020 to 0.041) | 1.408 | 0.663 | -0.03 (-0.064 to 0.011) | -0.363 | 0.157 |
| *Baseline to 24-w* | -0.02 (-0.052 to 0.022) | -2.816 | 0.580 | 0.03 (-0.003 to 0.065) | 4.225 | 0.084 | -0.05 (-0.087 to -0.005) | -0.570 | **0.028** |
| aBMD-Legs (*g/cm^2^*) |  |  |  |  |  |  |  |  |  |
| *Baseline to 12-w* | 0.03 ( 0.015 to 0.048) | 2.678 | **<0.001** | 0.01 (-0.003 to 0.028) | 0.892 | 0.135 | 0.02 (0.000 to 0.038) | 0.514 | **0.047** |
| *12-w to 24-w* | 0.02 ( 0.002 to 0.036) | 1.785 | **0.025** | 0.02 (0.000 to 0.031) | 1.785 | **0.047** | 0.00 (-0.015 to 0.022) | 0.000 | 0.706 |
| *Baseline to 24-w* | 0.05 ( 0.035 to 0.066) | 4.464 | **<0.001** | 0.03 (0.014 to 0.042) | 2.678 | **<0.001** | 0.02 (0.005 to 0.040) | 0.665 | **0.011** |
| aBMD-Pelvis (*g/cm^2^*) |  |  |  |  |  |  |  |  |  |
| *Baseline to 12-w* | 0.03 ( 0.018 to 0.048) | 3.225 | **<0.001** | 0.02 ( 0.006 to 0.035) | 2.150 | **0.002** | 0.01 (-0.004 to  0.030) | 0.375 | 0.144 |
| *12-w to 24-w* | 0.02 ( 0.004 to 0.034) | 2.150 | **0.012** | 0.00 (-0.010 to 0.018) | 0.000 | 0.731 | 0.01 (-0.003 to 0.032) | 0.427 | 0.097 |
| *Baseline to 24-w* | 0.05 ( 0.037 to 0.067) | 5.376 | **<0.001** | 0.02 ( 0.011 to 0.039) | 2.150 | **<0.001** | 0.03 (0.010 to 0.044) | 0.819 | **0.002** |
| aBMD-Spine (*g/cm^2^*) |  |  |  |  |  |  |  |  |  |
| *Baseline to 12-w* | 0.03 ( 0.014 to 0.056) | 3.370 | **<0.001** | 0.03 ( 0.015 to 0.054) | 3.370 | **<0.001** | 0.00 (-0.024 to 0.024) | 0.000 | 0.977 |
| *12-w to 24-w* | 0.00 (-0.012 to 0.021) | 0.000 | 0.786 | 0.01 (-0.006 to 0.024) | 1.123 | 0.331 | 0.00 (-0.023 to 0.014) | 0.000 | 0.637 |
| *Baseline to 24-w* | 0.04 ( 0.025 to 0.053) | 4.494 | **<0.001** | 0.04 ( 0.030 to 0.056) | 4.494 | **<0.001** | 0.00 (-0.020 to 0.012) | 0.000 | 0.614 |
| The results are presented as the mean difference for each group and the mean difference between the groups. Data were analysed using restricted longitudinal data analysis (cLDA).  Bold letters indicate significant differences.  ^Ψ^The percentage change (corresponding assessment less baseline values/baseline values × 100) was calculated.  *Abbreviations*: CI, confidence interval; BMI, body mass index; WC, waist circumference; BMC, body mass content; aBMD, areal body mass density. | | | | | | | | | |

# **Figure ESM1.** Actual progression of the Diactive-1 muscle strength training programme over the 24-week period.

**
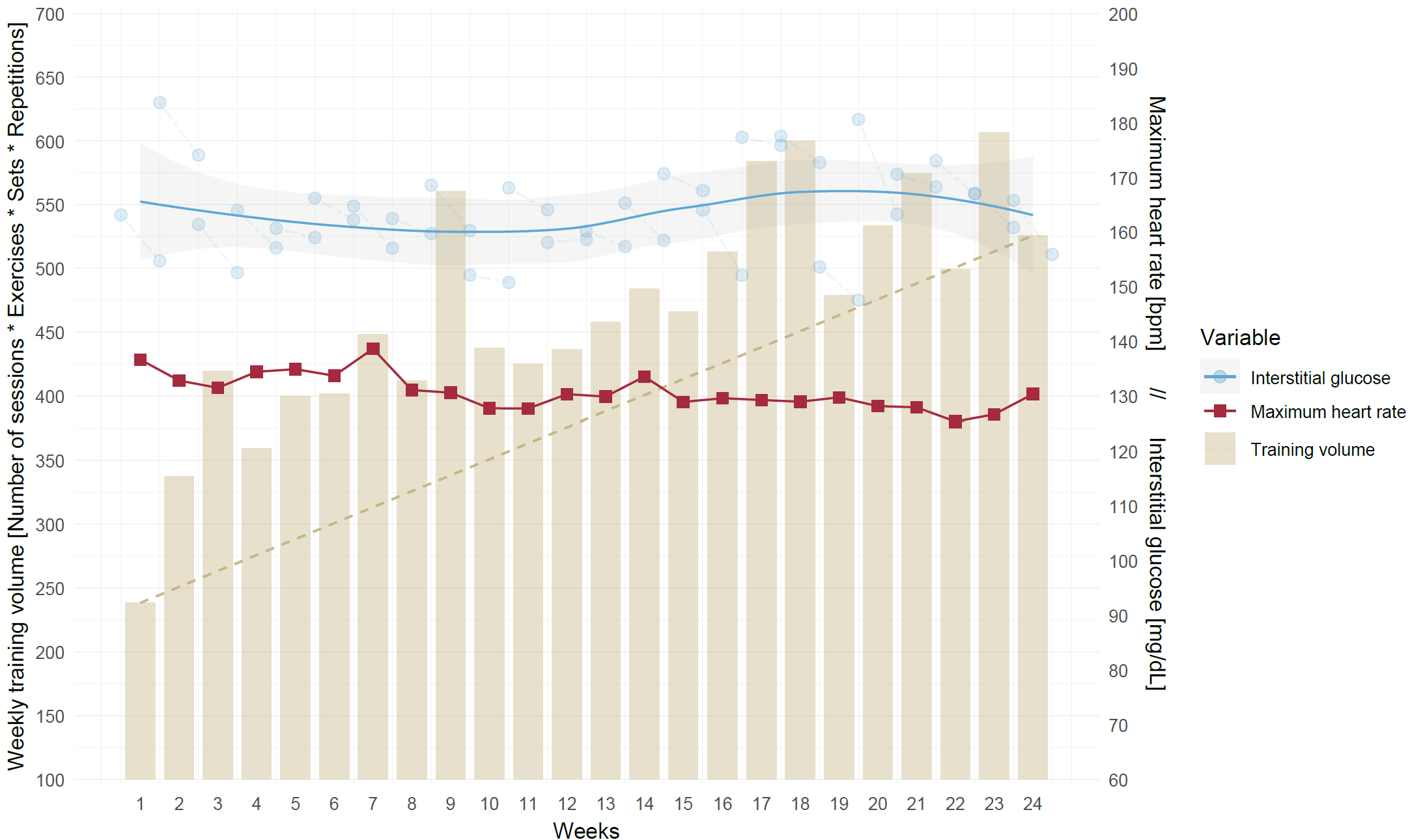
**

In the figure, earth-toned bars represent the quantified weekly training volume (i.e. weekly sessions × number of exercises executed in each session × sets completed in each exercise × repetitions performed in each set), while the dashed line indicates the difference of volume training between the first and last week. The blue smoothed line depicts the mean inter-training blood glucose levels recorded, whereas the blue dots connected by dashed lines represent pre- and post-training blood glucose measurements. The red squares, linked by red dashed lines, indicate the mean weekly maximum heart rate recorded, serving as a measure of adherence to training sessions.

# **Figure ESM2.** Algorithm for sarcopenia status classification according to EWGSOP2 criteria.

Muscle strength

[ > P20 ]

Muscle strength

[ ≤ P20 ]

ALMI

[ > 1.5 SD ]

Muscle strength

[ ≤ P20 ]

ALMI

[≤ 1.5 SD ]

Without sarcopenia

Probable sarcopenia

Confirmed sarcopenia
